# Laser diagnostic investigation on flame-assisted spray synthesis of NMC811 battery materials

**DOI:** 10.1038/s41598-025-26673-y

**Published:** 2025-11-27

**Authors:** Yutao Zheng, Jili Wei, Lee Weller, Simone Hochgreb

**Affiliations:** Department of Engineering, University of Cambridge, Cambridge, CB2 1PZ United Kingdom

**Keywords:** Chemistry, Energy science and technology, Engineering, Materials science, Physics

## Abstract

There is significant interest in the development of robust and simplified processes for the production of battery materials, including Li(Ni$_{0.8}$Mn$_{0.1}$Co$_{0.1}$)O$_2$ (NMC811), which has demonstrated high energy capacity, thermal stability and excellent electrical conductivity. This study developed a flat-flame reactor to provide a flame environment for flame spray pyrolysis (FSP), starting from an aerosolised solution of a mixture of metal nitrates in water. Preliminary studies were conducted to confirm that the operating conditions produced suitable NMC materials with acceptable performance after annealing at 750 $^{\circ }$C. Multiple laser diagnostic techniques were applied to characterise the spatial distribution of reactants and products and capture the process of reaction. Phase Doppler particle analysis was used to capture the droplet characteristics of precursors, and Mie scattering was used to map the instantaneous spatial distribution of droplets. A range of excitation wavelengths was tested to detect the participating species. However, only the high-energy wavelengths below 400 nm were capable of eliciting any signal. Light from a 355 nm pulsed laser was used to excite phase-selective laser-induced breakdown spectroscopy (PS-LIBS) to characterise the spatial distribution of the synthesised particles arising from mixing and reaction in the high temperature zone. The excited emission from the reaction zone was spectrally characterised, as was the corresponding time signature. Finally, simultaneous Mie scattering and PS-LIBS images were obtained to capture both droplet and synthesised particle distribution, capturing the emerging reaction process. The studies show, for the first time, how emissions from the formed particles can be used as a surrogate for the progress of reaction in similar FSP systems.

## Introduction

Rechargeable batteries, particularly lithium-ion batteries, have become pivotal in the use of renewable, sustainable energy. The importance of cathodes in improving battery performance has become essential when a higher specific capacity and faster charging/discharging cycles are expected.

Lithium-nickel-manganese-cobalt oxide (NMC) materials are some of the most widely used materials, considering their high specific energy capacity and attractive cost^1^. In particular, nickel-rich cathode materials such as Li(Ni$_{0.8}$Mn$_{0.1}$Co$_{0.1}$)O$_2$ (NMC811) have attracted significant attention for industrial applications, as Ni-rich layer cathode allows for high theoretical energy density and ionic conductivity^2^.

To produce these battery cathode materials in an efficient, economical and environmentally friendly manner, various synthesis techniques have been applied, including co-precipitation^3^, sol-gel^4,5^, hydrothermal^6^, spray-pyrolysis^7,8^ and flame spray pyrolysis^9,10^. Some of these methods, including flame spray pyrolysis (FSP), provide a potential approach to produce NMC in a clean and high-throughput scalable manner. Investigations have shown FSP can yield single-crystal Ni-rich cathodes with superior structural stability^2^, yet also produces small and fragile particles which create lower cycling durability^11^. However, FSP requires that precursors be available in liquid form, produces agglomerates of less controllable quality, and requires additional long-term calcination and annealing to achieve the crystallisation of expected materials^12^.

Zang et al^9^. evaluated the techno-economic feasibility of producing NMC cathode materials via flame spray pyrolysis. Compared with a traditional carbonate co-precipitation pathway, they have shown that FSP could reduce the minimum sale price of cathode material by 17% and offer lower wastewater emissions. Zhang et al^13^. provided an example of the production of NMC811 cathode materials via FSP with the addition of urea to improve the process. Morphology, crystal characteristics and battery performance of calcinated particles were measured by SEM, XRD and electrochemical tests. The results of the electrochemical tests showed the capability of using flame spray pyrolysis to produce NMC materials that had equal battery performance compared with commercial materials^13^. The study also found that the addition of urea improved the uniformity of Li distribution and shortened the required calcination time after collection. Madero et al^10^. optimised a FSP reactor to enable low-temperature flame spray pyrolysis (LT-FSP) to produce NMC material. Li$_{1.2}$(Ni$_{0.54}$Mn$_{0.13}$Co$_{0.13}$)O$_2$ was synthesised under reactor temperatures of 800$\sim$1000 K and showed a good charging/discharging battery performance. A computational study comparing LT-FSP to a flame-assisted SP system (FSP) was also conducted to support the effect of uniform temperature distribution in LT-FSP on improving material performance.

There have been a number of studies targeting the process by which aerosolised precursors are transformed into oxides or NMCs, including measurements of temperature^14,15^, and emission studies of metal oxides^16,17^. Although Chrystie^18^ has pointed out the necessity of conducting laser diagnostic techniques on capturing the process of synthesis, especially in flame synthesis, there have been no in situ measurements on the local distribution of precursors and species affecting the process of flame spray pyrolysis for NMC synthesis. The main purpose of this study is to explore the possibilities of applying spectroscopic techniques to identify the spatial distribution of precursor and product surrogates in the synthesis of NMC811.

Recently, phase-selective laser-induced breakdown spectroscopy (PS-LIBS) was developed as an *in-situ* laser diagnostic technique for capturing the solid phase of synthesised particles^17,19,20^. PS-LIBS is atomic emission spectroscopy generated from laser-induced nanoplasma, which requires an energy intensity of the order 1 GW/cm$^2$ with a signal duration of the order of nanoseconds^21,22^. An Nd:YAG laser that can operate at the second (532 nm) or third (355 nm) harmonic modes can generally serve as the excitation source for PS-LIBS. The benefit of the PS-LIBS is the selective ionisation of the particle phase, which enables the flow structures of the particles to be imaged^23^.

In the present study, pulsed Mie scattering is used to capture spatial distributions of precursor solution droplets, and laser-induced emission from the solid phase, which we have associated with PS-LIBS, is used to capture spatial distributions of synthesised particles. The temporal and spectral characteristics of the PS-LIBS signal are captured to validate the type of signal being taken. Finally, Mie scattering and PS-LIBS were deployed *simultaneously* to understand the distribution of the droplets and synthesised particles. The aim is thus to apply multiple laser diagnostic methods to flame spray pyrolysis of NMCs, and investigate the physical process of spray evaporation and the emergence of synthesize particles process of cathode materials in a flame-assisted environment.

## Experimental methodology

Multiple laser diagnostic methods were employed to measure different characteristics in the reaction zone of a flame spray synthesis reactor. The 2D spatial distribution of incoming droplets is measured using scattered light from a shaped laser beam. The size distribution of droplets is measured using phase Doppler particle anemometry (PDPA). Finally, point-wise and planar PS-LIBS at 355 nm was used to capture the emission of the synthesized particles. Further details of the techniques are detailed in each section.

### Flame synthesis setup

**Fig. 1:**
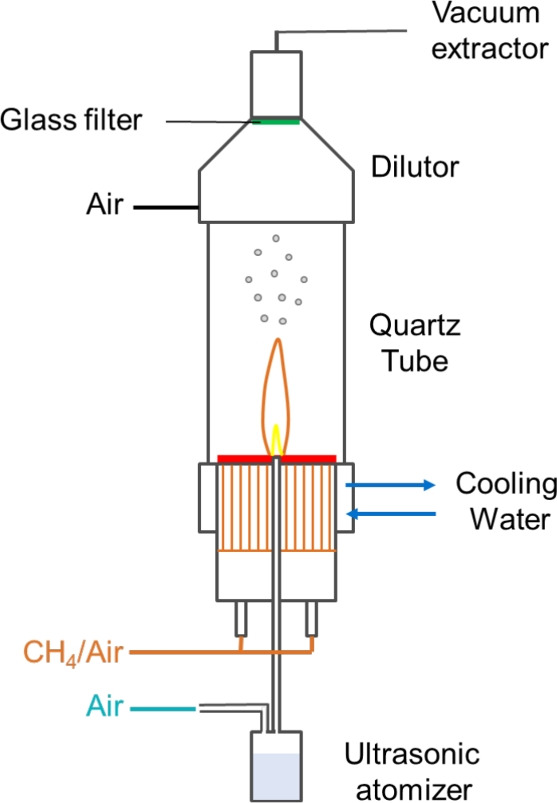
Flame-assisted spray pyrolysis system.

The flame-assisted spray pyrolysis system is shown in Fig. 1, consisting of three parts: the central region formed of a laminar jet of reactants, a surrounding flat flame burner which produces hot products, and a particle collection system. The inner jet consists of an inner and outer diameter ID/OD=10/13 mm quartz tube, through which air and aerosolised precursors flow. The flat flame is stabilised at a 14-mm thick ceramic perforated plate through which the jet tube protrudes 6 mm above the surface of the flat burner. The precursor droplets are generated by an jet atomiser (Omron C28P) and carried to the central zone by air through the central 10 mm tube. The mean droplet diameter was measured as 3 $\mu$m (PDPA, discussed in A) carried by an air flow rate of 10 slpm as metered by mass flow controllers (Alicat MC series 20 slpm with ±0.6% accuracy). The carrier air provides plenty of excess oxygen (>100 times) for oxidation of the incoming metal precursors^24^.

The outer flame uses methane and air premixed in proportions of 2.85 and 27.15 SLPM, respectively, to produce a stoichiometric ($\phi =1.0$) laminar flat flame, with adiabatic temperature of ($T_{ad}=2170$ K), stabilized above a porous ceramic plate with internal/external diameters of ID/OD = 14 mm/100 mm. The stoichiometric condition was chosen to deliver a higher gas temperature to minimise the necessary calcination time. Nevertheless, as shown elsewhere, much longer calcination times (hours) are still necessary to produce materials of sufficient quality for batteries^13,24^. An outer $D=$100 mm quartz tube shown in Fig. 1 was used to shield the reacting region during the long-term synthesis of sample collection but removed during the application of laser diagnostics.

The precursor solution was prepared by dissolving metal nitrates ($\ge$97% purity, Sigma-Aldrich), including lithium nitrate ($\mathrm LiNO_3$), nickel nitrate hexahydrate ($\mathrm{Ni(NO_3)_2 \cdot 6H_2O}$), manganese nitrate hexahydrate ($\mathrm{Mn(NO_3)_2\cdot 6H_2O}$)), and cobalt nitrate hexahydrate ($\mathrm{Co(NO_3)_2\cdot 6H_2O}))$ in pure deionized water, with molar ratios of 1.1:0.8:0.1:0.1 for NMC811 synthesis, shown in Table 1. The molar concentration of Li was 0.55 mol/L, corresponding to 10% in excess of stoichiometry, to compensate for the loss of Li during the synthesis process. The main source of lithium loss was the decomposition of lithium carbonate that failed to react with the nickel oxide structure, while another possible reason for the loss can be the thermal decomposition of Li$_x$Ni$_{2-x}$O$_2$^25^. Flow rates of precursors of the experimental test case are provided in Table 1.

**Table 1:** Precursor flow rates and flame setup.

Variable	Content	Unit	Values
Precursor	LiNO$_3$	mol/L	0.55
	Ni(NO$$_3)_2$$	mol/L	0.40
	Mn(NO$$_3)_2$$	mol/L	0.05
	Co(NO$$_3)_2$$	mol/L	0.05
$\dot{Q}_{p}$		ml/min	$\sim$0.50
$\dot{Q}_{air}$		slpm	10

$\dot{Q}_{p}$: total volumetric flow rate of precursors and deionized water...

$\dot{Q}_{air}$: flow rate of the central carrier air at standard pressure and temperature conditions...

To validate the feasibility of producing NMC811 material with useful battery performance, the synthesised particles produced by flame spray pyrolysis in the above setup were annealed into NMC crystal structures and tested as battery cathodes in a half-cell.

The present study is primarily focused on the results of the optical diagnostics of the process flow. Sample characteristics were only used to confirm that suitable properties were produced in the selected FSP process. Results of the materials and battery performance characteristics of synthesised particles and annealed particles are presented in B.

### Mie scattering: droplet spatial distribution

**Fig. 2:**
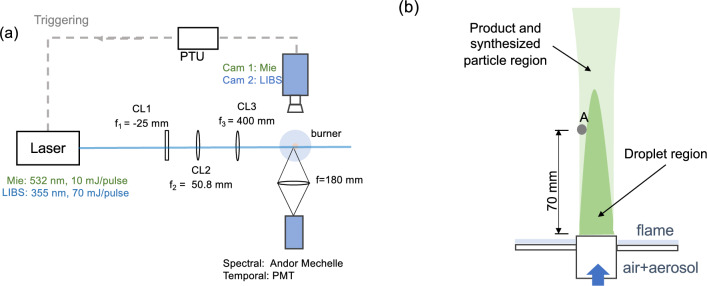
Optical arrangement for imaging of Mie scattering using 532 nm, and planar and pointwise LIBS using 355 nm excitation. The setup is timed by the PTU clocked by the laser. (**a**) Top-view of the optical arrangement; Camera 1: Andor iXon camera for Mie scattering; Camera 2: Nanostar intensified camera for LIBS. (**b**) Side-view of reacting zone across centreline. A: location of pointwise PS-LIBS measured by Andor Mechelle 5000 and PMT with 355 nm excitation.

A Nano L PIV laser was used to deliver 10±0.5 mJ/pulse at 532 nm wavelength. The laser beam was shaped into a sheet of 25 mm height $\times$ 0.1 mm thickness at the central plane of the reactor by using one concave and two convex lenses, as shown in Fig. 2. The beam exiting the laser head (diameter $\approx$ 4 mm) was expanded by a plano-concave cylindrical lens ($f_1$ = −25 mm) and reshaped by a plano-convex cylindrical lens ($f_2$ = 50.8 mm) into a 50 mm height $\times$ 4 mm thickness laser sheet. Finally, the laser sheet was focused by a planoconvex cylindrical lens ($f_3$ = 400 mm). The final laser sheet was clipped at the edges to create a nearly top-hat intensity profile with a remaining height of 25$\times$ 0.1 mm for the Mie scattering measurement. A knife edge was used to obtain the energy profile at the middle point and both sides of the edge. The energy intensity ratio was around 0.7(bottom):1(middle):0.7(upper), which led to a slight non-uniformity of the signal intensity vertically.

Timing was controlled by the DaVis LaVision processor timing unit (PTU), externally triggered at 10 Hz, and issuing a pulse every third image, for compatibility with the slowest camera detailed below, operating at 10/3 Hz.

The Mie-scattered light was captured by an unintensified Andor iXon Camera (Camera 1), using a lens with a focal length of 105 mm and a $532\pm 10$ mm band-pass filter. The imaged region is captured by a sensor of 1024 $\times$ 1024 pixels, with a nominal pixel resolution of 25 $\mu$m/pixel. The camera shutter time was set to the minimum possible of 20 $\mu$s. Five background images were subtracted with the flame on and droplets off. Mie scattering images were obtained repetitively at different heights by tuning the height of the flame, and 500 images were collected for each 25 mm tall region, spanning the height above the burner exit (HAB) from 20 to 120 mm.

### Phase-selective laser-induced breakdown spectroscopy

A preliminary series of tests was performed by scanning a laser beam produced by an optical parametric oscillator system (Coherent Horizon, tunable wavelength from 192 to 2750 nm), pumped by a tripled harmonic beam (355 nm) of a Nd:YAG laser (Coherent Surelite-III) with a pulse energy of 130 mJ, over a control volume in the area of point A in the diagram (Fig. 2) corresponding to the product region, and collecting the resulting light using a spectrometer. The scan tests showed no signal above the background baseline, except for excitation near and below the level of 355 nm, and then, the excitation seems not to be sensitive to the wavelength. Therefore, we have chosen to use light from a 355 nm beam by using a tripler on a 1064 nm laser to enable imaging of the selected synthesised region.

#### Laser setup

The same pulsed Nd:YAG laser (Coherent Surelite-III) with a wavelength tripler delivering 355 nm wavelength light provided approximately 130 mJ/pulse at a fixed repetitive rate of 10 Hz to produce a laser sheet across the reactant and product region. The 4 mm diameter beam was shaped into a sheet of $\sim$0.1 mm $\times$20 mm using the same group of lenses described above, one plano-concave ($f_1=-25$ mm) and two convex UV-lenses ($f_2=50.8$ mm and $f_3$ = 400 mm). The position of the second lens ($f_2=50.8$) was slightly tuned to modify the final sheet into 20 mm height, which was clipped into the desired size ($\sim$ 0.1 mm $\times$ 12 mm) by blocks, with a final energy of 70 mJ per pulse, corresponding to 0.972 GW/cm$^2$. By moving the reactor vertically, the 12 mm height frame was moved to scan the resulting signal of the synthesised particles from the bottom to the tip of the region in Fig. 2.

In all following experiments, including a local measurement for the spectral and temporal characteristics and a 2D PS-LIBS measurement for spatial distribution, the 355 nm laser setup was kept the same for a consistent configuration.

#### Local pointwise spectrum and signal duration measurement

The emission from the reaction region named in Fig. 2(b) as point A was excited by a 355 nm laser beam, using a spherical convex lens with focal length $f=180$ mm. The resulting emitted spectrum from the target region (a cylindrical spot with a diameter of 3 mm and a thickness of the laser sheet$\sim 0.1$ mm, calibrated by a fibre-based bulb) was measured using a high-resolution, wide-range Andor Mechelle 5000 spectrograph (200–975 nm calibrated by a series of fixed-wavelength light sources). A total of 200 spectral shots were hardware-accumulated onto a single spectrum in the range of 300–900 nm, after subtracting baseline emissions taken with flame on, but laser excitation off.

Time characteristics of the signal at selected wavelengths were measured from the laser pulse arrival time using a photomultiplier module (PMT, Hamamatsu, H10721-20), outfitted with a variable voltage for amplification (Hamamatsu, C10709, 2.8 to 5.0 V). Different filters ($450\pm 20$ nm and $670\pm 10$ nm) were used to collect specific ranges of luminescent excited emissions, once the peaks in the spectrum were identified. A silicon photodetector (Thorlabs, DET10A), was used as a trigger for the light from the pulsed laser to provide a reference time and trigger for the PMT signal, both collected using an oscilloscope (Tektronix DPO, 100 MHz, 2.5GS/s). A pulse detector, Thorlabs DET10A (1 ns rise time), was used to capture the residence of the 355 nm laser beam as a reference time by pointing it towards the laser source.

#### 2D PS-LIBS imaging

A Nanostar intensified CCD (ICCD) (Camera 2) camera was used to detect the emission from the reacting zone at a recording frequency set to 10/3 Hz (limited by the camera chip acquisition rate). A 400 nm long-pass filter was set in front of the camera lens. The ICCD sensor was not sensitive to signals with wavelengths larger than 600 nm, so the wavelength range collected was in the range of 400 to 600 nm. The visible lens objective with a focal length of 60 mm was doubled to 120 mm by adding a teleconverter, with a maximum aperture of *f*/2.8 mm. The imaged region was collected over 1280 $\times$ 1024 pixels, with a pixel resolution of 23.4 $\mu$m/pixel, covering a height of 24 mm by a width of 30 mm. Five background images were acquired at steady laminar conditions, without the addition of precursor droplets and averaged to be subtracted using the Nanostar ICCD camera. The background electronic noise was found to be negligible compared to the fluctuation in the signal. The camera intensifier was gated over 10 $\mu$s, starting at the time when the 355 nm laser was excited, and the gain ratio was set to 30/99. A total of 1000 images were collected stepwise for every 12 mm height above the burner (HAB), from HAB=20 to 120 mm.

### Simultaneous 2D Mie/PS-LIBS

**Fig. 3:**
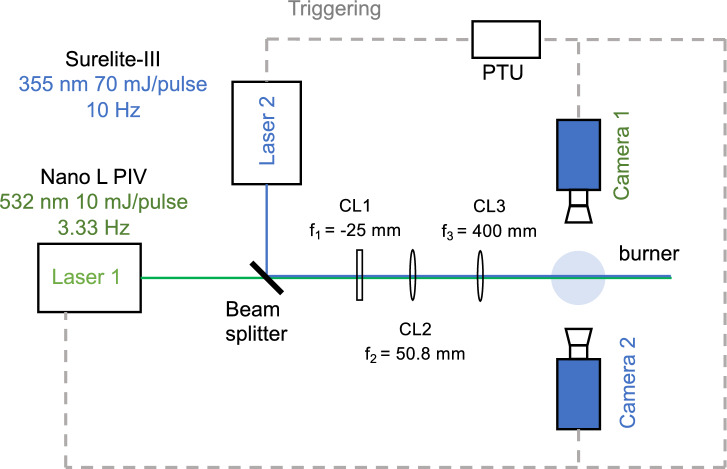
Experimental setup for simultaneous Mie scattering and PS-LIBS signal collection, including triggering scheme for two cameras and Nano L PIV laser, using the clock master from the Surelite laser CL1: concave cylindrical lens; CL2 and 3: convex cylindrical lens. Camera 1: Andor iXon camera for Mie scattering; Camera 2: Nanostar camera for PS-LIBS.

The setup in Sec. "Phase-selective laser-induced breakdown spectroscopy" for PS-LIBS measurements was combined with the Mie scattering setup described in Sec "Mie scattering: Droplet spatial distribution" to deliver simultaneous measurements described in Fig. 3. An Andor iXon camera (Camera 1) was used to capture Mie scattering at 10/3 Hz as described above, with a $532\pm 10$ mm band-pass filter in front of the camera. The Nanostar ICCD camera (Camera 2) was used to capture the PS-LIBS signal at 10/3 Hz. A 500 nm short-pass filter was set in front of Camera 2 to exclude the Mie scattering signal.

The 355 nm Surelite-III laser acted as a master to trigger Cameras 1 and 2, as well as the Q-switch timing of the Nano L PIV laser. The Surelite laser provided 10 Hz 355 nm laser pulses and triggered the processor timing unit to send a new triggering signal every third pulse at 10/3 Hz. A Lavision Programmable Timing Unit (PTU) was used to conduct the signal synchronisation and take 48 pairs of images for each run at different HABs.

## Results

### Spectral and temporal measurements

**Fig. 4:**
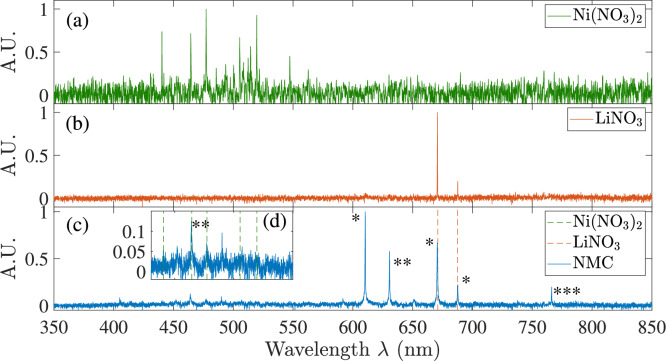
High-resolution spectral measurements of light intensity obtained at location ’A’ (Fig. 3(b)) for 355 nm-excited emission in the range of 350 to 850 nm. Emission spectrum for nickel nitrate solution (**a**); lithium nitrate (**b**); mixed NMC precursor with dashed lines corresponding to peaks in two nitrate solutions (**c**). (**d**) inset: detail of emission for NMC solution in the wavelength region of 420$\sim$550 nm; green and red dashed lines: peaks corresponding to top and middle plots, respectively. Markers *: Li I; **: Ni I; *** Co II.

Figure 4 presents the results of spectral measurements for excitation at 355 nm, collected at location ’A’ in Fig. 2(b). The background emission was measured to be negligible without excitation at 355 nm. Five spectra were acquired and averaged with droplets on and laser off for each condition as the background, and subtracted in the data processing procedure. No baseline broadband spectrum was detected. This allows the exclusion of either bremsstrahlung radiation^26^, as well as laser-induced incandescence.

The intensity measured in each subplot was normalised separately to its own maximum for visibility. To characterise the likely source of these two groups of peaks, two reference measurements were conducted by using single metal nitrate solutions, namely lithium nitrate and nickel nitrate. The emission spectra for the two reference solutions are plotted in Fig. 4 for (nickel)(a) and (lithium)(b) nitrate. Dashed lines with corresponding colours are also plotted in the emission spectrum of the NMC solution for ease of comparison. The overlap between coloured dashed lines and peaks in the emission of NMC precursor in Fig. 4 shows that the excited lines coincide with the spectra produced by Li and Ni individually. This is expected, as Li and Ni are added to the solution in a proportion of 55:40 (Table 1) and have larger proportions than other metals’ proportions in synthesised particles. The spectral location of peaks for Ni in the range of 440 to 550 nm is supported by previous experimental measurements^27–29^. The three peaks at 610, 670 and 690 nm are associated with Li atomic lines^30^. Oddly, although the latter peaks do appear in the pure lithium nitrate experiment, the extra peaks at 610 and 630 nm appear only in the NMC plots (Fig. 4(c)), even though those are both two strong lines of Li and Ni atomic spectroscopy^31^.

**Table 2:** Center wavelength and FWHM of the detected ionic spectral lines compared with the NIST database^30,32^.

Ni(NO$_3$) (nm)$_2$	LiNO (nm)$_3$	NMC (nm)	FWHM (nm)	Ion (nm)	Ritz wavelength (NIST) (nm)
440.3			0.29	Ni I	440.154
		464.2	0.8	Ni I	464.865
464.7			0.28	Ni I	464.865
477.2		477.2	0.38	Ni I	477.341
		490.1	0.20	Ni I	490.097
505.1			0.50	Ni I	504.885
514.4			0.39	Ni I	514.277
519.6			0.28	Ni I	519.716
547.3			0.40	Ni I	547.690
		610.3	0.43	Li I	610.366
		630.6	0.41	Ni I	631.466$^{(1)}$
	670.7	670.7	0.35	Li I	670.791
	687.3	687.4	0.62	Li I	687.308
		766.1	0.44	Co II	766.236

$^{(1)}$closest lines corresponding to potential ions in precursors.. .

Two groups of peaks were detected in the green (430$\sim$550 nm) and red wavelength regions (610$\sim$700 nm) and listed in Table 2. The centre wavelength and FWHM of the major peaks were compared with potential lines (the rightmost column) extracted from the NIST database^30,32^. Based on observed wavelengths and the proportion of the NMC (Li, Ni, Mn, Co), the wavelengths were estimated for the potentially released ions, according to the Rydberg-Ritz wavelengths closest to observed wavelengths, and the estimated line origins are identified and indicated directly on Fig. 4. The signal in the green region (400$\sim$550 nm) appears to be much smaller than that in the red region (610$\sim$670 nm), which is consistent with the relative intensity of Li (3600 at 670 nm) and Ni($<110$ at 440$\sim$547 nm) on the NIST database. We note that the intensified camera used for imaging in the next section is optimised for the lower wavelengths and is unable to detect signals in the longer range for imaging purposes.

**Fig. 5:**
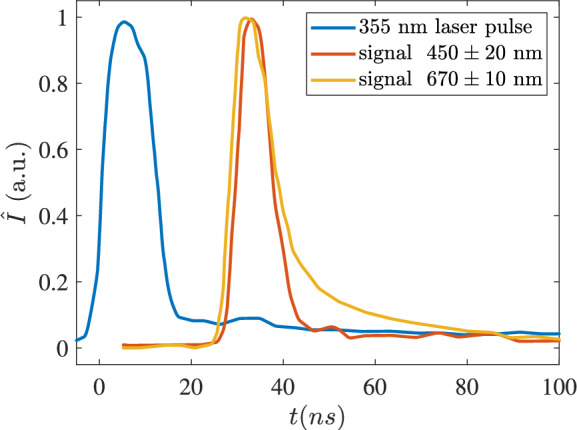
Time history of excited emission measured at two different wavelengths, relative to the arrival time of the exciting 355 nm laser beam. Blue: normalised time history for 355 nm collected by photon detector; red and orange lines: normalised signal intensity obtained at two different filter wavelength ranges, as collected by the PMT.

Given the high energies per pulse used (6 J/cm$^2$, 0.972 GW/cm$^2$ estimated by the FWHM of the laser pulse in Fig. 5), it is suggested that nanoparticles undergo ablation into free atoms. Upon direct absorption of laser photons, electrons are excited into the conduction band, initiating the ablation process and resulting in the formation of nano-plasmas. However, under these excitation conditions, impact ionisation of the surrounding gas phase is unlikely to occur, as this process typically requires a more energetic wavelength^33^. Consequently, this enables a phase-selective breakdown^21^. The phase-selective signals are shown in the next section, where the instantaneous contour of the droplet region is captured.

A comparison with database candidate lines was able to identify the origin of the observed emissions, with the exception of 630.6 nm lines from potential metal lines of NMC precursors. The deviation of 0.9 nm between this observed line and the closest line for Ni at 631.466 nm is significantly larger than the resolution of the Andor Mechelle 5000 spectrometer (0.1 nm) and the FWHM of the detected peak (0.41 nm). Further studies are clearly necessary to clarify the identity of this extra line.

The emitted signal duration was measured for the full NMC solution case by a Hamamatsu PMT over selected frequency ranges, as presented in Fig. 5. The emitted signal in the green and red wavelength regions was collected by the oscilloscope over two different wavelength regions centred at 450 and 670 nm, respectively, in two different sets of experiments. Signal lifetimes are of the order of the laser excitation lifetime, which is consistent with the excitation/de-excitation of electronic levels, and does not support the hypothesis of laser-induced incandescence, which is typically broadband and longer lived (hundreds of nanoseconds) for the expected nano-sized particles^19^.

**Fig. 6:**
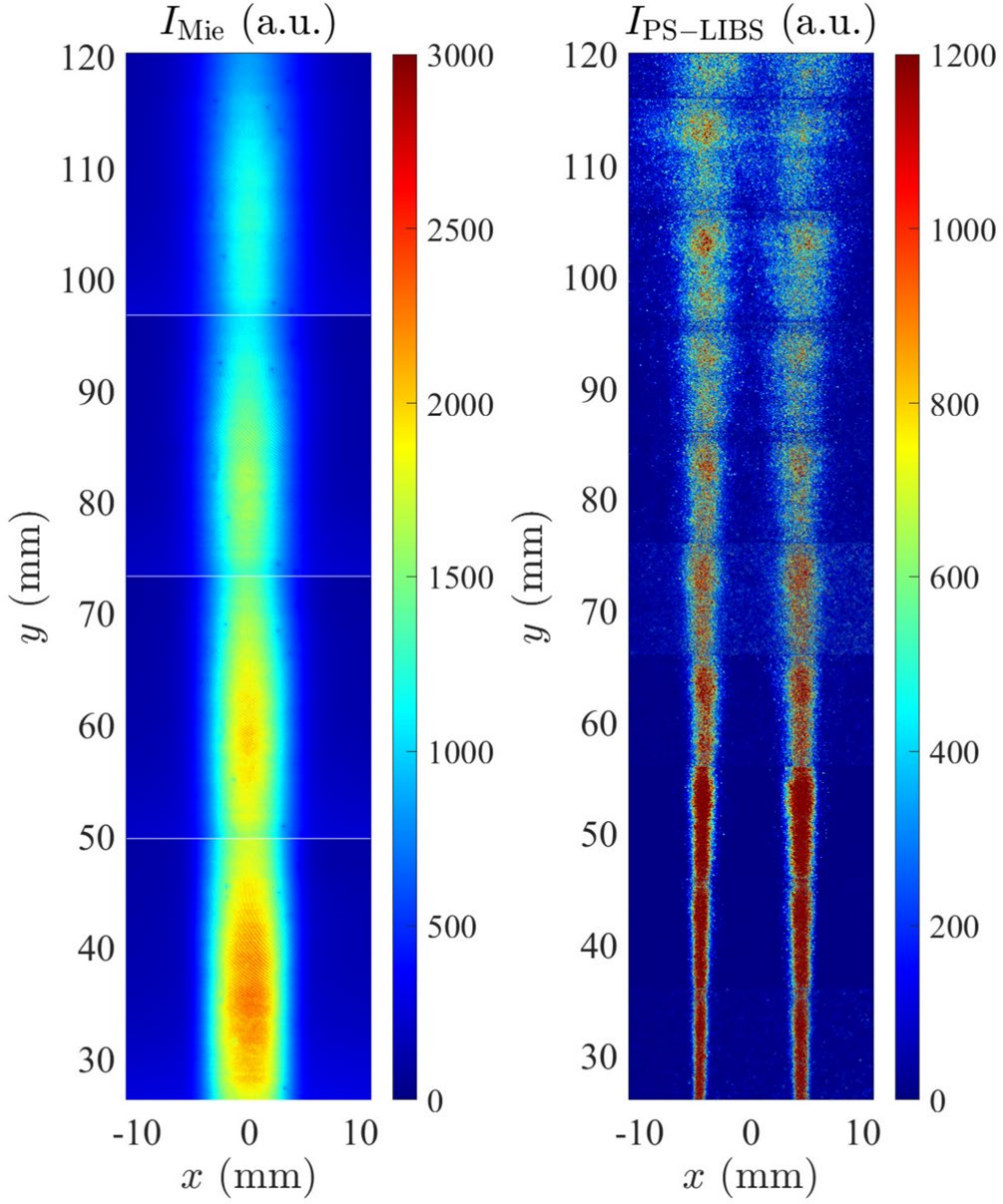
Composite ensemble average of images between HAB$=25\sim 120$ mm. (**a**) Mie scattering images, (**b**) PS-LIBS emission over 400 to 600 nm.

Figure 6 shows a composite of the ensemble averages of Mie scattering and PS-LIBS images acquired *separately*. Fig. 6(a) shows the ensemble average of the 1000 Mie scattering signal images taken within four experiment runs where each run captured 200 images for a region of $25\times 25$ mm. The Mie scattering images capture the spatial location of non-vaporised solution droplets. The Mie scattering intensity is corrected by dividing by the average relative energy profile calibrated in advance. Mie scattering signals are often scaled with the number of particles and the square of the particle diameter in the relevant size range. It is expected that evaporation takes place at the edges of the droplet column, decreasing both the number and size of droplets until they disappear at the top edge.

Conversely, Fig. 6(b) shows the ensemble average of the PS-LIBS images acquired for 10 identical experimental runs, where each run captured 1000 images for a region of 25 mm wide and 12 mm height, using a 400 nm long-pass filter. The narrow height band was necessary to allow sufficient energy fluence for detectable signals from PS-LIBS. The beam profile of the incoming light is not perfectly flat, leading to the striped behaviour on the edge. The images represent the signal obtained from nickel emission, as indicated in Fig. 4. The composite images show that the signal is largest just outside of the droplet vaporisation region: the precursor reactants are formed, either from calcined droplets or vaporised precursors, leading to PS-LIBS signal from the atomic lines associated with the metal, or possibly their oxides. The behaviour of simultaneous single-shot distributions of Mie and PS-LIBS signals is discussed in the next section.

### Simultaneous Mie/PS-LIBS results

**Fig. 7:**
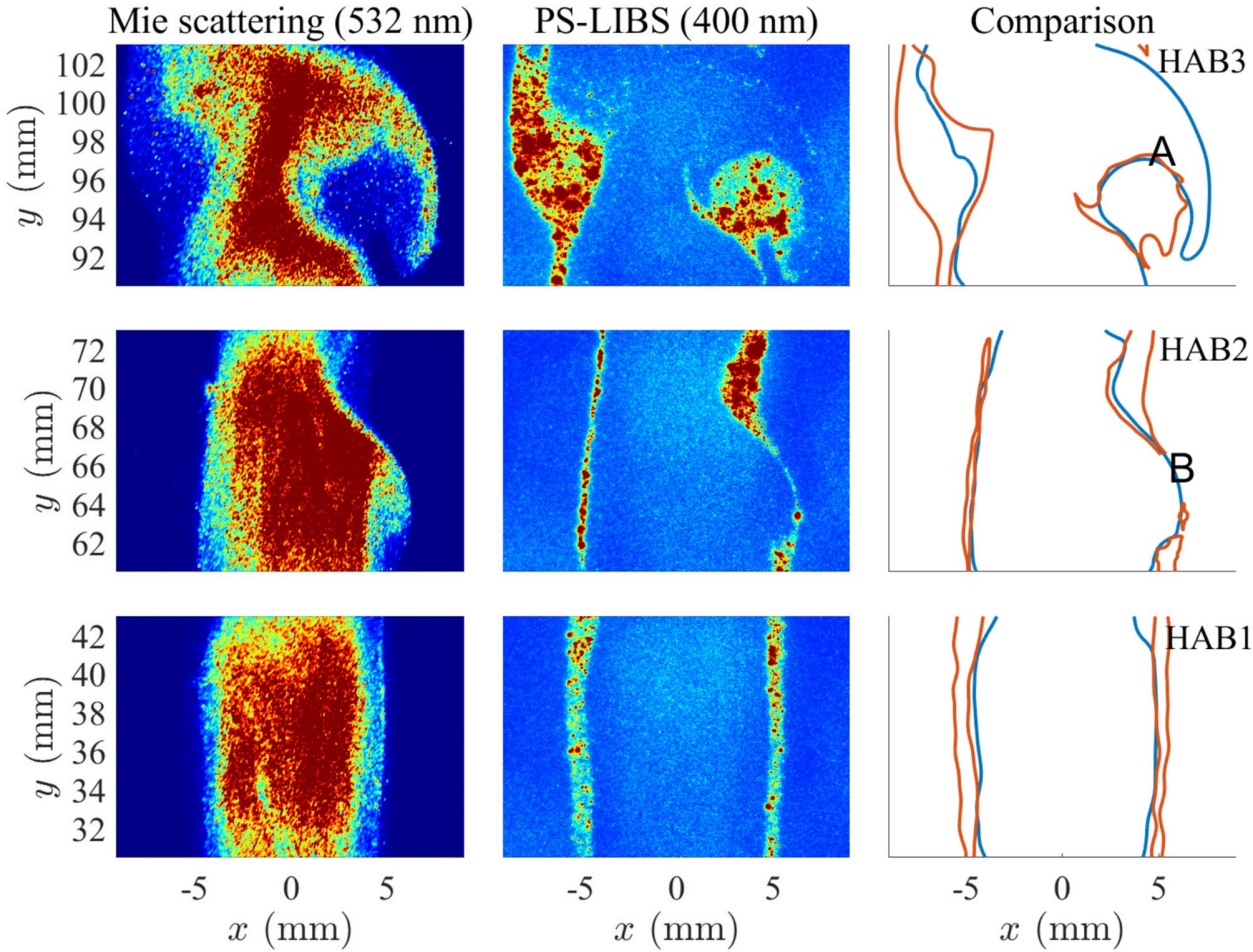
Simultaneous images indicating Mie scattering and PS-LIBS images at three different heights. Left: droplet Mie scattering signal characterised (532 nm); middle: PS-LIBS signal (400–600 nm); right: iso-contours (10 and 90%) for Mie scattering (blue) and PS-LIBS (red).

Figure 7 shows simultaneous images of Mie scattering (left) from incoming droplets and PS-LIBS signal (middle) associated with synthesised particles, for three different non-overlapping heights. Contours of the 10% (of the global maximum signal) region for Mie scattering (blue) and the 10% contours (red) of PS-LIBS are extracted after applying a 8$\times$8 px$^2$ moving mean filter for smoothed contours.

The results show that PS-LIBS signals are only observed in the outer zone of the Mie scattering region, a pattern which can also be found in all 48 pairs of images for each height provided in the supplementary documents. This is consistent with the vaporisation and synthesis of the PS-LIBS-containing particles or vapour. There is no clear separation between the droplet region and particle region: once droplets are vaporised, synthesised particles are formed. At the bottom two heights (HAB1 and HAB2), the flow is laminar and the reaction layer containing particles has a thickness of the order of 1 mm. This suggests that the particles are formed, and do not diffuse very quickly, giving rise to a layer of incipient product particles from which the signal arises. At the higher HAB3, a flow instability arises owing to the velocity and density difference between the inner and outer flows, leading to the formation of vortices engulfing the high-temperature particle formation zone. For example, region A in Fig. 7 contains particles formed at the interface, which accumulate in a region that coincides with the negative of the droplet pattern, as the vortex wraps around and forms a recirculation zone. Recirculation zones are associated with longer residence times for the particles, which do not leave the region; we observe that there is evidence of particle clumping, suggesting agglomeration owing to higher residence time. Conversely, comparing the pattern of region B with low droplet concentration and a thin layer of particles indicates a region where the inner layer is expanding, and no recirculation zone appears, only a thin layer of particles. This is a familiar pattern from soot formation in flames^34^, and the process here appears to be similar.

## Conclusions

A flame spray pyrolysis reactor has been built for producing battery materials of NMC811 starting from aerosolised droplet precursors delivered in a stream of micrometre-sized droplets intersecting high-temperature gases provided by the flame. Optical detection and imaging using Mie scattering of droplets and PS-LIBS of synthesised products have been employed to identify the spatial distribution and emission characteristics. The materials produced had been shown to have suitable electrochemical properties to work as Li-ion battery cathodes.

The measurements show how droplets disappear as they cross the flame, giving rise to PS-LIBS from the metals released in the process. A 355 nm blue laser was used to excite PS-LIBS of different metal ions, including well-defined lines identified with Ni and Li atoms, over the visible range, and offering lifetimes of the order of 10 ns.

Simultaneous Mie/PS-LIBS measurements of droplets and products showed that the particle products form immediately once in contact with the hot gases. A wider spatial distribution arises in regions where hot gases are entrained, suggesting that longer local residence times lead to the production and/or agglomeration of synthesis particles.

The present work has shown that it is possible to apply an image detection technique for particle formation associated with metal-containing species for FSP, clarifying where the products are formed and distributed, starting from initial aerosolised droplets. Significant further work is necessary to understand the details of particle calcination and stimulated emissions.

## Supplementary Information

Below is the link to the electronic supplementary material.

Supplementary material 1

## Figures and Tables

**Fig. 8:**
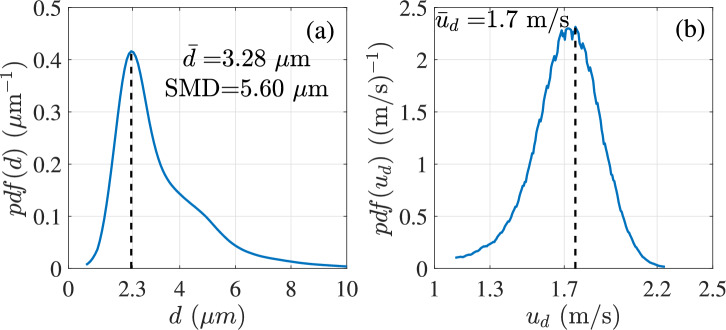
Measured pdf of droplet sizes (**a**) and droplet velocities (**b**) obtained 20 mm above the exit of the central tube.

**Fig. 9:**
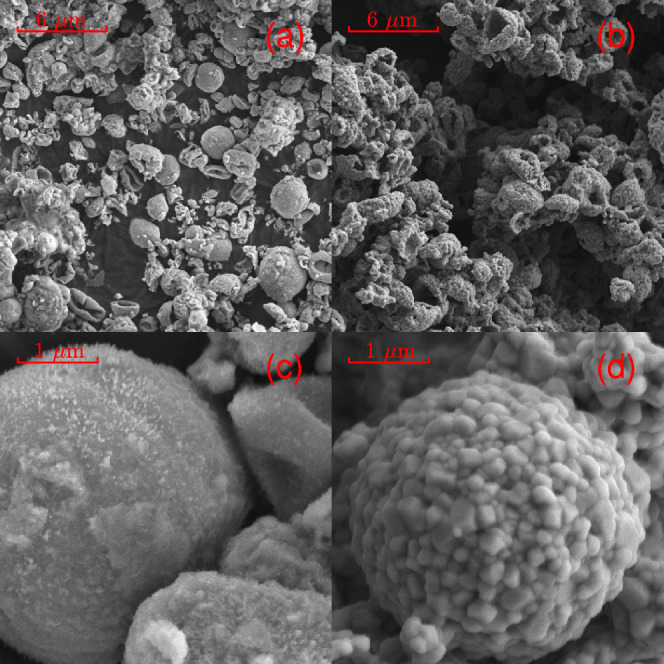
SEM images of synthesised samples of the case in Table 1 before and after calcination. Left column: synthesised particles before calcination (**a**) (**c**) magnified 6 times; right column: synthesised particles after calcination (**a**) (**d**) magnified 6 times.

**Fig. 10:**
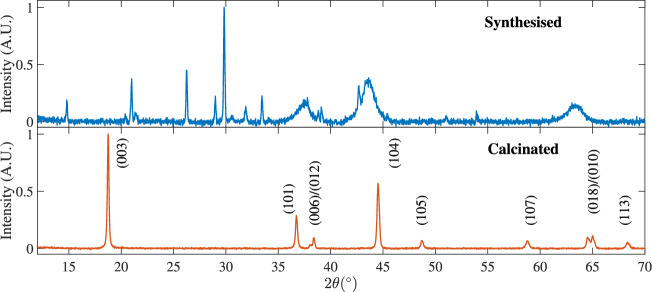
XRD results of the test case, including the synthesised sample and two calcinated samples.

**Fig. 11:**
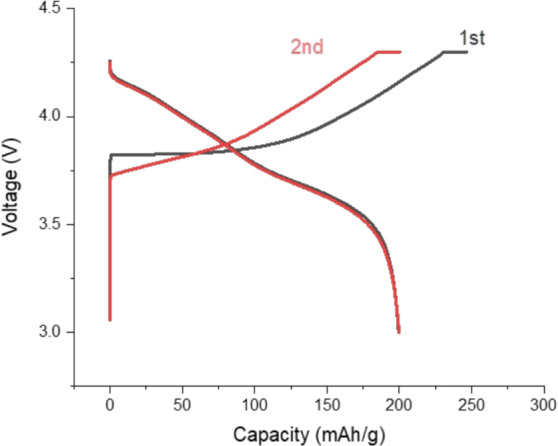
First and second cycle of the battery performance test.

## Data Availability

Data is provided within the manuscript or supplementary information files.

## A Phase Doppler Particle Anemometer (PDPA) measurements

Droplet measurements were undertaken at 20 mm above the exit of the central tube using a PDPA (Dantec Dynamics), deploying two crossed beams of a 514.5 nm cw laser at a 500 mm focal length transmitting probe. Data rates were of the order of $10^3$ to $10^4$ data points per second, which were sufficient to yield droplet size distributions.

Figure 8 shows the detected size distribution of droplet diameter pdf(*d*) and velocity pdf$(u_d)$ collected by PDPA above the exit under the mass flow rate of the jet in Table 1. The apparent mode of the diameter of droplets was around 2.3 $\mu$m, with a corresponding mean diameter (*d*1) of 3 $\mu$m and the corresponding Sauter mean diameter (SMD) of droplets of 5.60 $\mu$m. However, the *true* mode of the droplets is likely significantly smaller, as the sensitivity of the PDPA decreases quickly below 1 $\mu$m. This means that the SMD is not likely to be much affected, but the mode and first moment are somewhat overestimated. The measured mean velocity of droplets was around 1.7 m/s, which is slightly smaller than the mean bulk velocity of the gas phase (2 m/s). PDPA measurements downstream of the droplet region showed no significant signal, indicating that the synthesised particle diameters are too small to be measurable using the PDPA technique.

## B Sample characteristics and battery performance

Around 0.8$\sim$1 g synthesised micro-particles were collected by a glass fibre filter for 1.5 hrs (Whatman GF/F, 110 mm diameter), shown in Fig. 1. The collected material was placed in an alumina crucible and annealed in a tube furnace. The furnace temperature was increased to the target temperature at a ramping rate of 5 $^\circ$C/min to the final temperature (750 to 850 $^\circ$C), maintained for 9 h, after which the sample was removed and allowed to cool naturally.

Particle morphology and crystal characteristics were characterised before and after calcination using a scanning electron microscope (SEM), and elemental composition was obtained via energy-dispersive X-ray spectroscopy (EDX). The lithium signal could not be obtained given the limited voltage range of the beam (15 kV).

Phase identification of the immediately collected synthesised samples, and annealed samples were measured by X-ray diffraction (XRD). A Cu source was used, and the $k\beta$ was removed via a Nickel Filter before the detector, leaving only the $k\alpha 1$ (1.54060 Å) and $k\alpha 2$ (1.54443 Å) wavelengths. XRD results were processed by software GSAS-II to acquire crystal characteristics of the synthesised sample.

### B.1 SEM results

Figure 9 shows SEM images of the collected samples for the test case in Table 1 before calcination, shown at two different magnifications. Two different structures of samples arise, pure spheres and folded spheres. Prior to calcination, there are a number of smooth spheres of the order of 3 $\mu$m in diameter, a dimension close to the mode of maximum pdf of droplet sizes in Fig. 8. The molecular dynamic simulation work by Luo et al^35,36^. suggests that metal nitrates, except for Li, form an empty shell during calcination after the water evaporates, owing to differential diffusion, reaction and thermodynamic properties, and that is consistent with the observations. For calcinated samples, Fig. 9(b) shows that the larger spheres are coated with smaller crystal structures, probably lithium compounds that have precipitated. The shell in turn may inhibit the further evaporation of the remaining water, which may collapse to irregular shapes when particles pass through the flame.

### B.2 EDX results

Energy-dispersive X-ray spectroscopy (EDX) was used to measure the ratio of metal (Ni, Mn and Co) in the synthesised samples and calcinated samples, and compared with the target ratio of 8:1:1, shown in Table 3. In general, the measured ratio of Ni:Co:Mn is close enough to the expected ratio of 8:1:1 within an acceptable error. Table 3 also shows that the proportion of Mn appears to increase after the calcination process, which results from that the annealing process increases the Mn content at the surface by diffusion within the particle^37^.

**Table 3:** EDX results: Ratio normalised to Ni as 8, to compare with input 8:1:1 ratio.

	Before calcination			After calcination		
	Ni	Co	Mn	Ni	Co	Mn
ratio	8	1.04	0.93	8	1.06	0.99

### B.3 XRD results

XRD results for calcinated samples are able to reveal the crystal structures, such as the crystal size, levels of the mixing between different metals and mean *d*-spacing between different layers. Figure 10 shows XRD results of synthesised samples and calcinated samples for the test case. After 9 h of calcination, peaks of NMC811 begin to appear, implying the formation of a layered crystalline structure. Two peak pairs (006)/(012) and (018)/(110) split, suggesting cation ordering and a full lithiation process under high temperature.

The ratio of peaks *I*(003)/*I*(104) represents the cation mixing level of $\mathrm Li^+/Ni^{2+}$, and a higher ratio corresponds to a higher mixing level, leading to a more uniform layer distribution between $\mathrm Li$ and $\mathrm Ni$. Ni atoms can occupy the Li layer since $\mathrm {Ni^{2+}}$ and $\mathrm {Li^+}$ share similar atomic radii, resulting in the formation of disordered phases. $\mathrm I(003)/I(104)$ reaches 1.43 in the test case, which is an acceptable level of good battery performance^13,38^. A high value of peak ratio (003)/(104) means a low ion exchange level of $\mathrm {Ni^{2+}}$ and $\mathrm {Li^+}$ in layered structure metal oxides, which is conducive to good cycling performance.

Rietveld refinement was conducted to refine the crystal characteristics of calcinated samples through *GSAS-II*. Structural parameters of calcinated crystals calculated by using Rietveld refinement via *GSAS-II*^39^ are presented in Table 4. The XRD patterns in Fig. 10 show that the diffraction peaks of calcinated samples can be indexed based on the hexagonal $\alpha$-NaFeO$_2$ type, defining a metal oxide layered structure with an R-3mH(#166) space group with no impurity phases^40,41^.

**Table 4:** Crystal structural parameters calculated from Rietveld refinement of the XRD data of the calcinated sample.

Refinement characteristics		Value
Unit cell	a(Å)	2.869
	c(Å)	14.221
	c/a	4.957
Crystal size	($\mu$m)	0.108
Micro-strain$\varepsilon$	(10$^{-6}$)	614
$R_w$	(%)	2.80
GOF$^{(1)}$	/	1.77

$^{(1)}$ Goodness of fit..

The ratio of lattice parameters *c* to *a* (*c*/*a*) is commonly used to indicate the tetragonal distortion of the NMC samples^42^. A larger value of *c*/*a* indicates a better ordering of the hexagonal layered structure^43^, indicating higher purity^44^ and a better expected battery performance^13^.

### B.4 Battery performance

The charging and discharging process of the battery test has been conducted on the calcinated sample of the test case. The performance curves are shown in Fig. 11.

Three characteristics of battery performances for the Case in Table 1 in the first and second cycles with a charge/discharge rate of 0.1 C are listed in Table 5. The charging and discharging capacity of the first order has reached the level of a commercial NMC811 battery^45,46^. However, the Coulombic Efficiency (CE), defined as the ratio of discharging capacity to charging capacity, is only 80.9%. The low CE value is dominated by the slow Li-ion diffusion kinetics of the H1 phase in highly lithiated states^47^, which is mainly caused by insufficient lithium vacancies and narrow layer space. Therefore, the working voltage of the NCM811 electrode quickly reaches the cut-off voltage at the end of discharge, and there is not enough time for the full recovery of the H1 phase, leading to a loss of discharge capacity and low initial CE.

Nevertheless, the battery performance is sufficient to show the feasibility of producing NMC electrode materials with performance comparable with commercial materials, which show charge capacity of the first cycle in the range of 195$\sim$206 mAh/mg^45,46,48,49^. Further analysis using laser diagnostics is representative of future FSP processes.

**Table 5:** Battery performance of the test case.

Cycle	Charge capacity	Discharge capacity	Coulombic efficiency (CE)
	(mAh/mg)	(mAh/mg)	(%)
First	246.54	199.46	80.90
Second	200.49	199.72	99.61
