# Supplementary figures and images for: Laser diagnostic investigation on flame-assisted spray synthesis of NMC811 battery materials
Source: Sci Rep. 2025 Nov 27;15:42426. doi: 10.1038/s41598-025-26673-y (PMC12660942; doi:10.1038/s41598-025-26673-y)

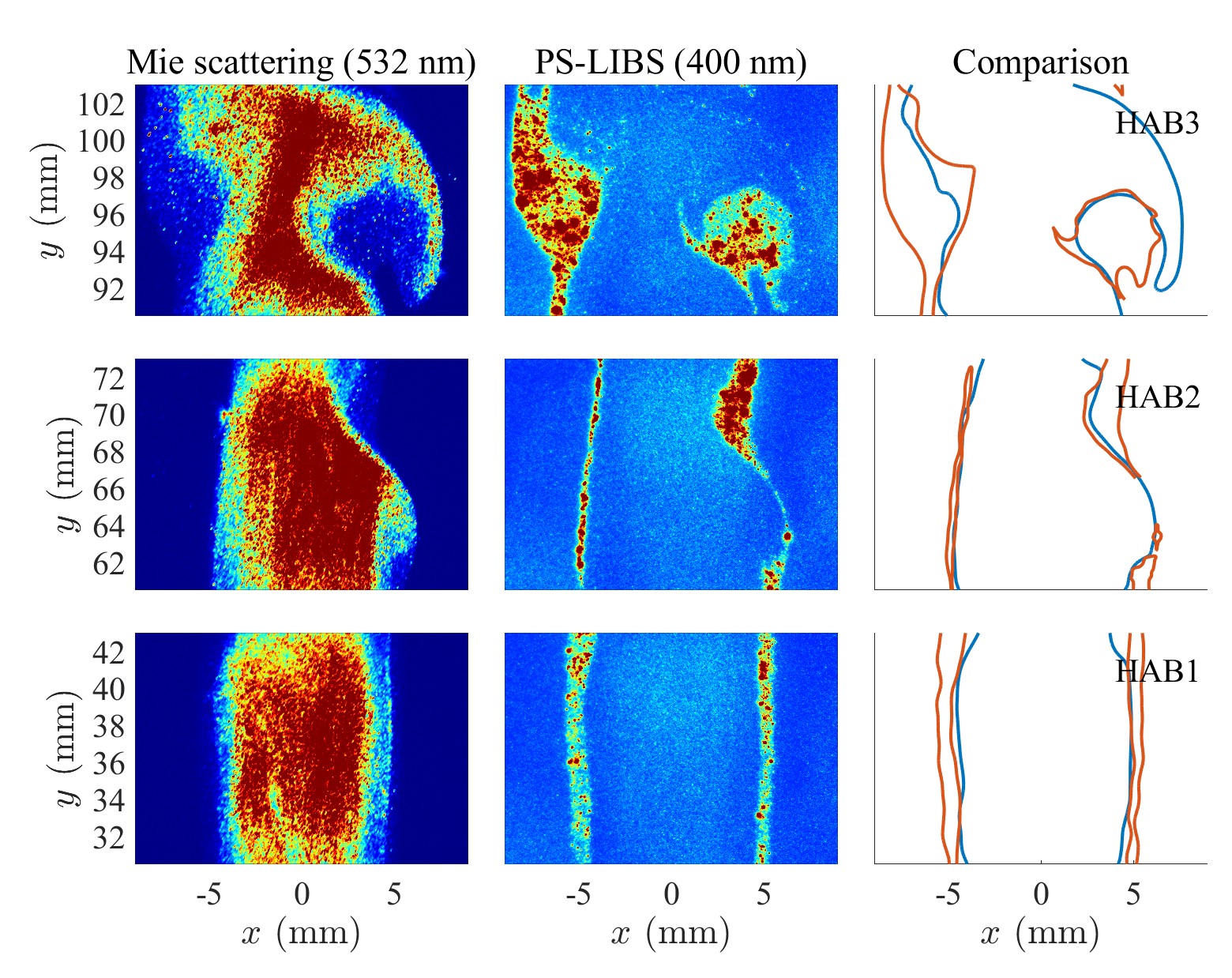

Supplement: Supplementary file 1 — Supplementary material 1 [file 41598_2025_26673_MOESM1_ESM.zip › Fig1.jpg]

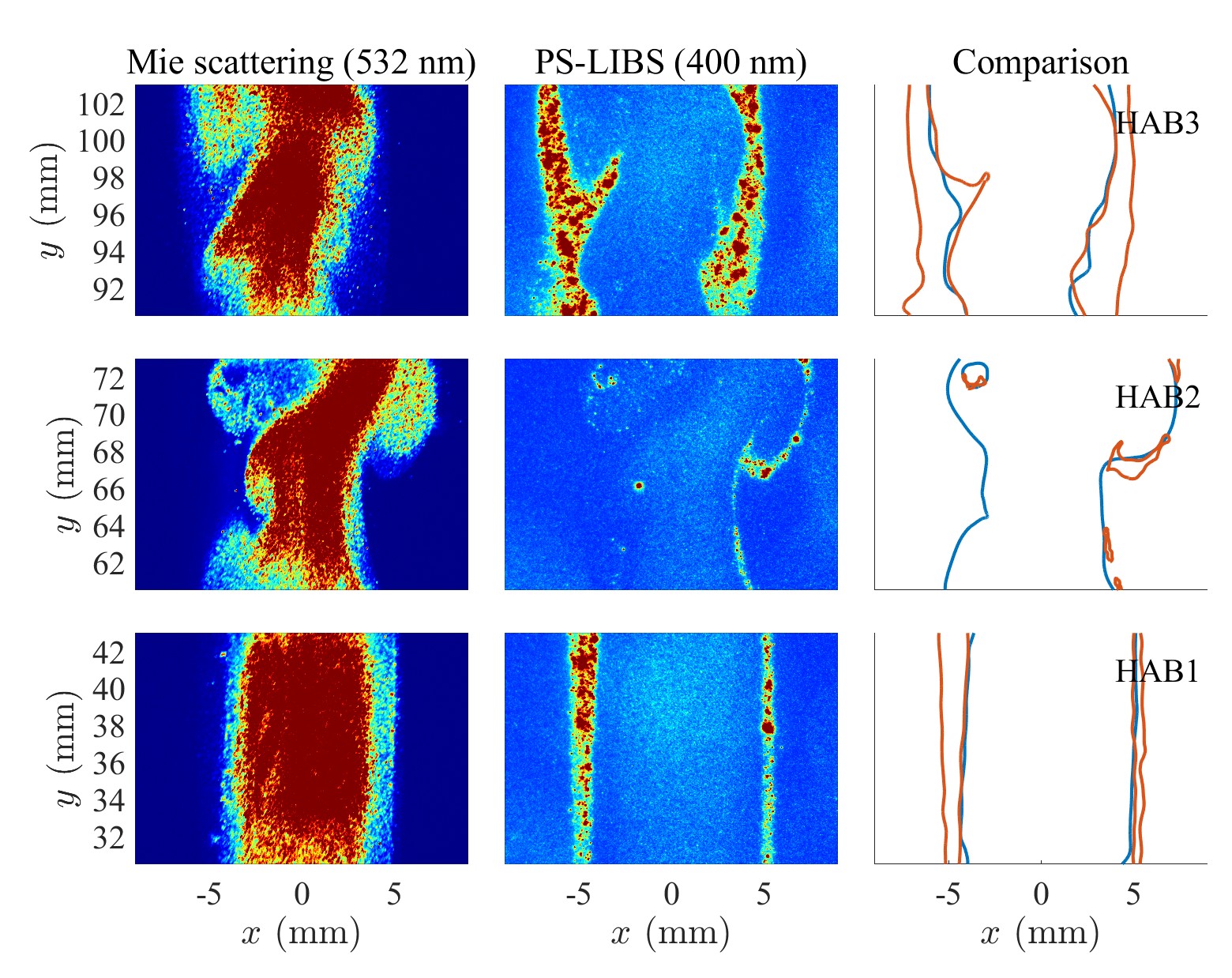

Supplement: Supplementary file 1 — Supplementary material 1 [file 41598_2025_26673_MOESM1_ESM.zip › Fig2.jpg]

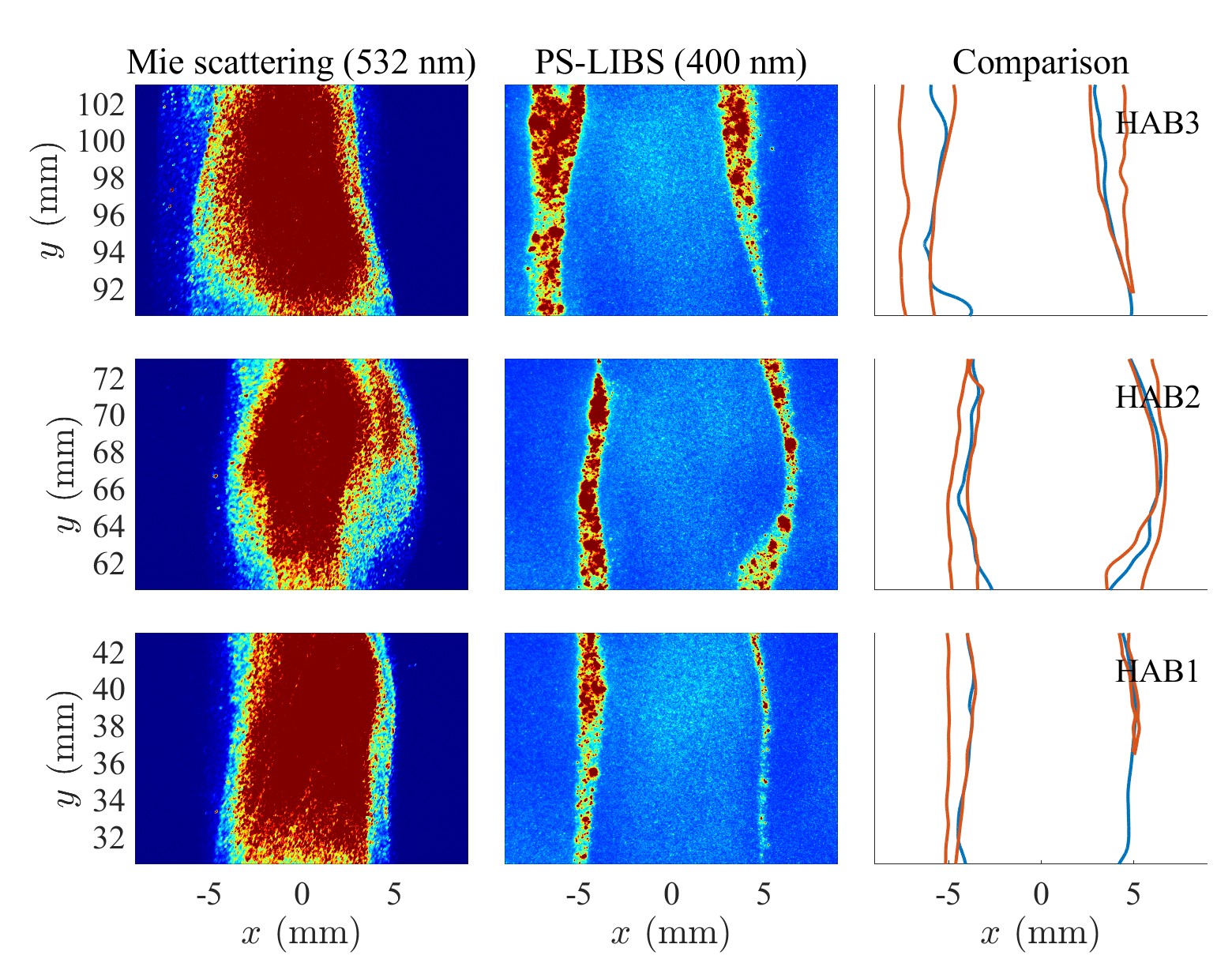

Supplement: Supplementary file 1 — Supplementary material 1 [file 41598_2025_26673_MOESM1_ESM.zip › Fig3.jpg]

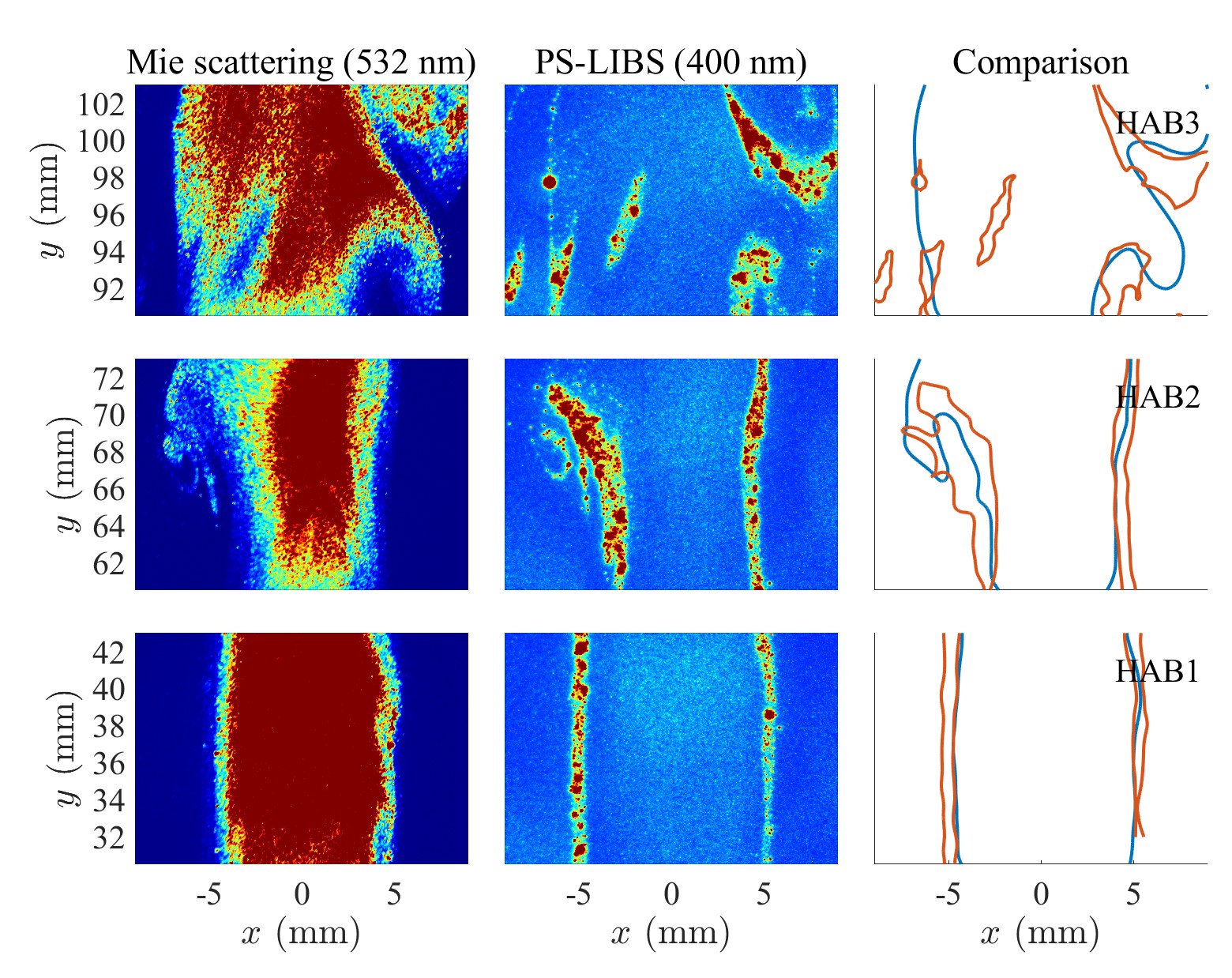

Supplement: Supplementary file 1 — Supplementary material 1 [file 41598_2025_26673_MOESM1_ESM.zip › Fig4.jpg]

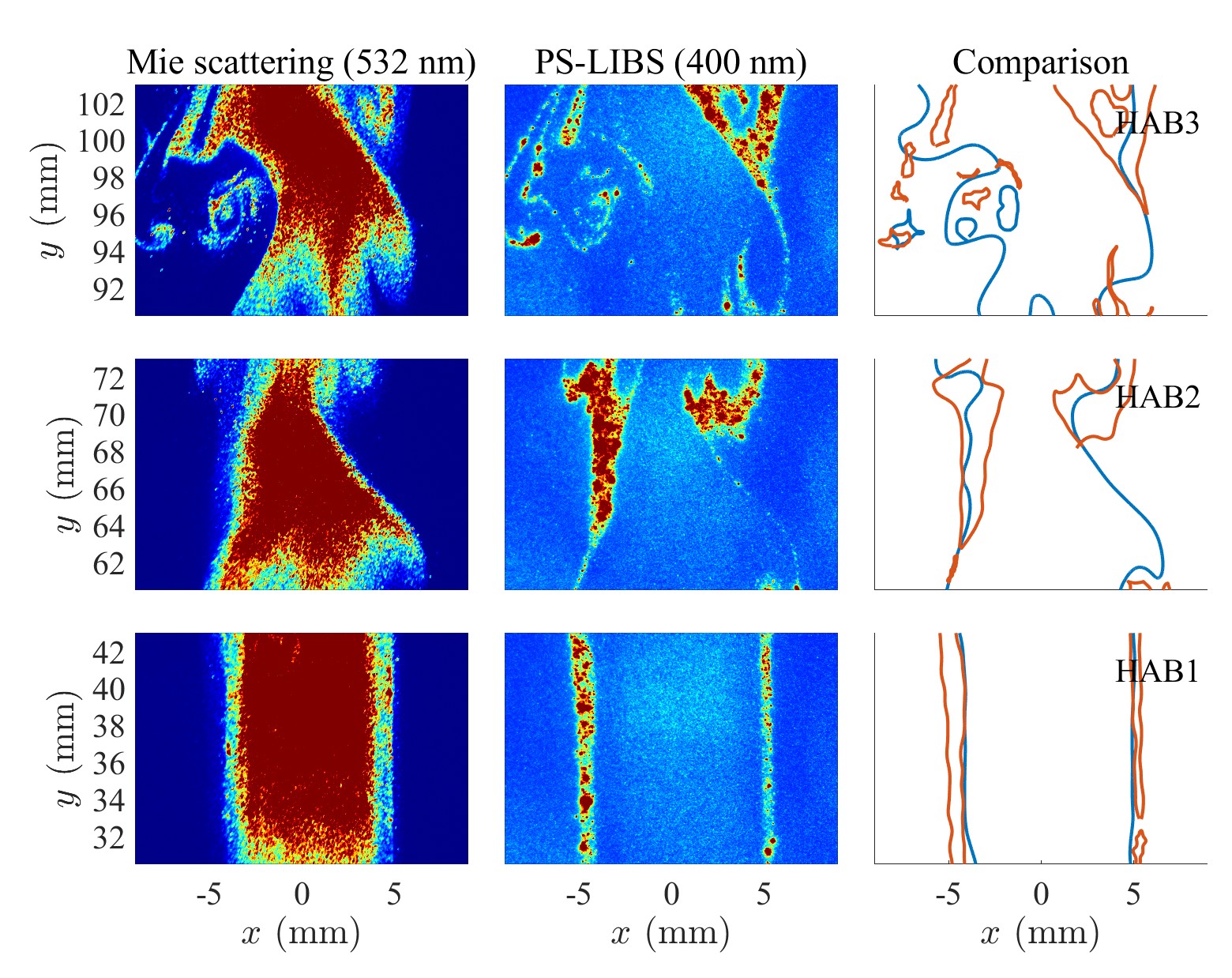

Supplement: Supplementary file 1 — Supplementary material 1 [file 41598_2025_26673_MOESM1_ESM.zip › Fig5.jpg]

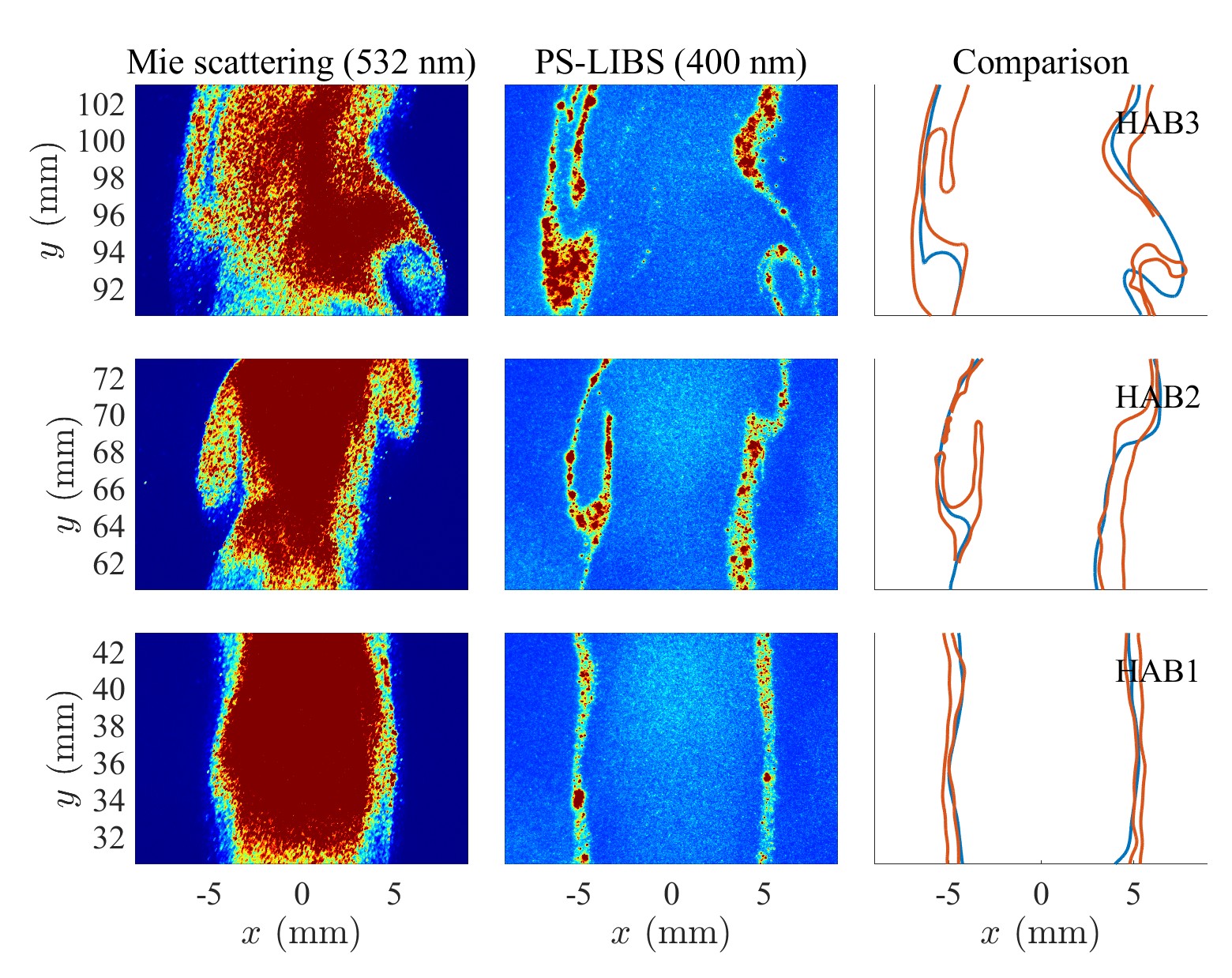

Supplement: Supplementary file 1 — Supplementary material 1 [file 41598_2025_26673_MOESM1_ESM.zip › Fig6.jpg]

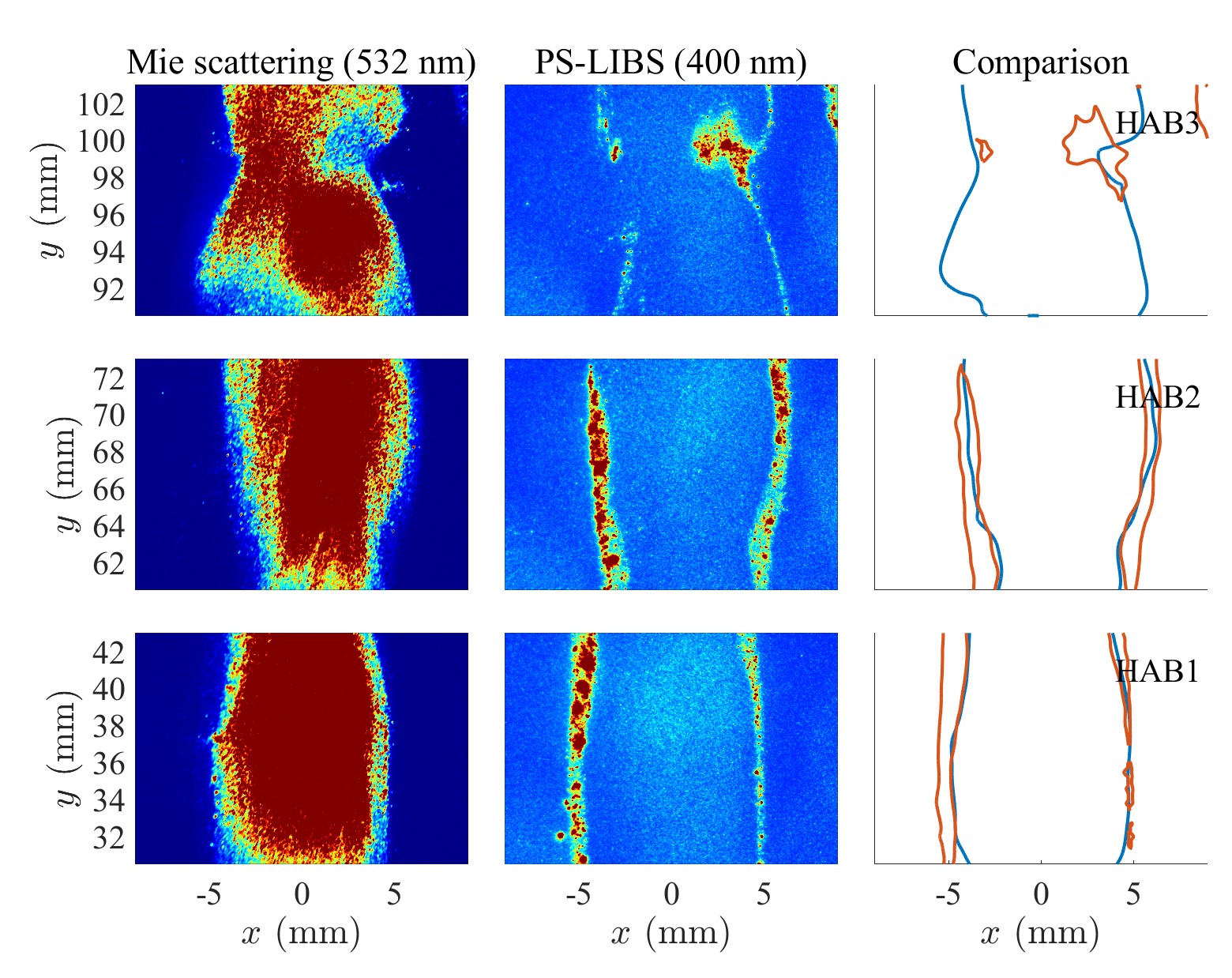

Supplement: Supplementary file 1 — Supplementary material 1 [file 41598_2025_26673_MOESM1_ESM.zip › Fig7.jpg]

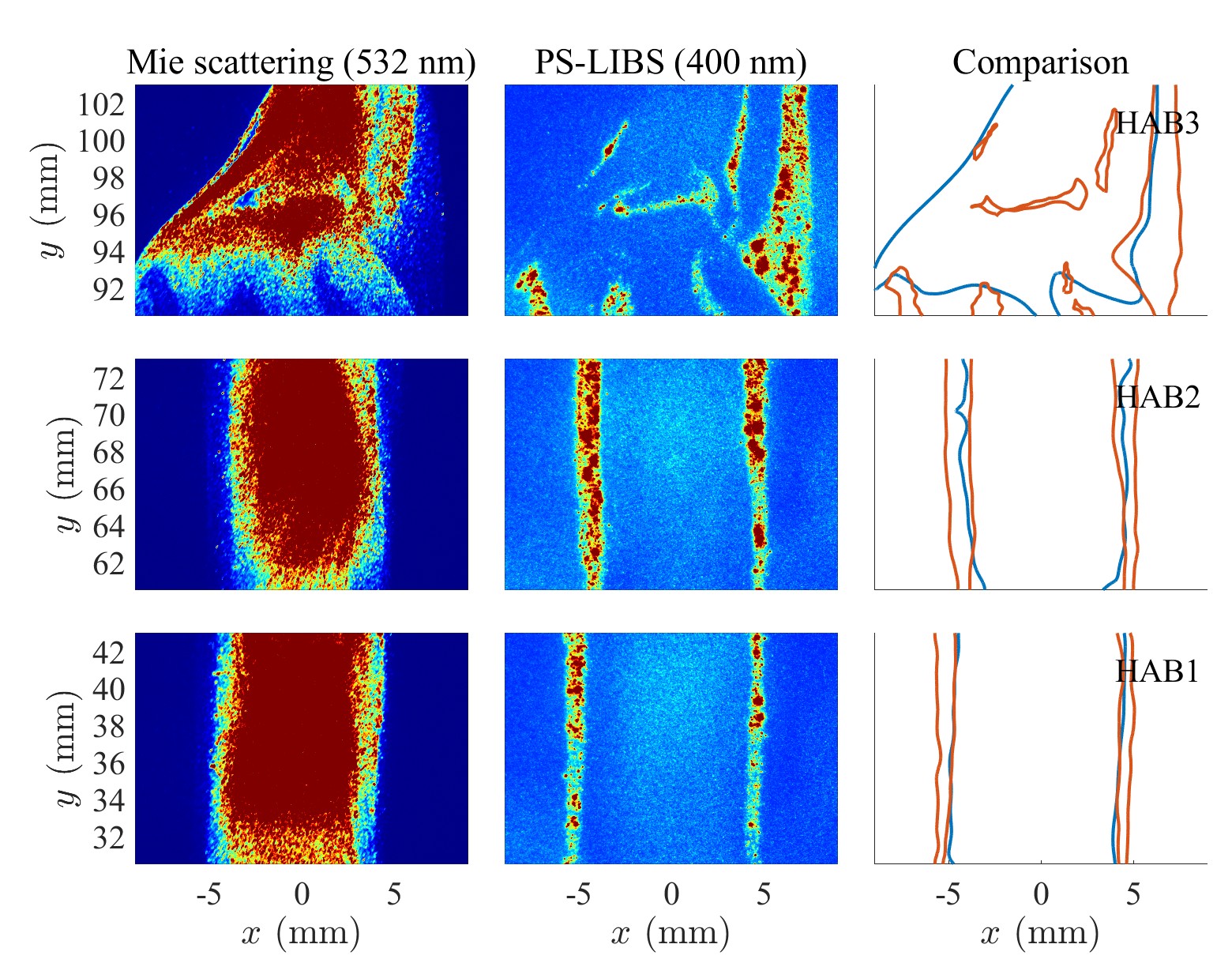

Supplement: Supplementary file 1 — Supplementary material 1 [file 41598_2025_26673_MOESM1_ESM.zip › Fig8.jpg]

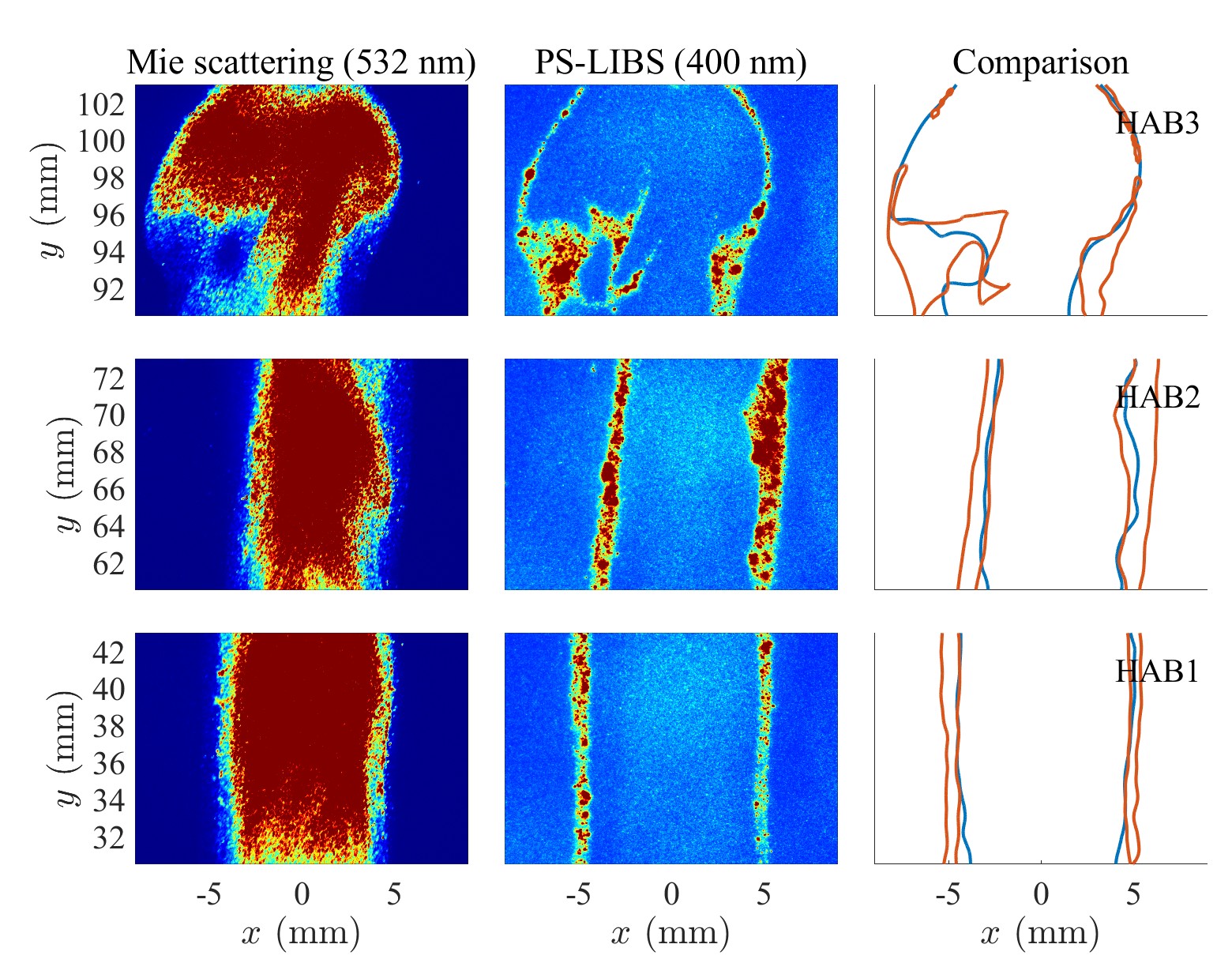

Supplement: Supplementary file 1 — Supplementary material 1 [file 41598_2025_26673_MOESM1_ESM.zip › Fig9.jpg]

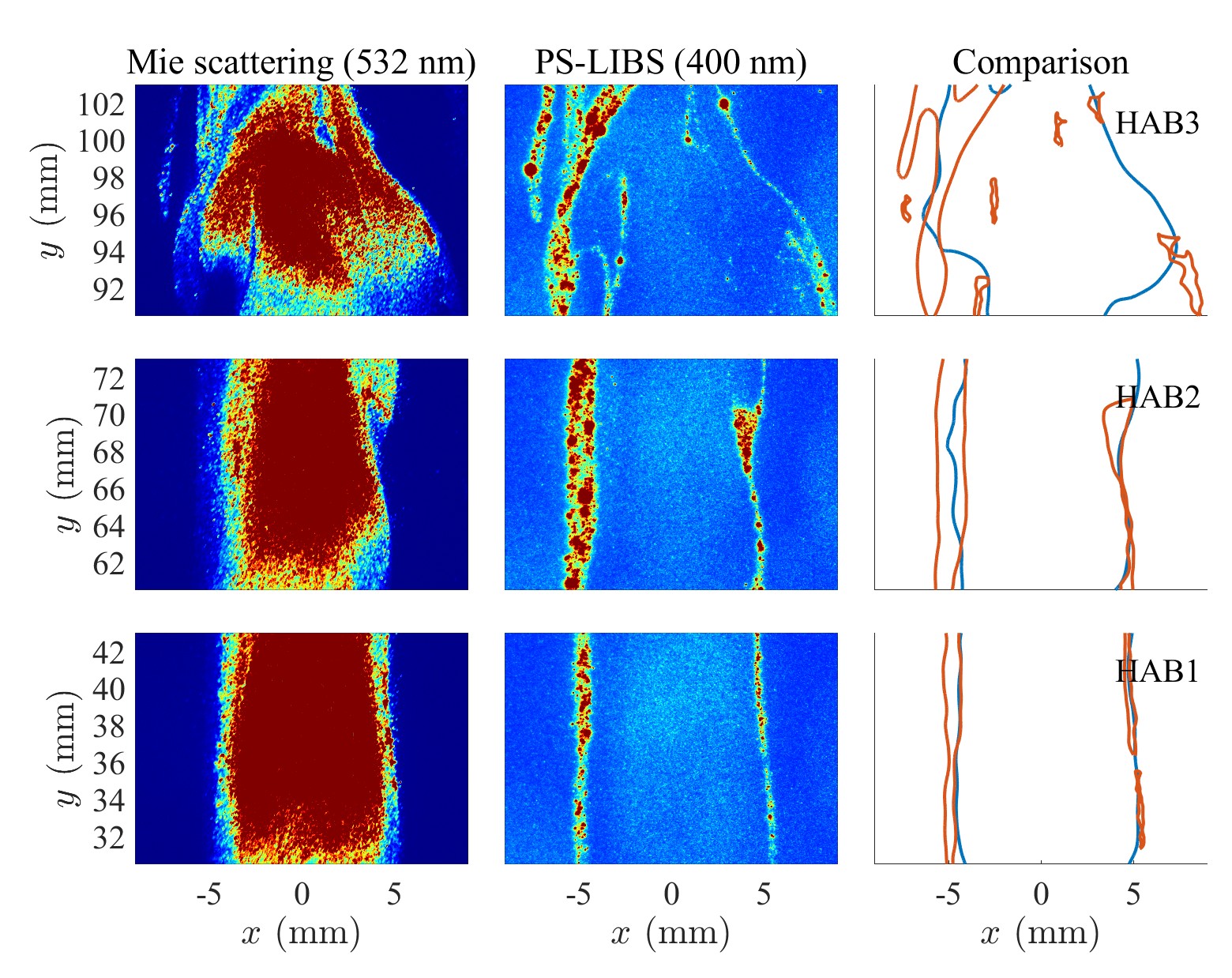

Supplement: Supplementary file 1 — Supplementary material 1 [file 41598_2025_26673_MOESM1_ESM.zip › Fig10.jpg]

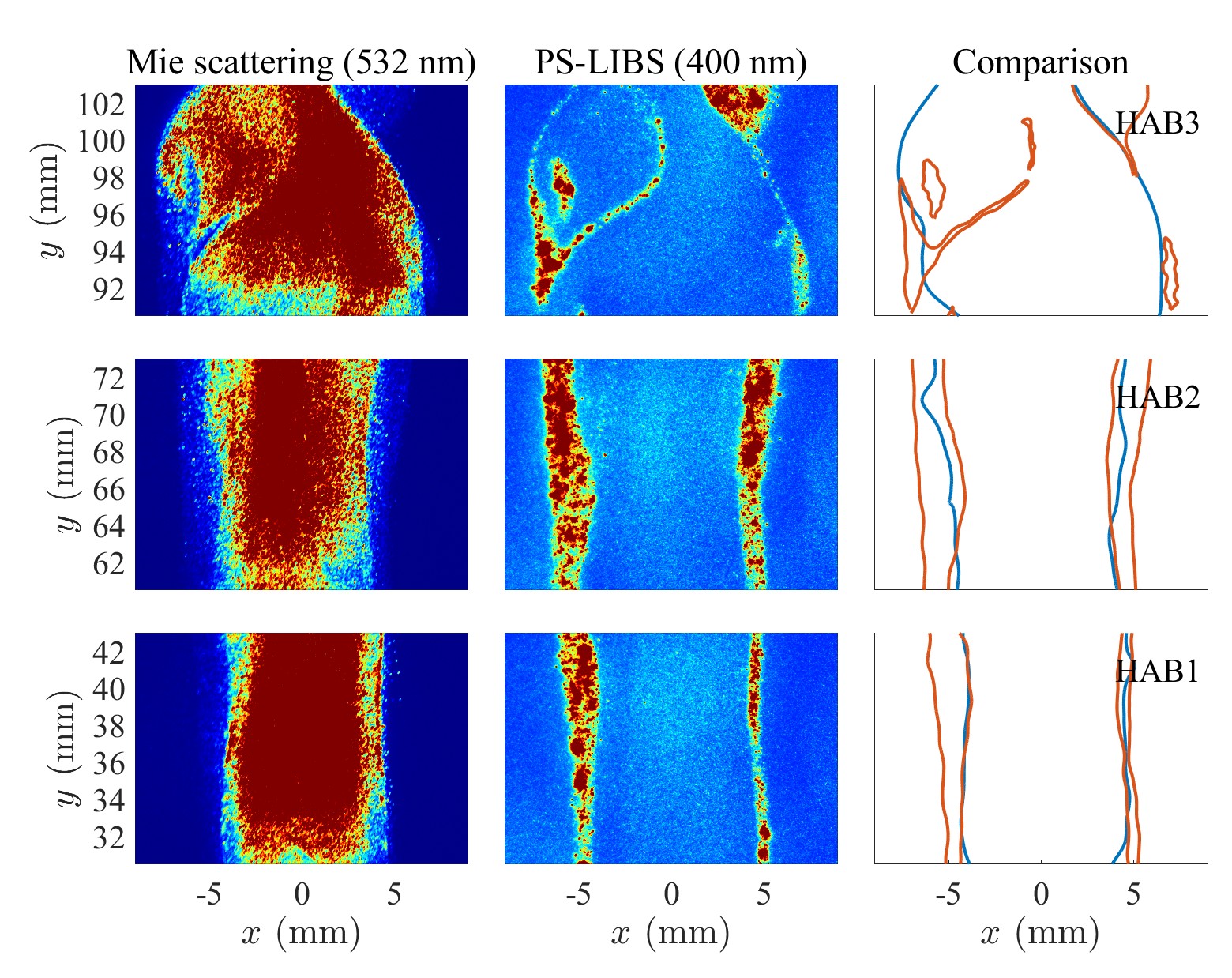

Supplement: Supplementary file 1 — Supplementary material 1 [file 41598_2025_26673_MOESM1_ESM.zip › Fig11.jpg]

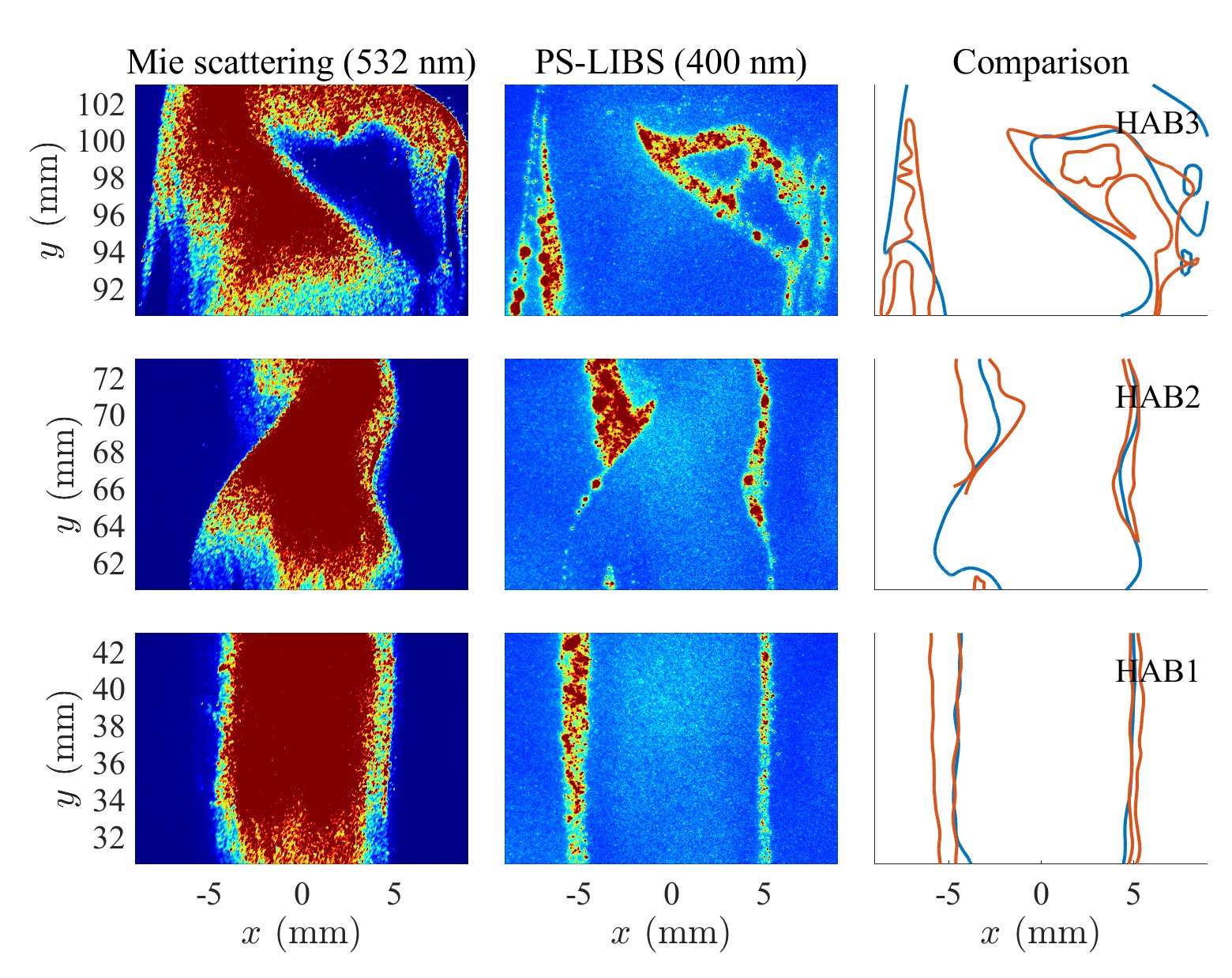

Supplement: Supplementary file 1 — Supplementary material 1 [file 41598_2025_26673_MOESM1_ESM.zip › Fig12.jpg]

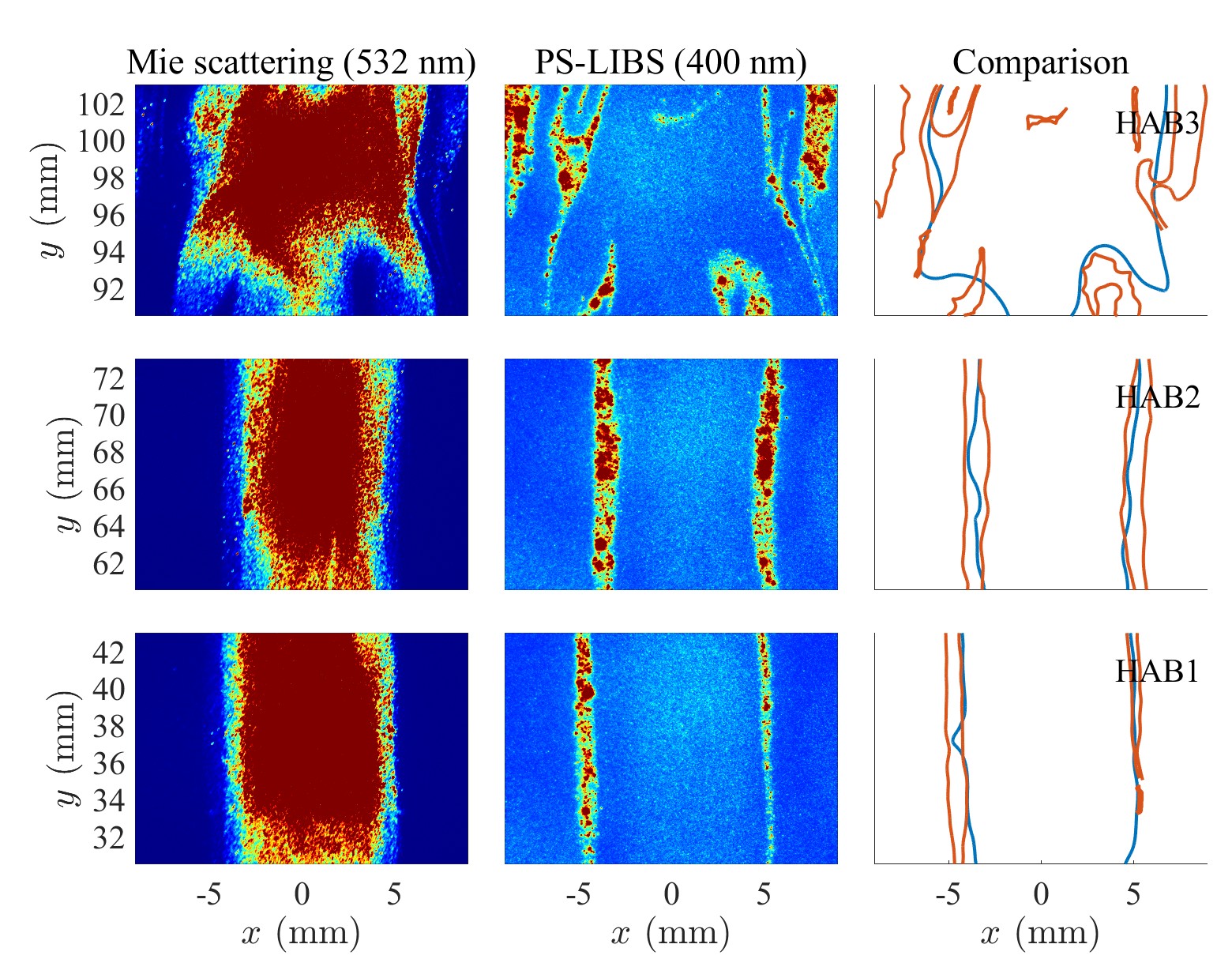

Supplement: Supplementary file 1 — Supplementary material 1 [file 41598_2025_26673_MOESM1_ESM.zip › Fig13.jpg]

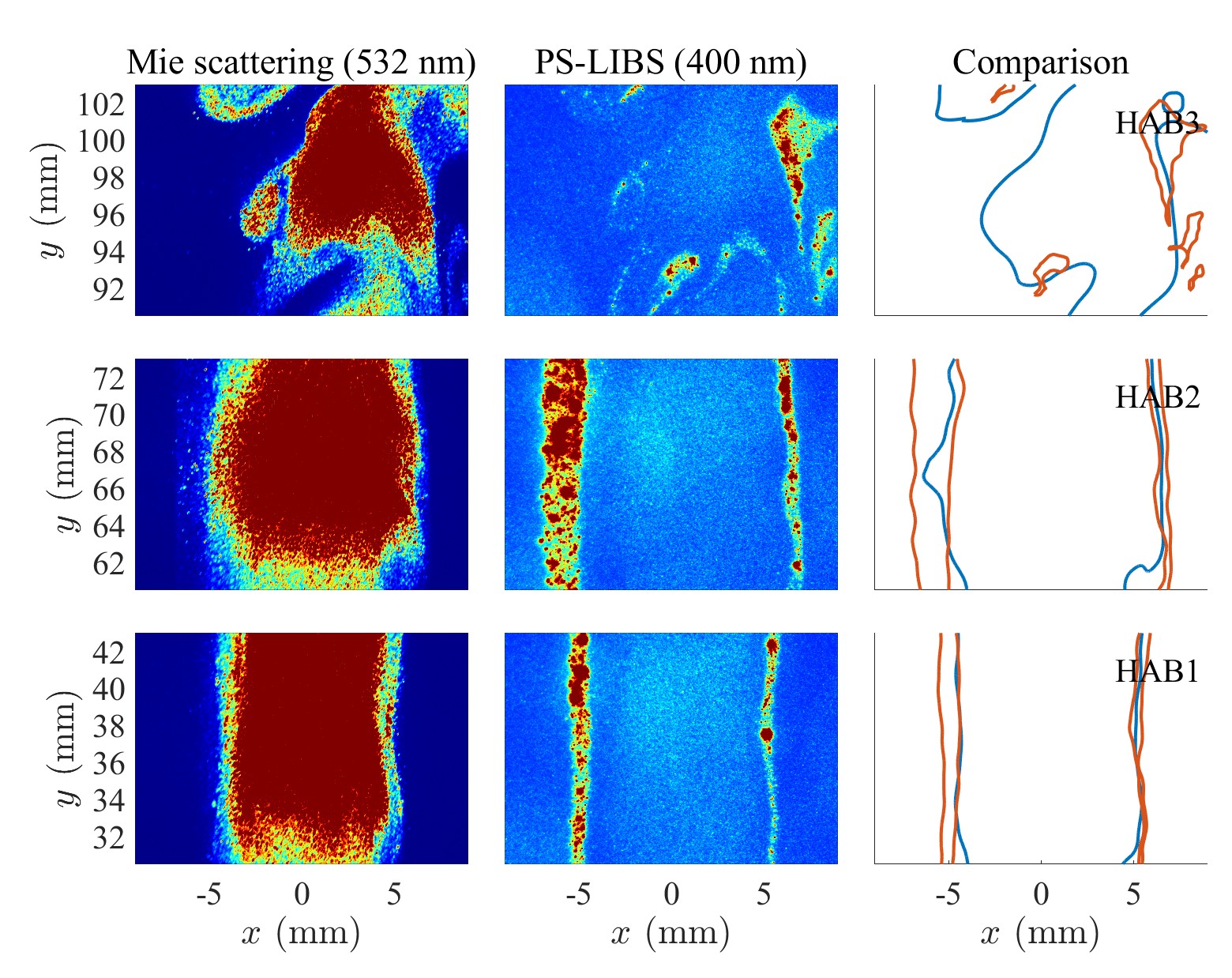

Supplement: Supplementary file 1 — Supplementary material 1 [file 41598_2025_26673_MOESM1_ESM.zip › Fig14.jpg]

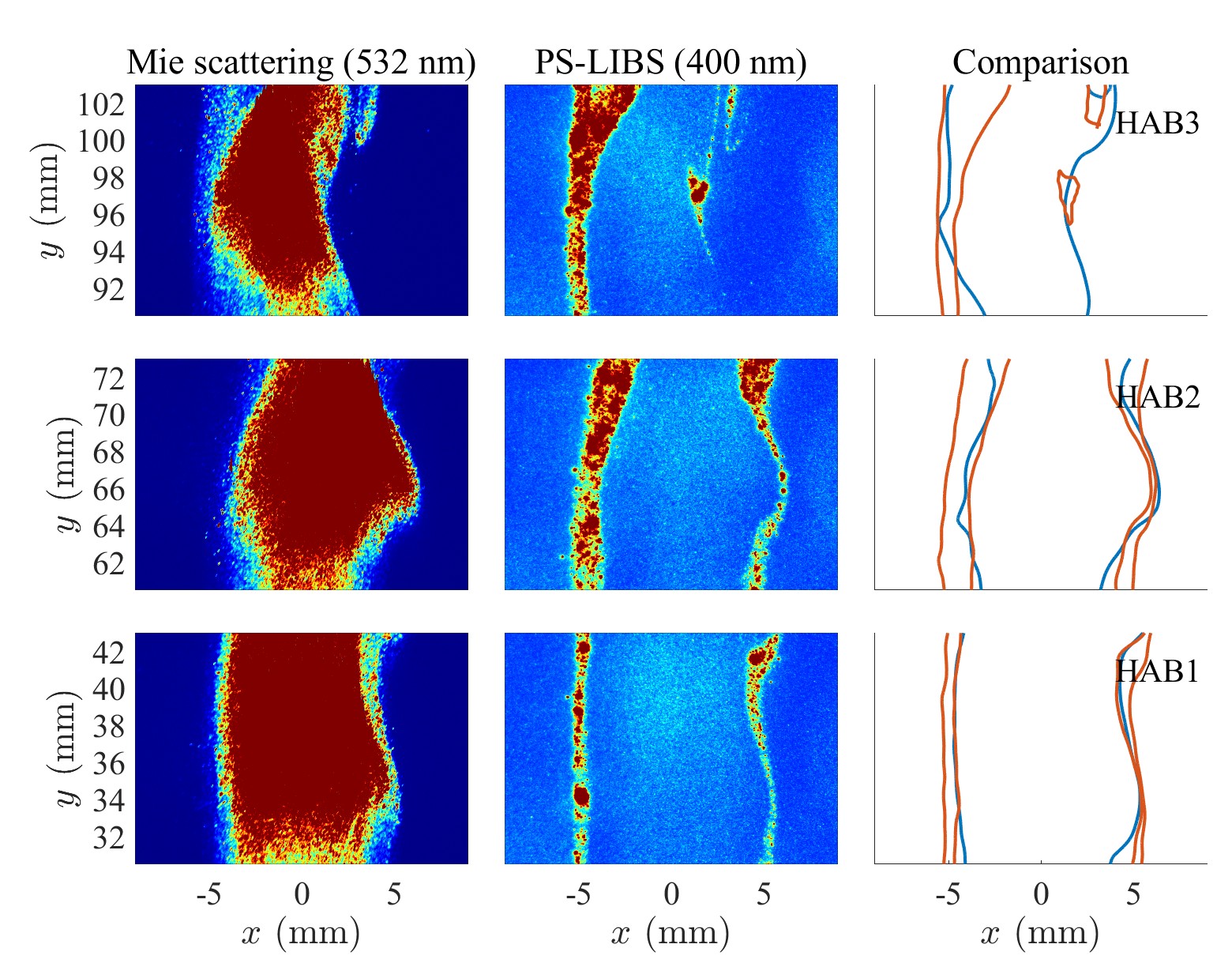

Supplement: Supplementary file 1 — Supplementary material 1 [file 41598_2025_26673_MOESM1_ESM.zip › Fig15.jpg]

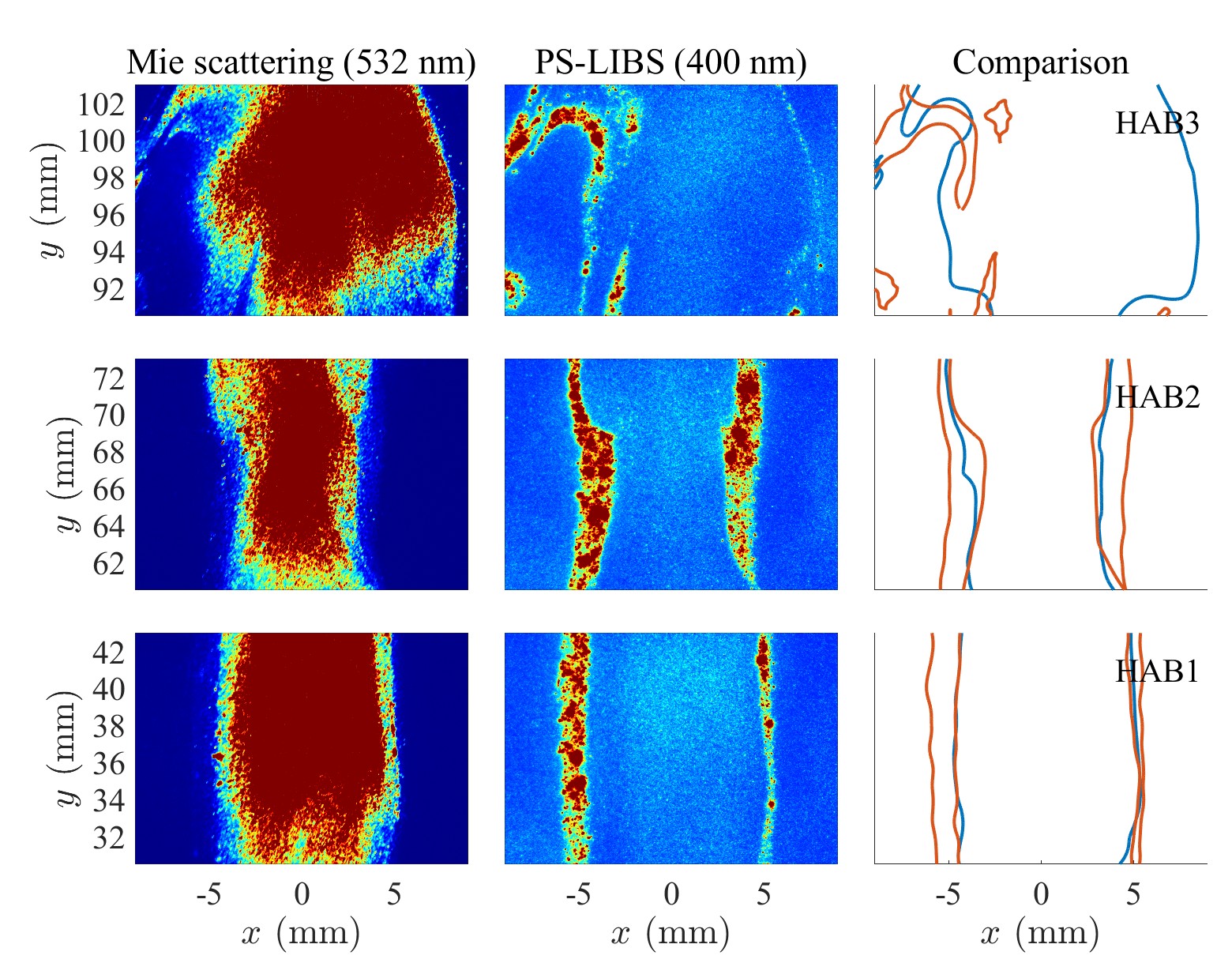

Supplement: Supplementary file 1 — Supplementary material 1 [file 41598_2025_26673_MOESM1_ESM.zip › Fig16.jpg]

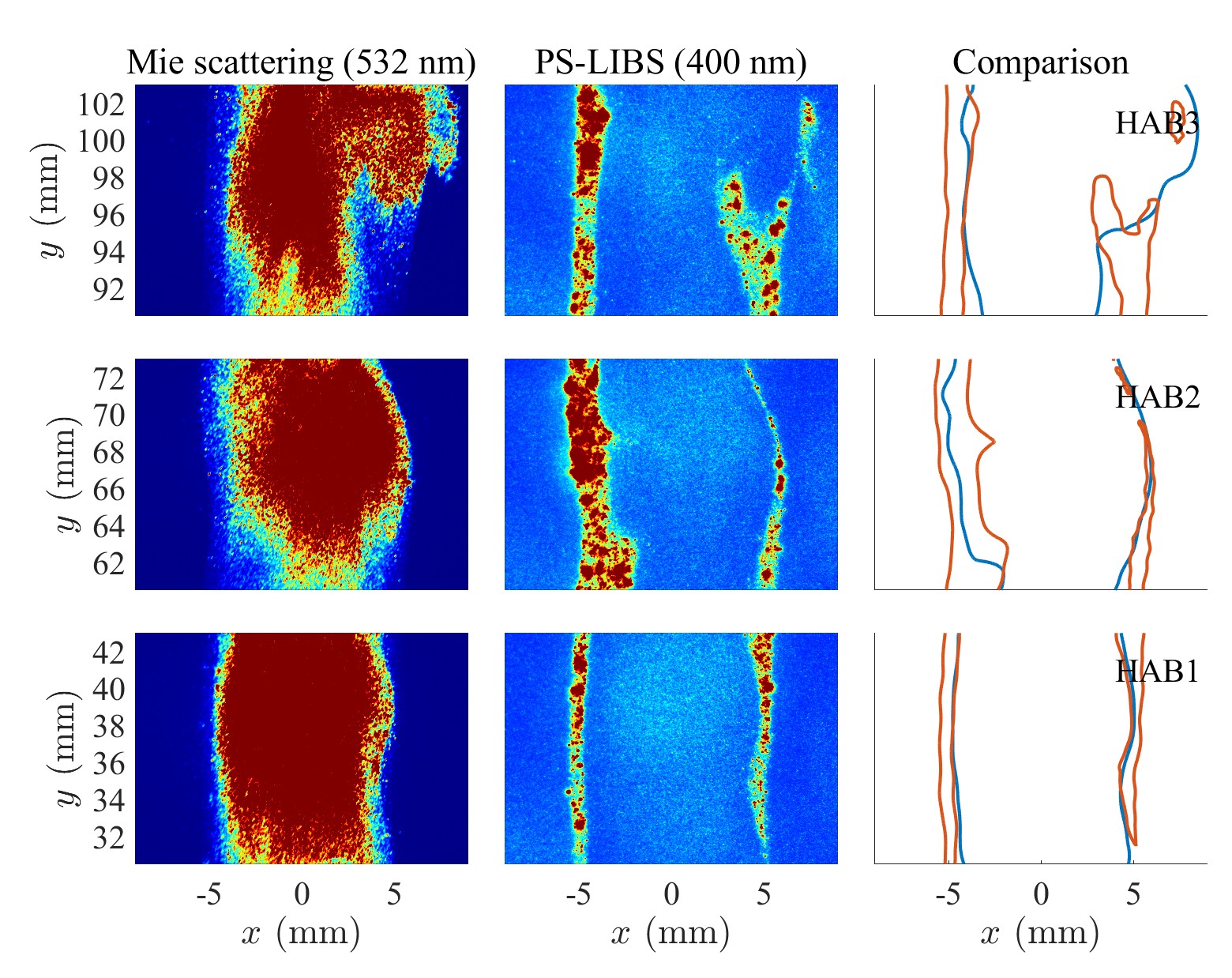

Supplement: Supplementary file 1 — Supplementary material 1 [file 41598_2025_26673_MOESM1_ESM.zip › Fig17.jpg]

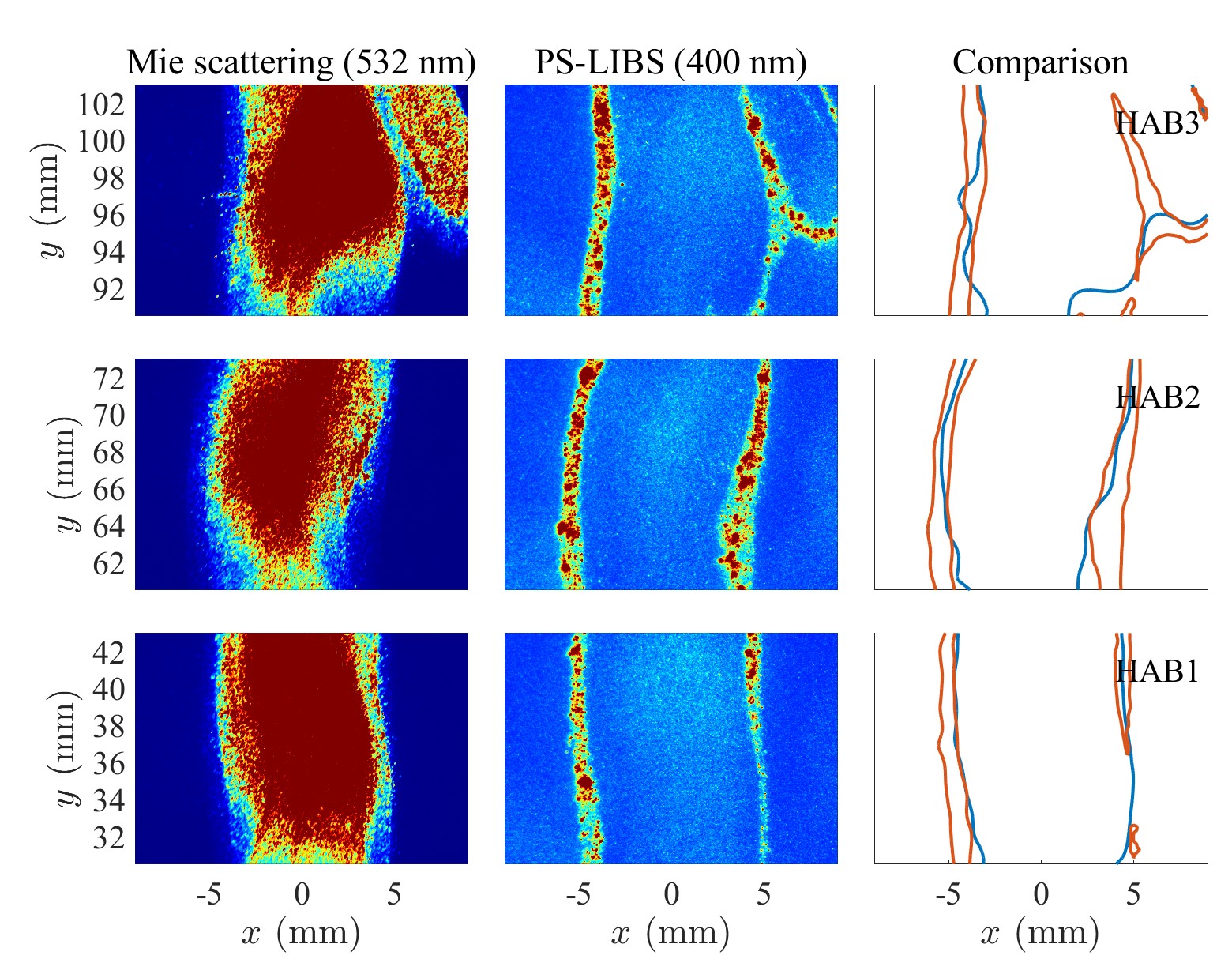

Supplement: Supplementary file 1 — Supplementary material 1 [file 41598_2025_26673_MOESM1_ESM.zip › Fig18.jpg]

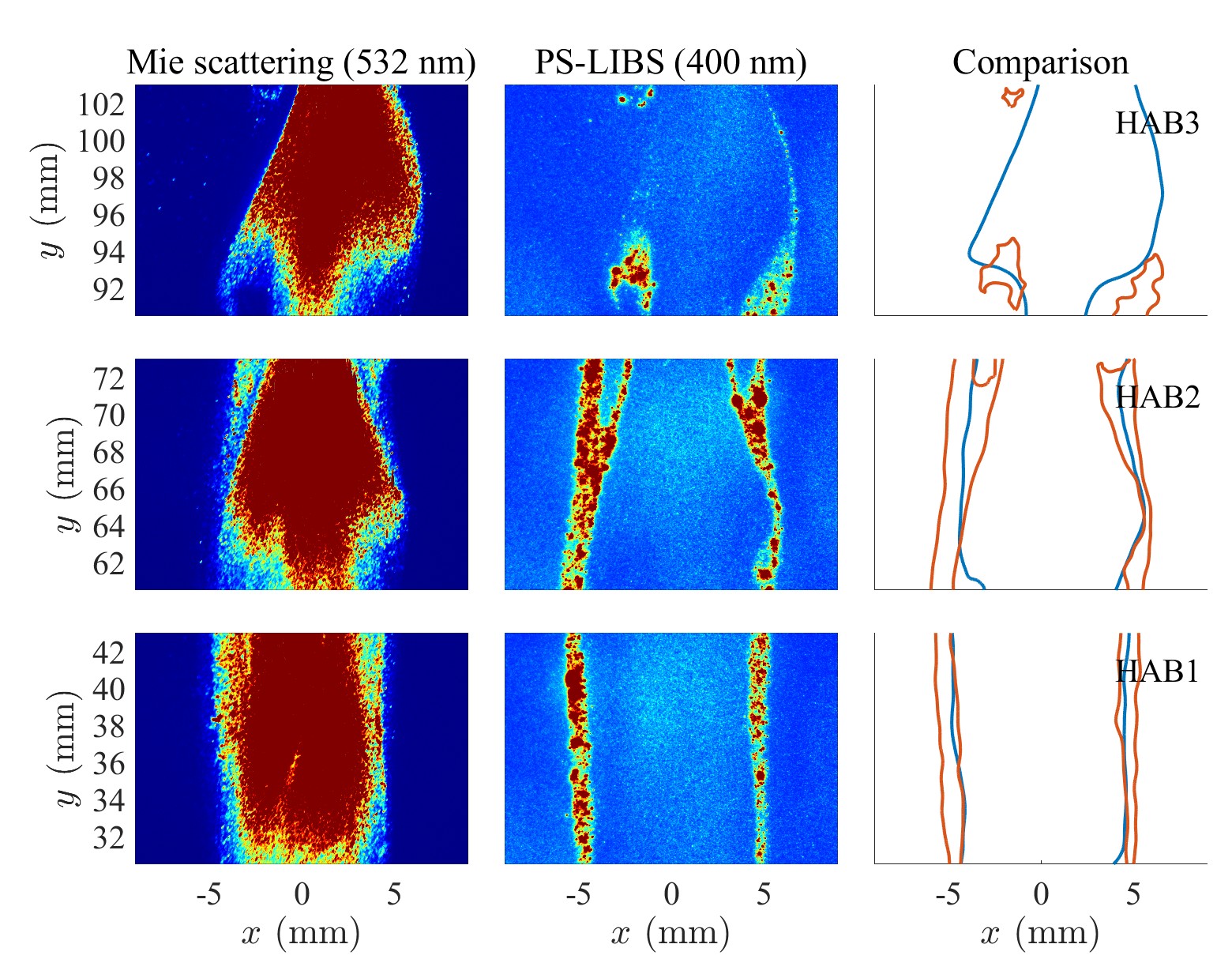

Supplement: Supplementary file 1 — Supplementary material 1 [file 41598_2025_26673_MOESM1_ESM.zip › Fig19.jpg]

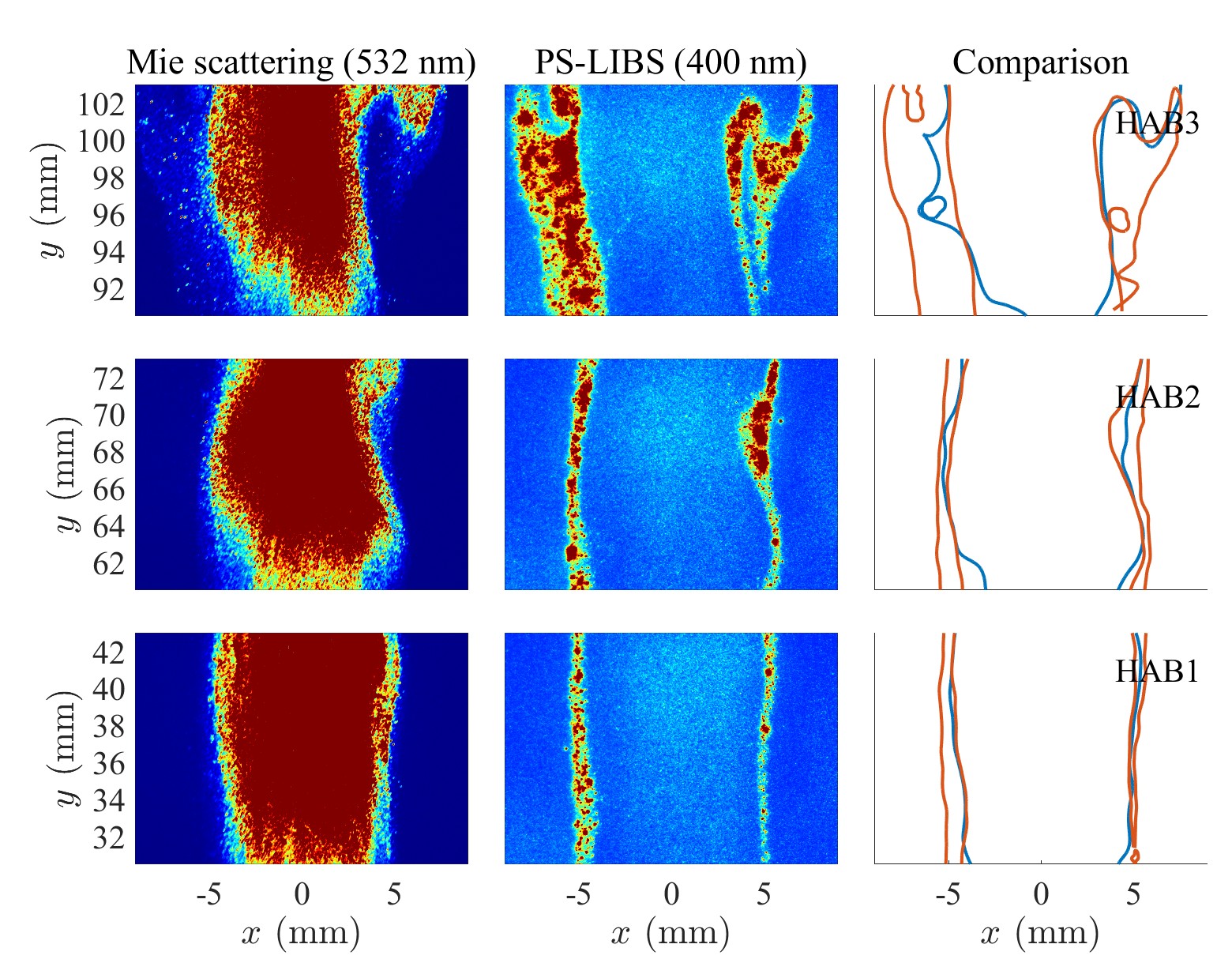

Supplement: Supplementary file 1 — Supplementary material 1 [file 41598_2025_26673_MOESM1_ESM.zip › Fig20.jpg]

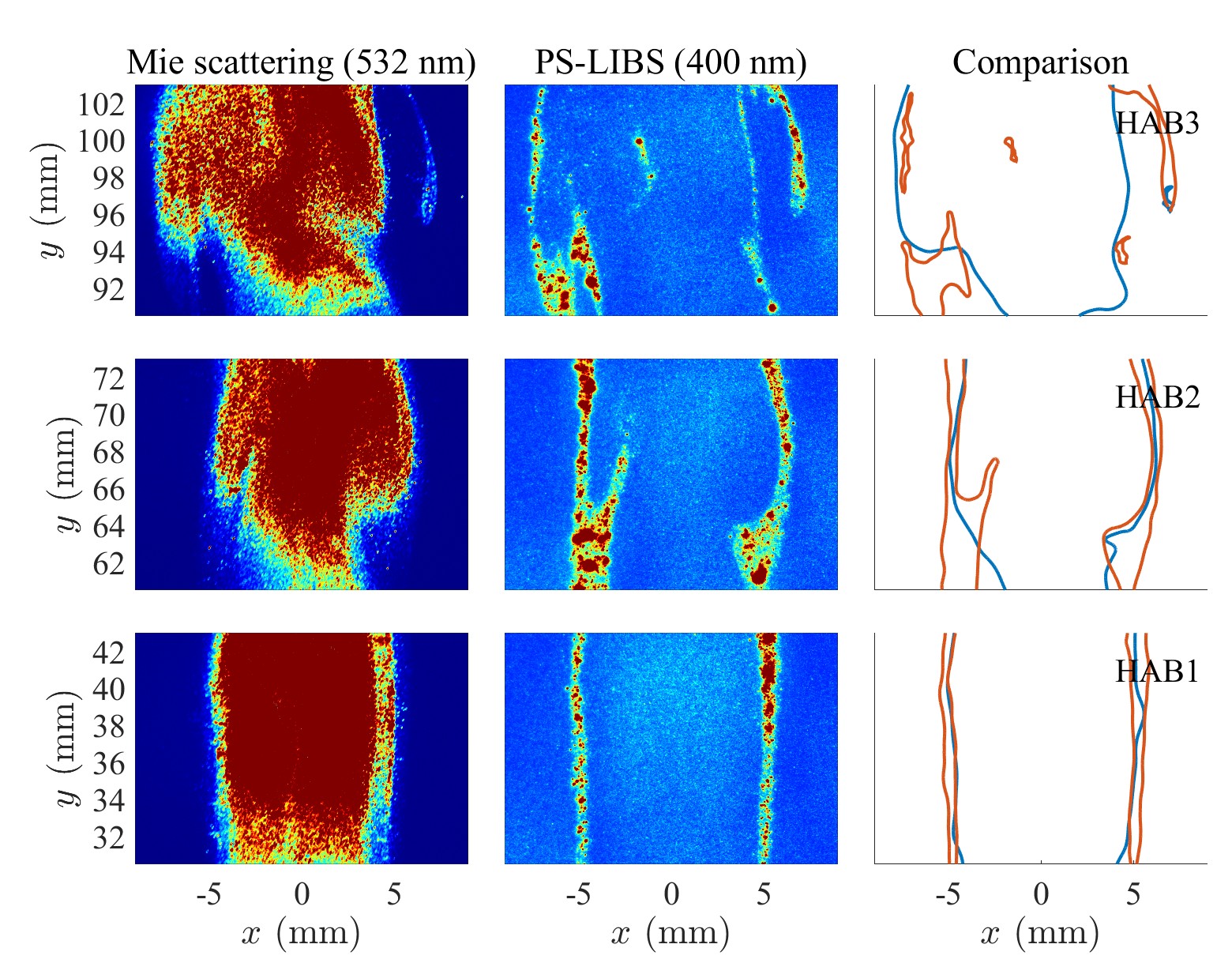

Supplement: Supplementary file 1 — Supplementary material 1 [file 41598_2025_26673_MOESM1_ESM.zip › Fig21.jpg]

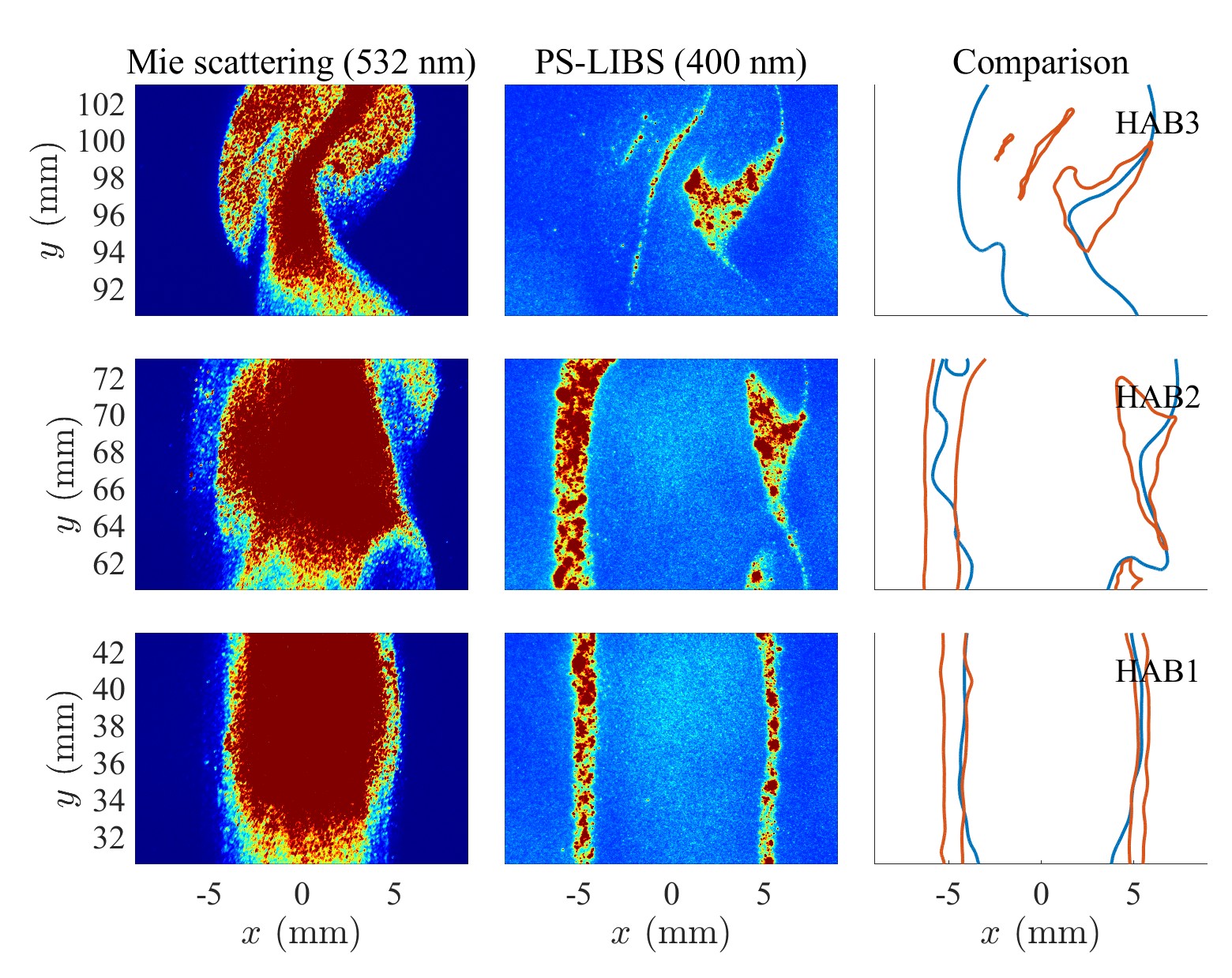

Supplement: Supplementary file 1 — Supplementary material 1 [file 41598_2025_26673_MOESM1_ESM.zip › Fig22.jpg]

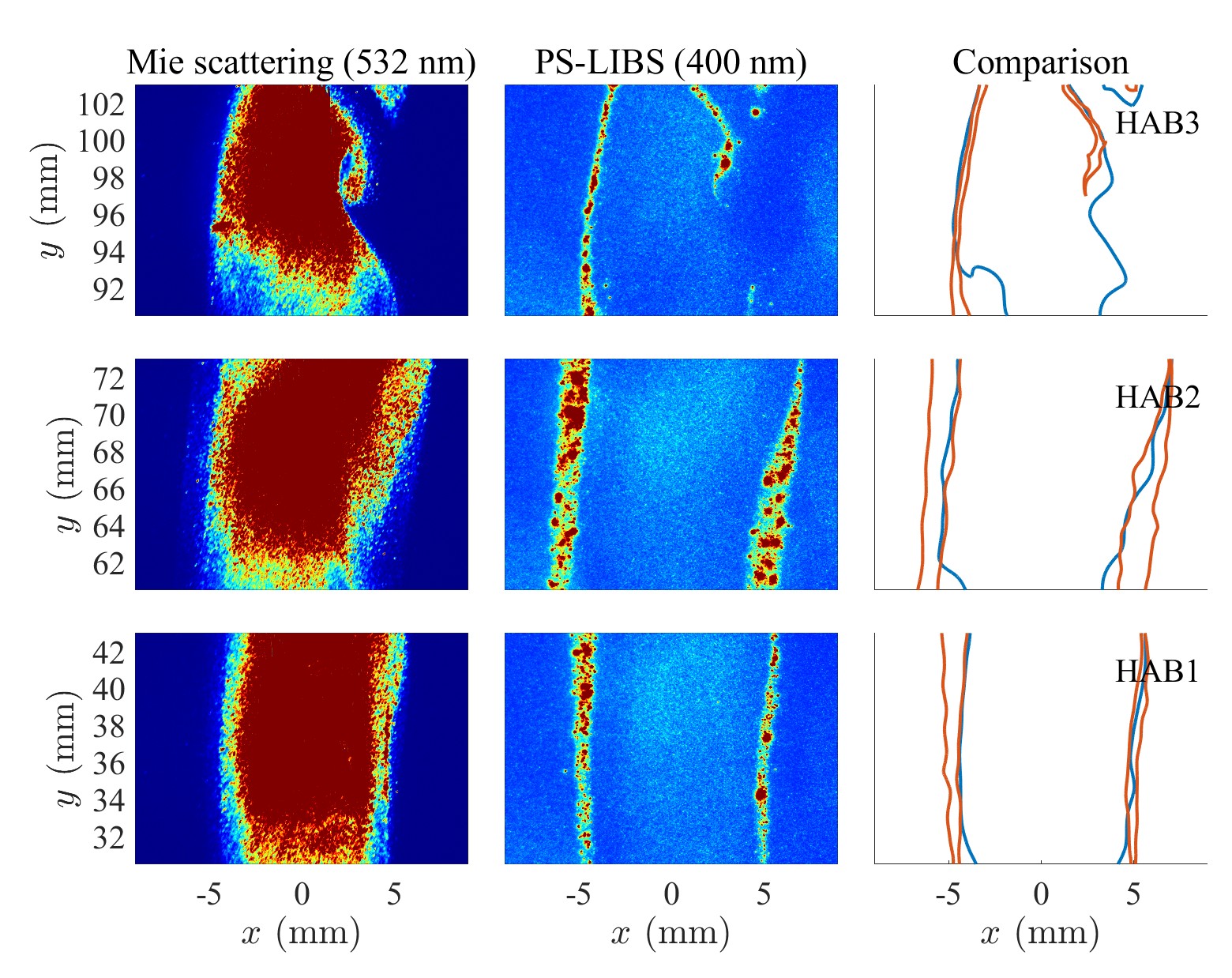

Supplement: Supplementary file 1 — Supplementary material 1 [file 41598_2025_26673_MOESM1_ESM.zip › Fig23.jpg]

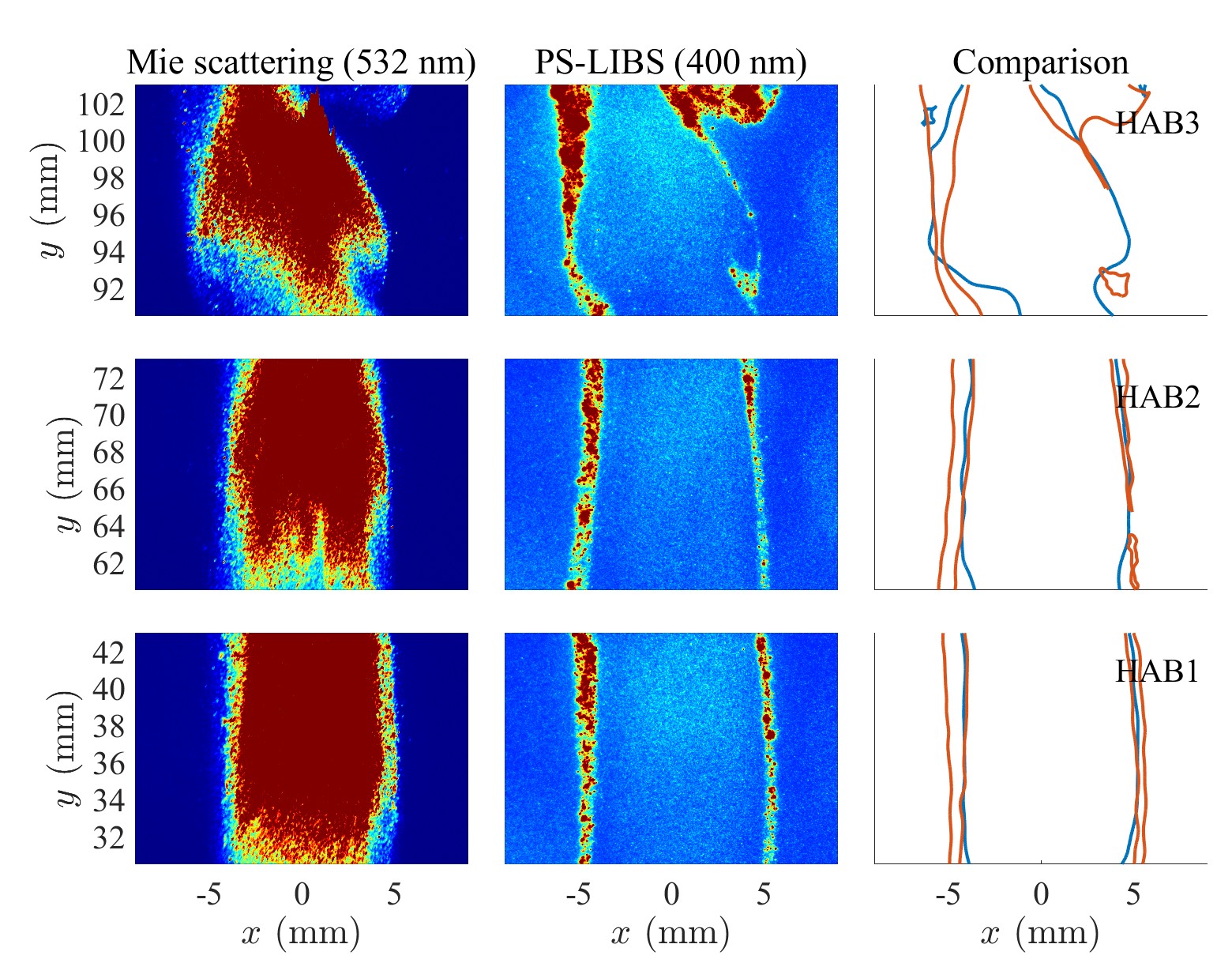

Supplement: Supplementary file 1 — Supplementary material 1 [file 41598_2025_26673_MOESM1_ESM.zip › Fig24.jpg]

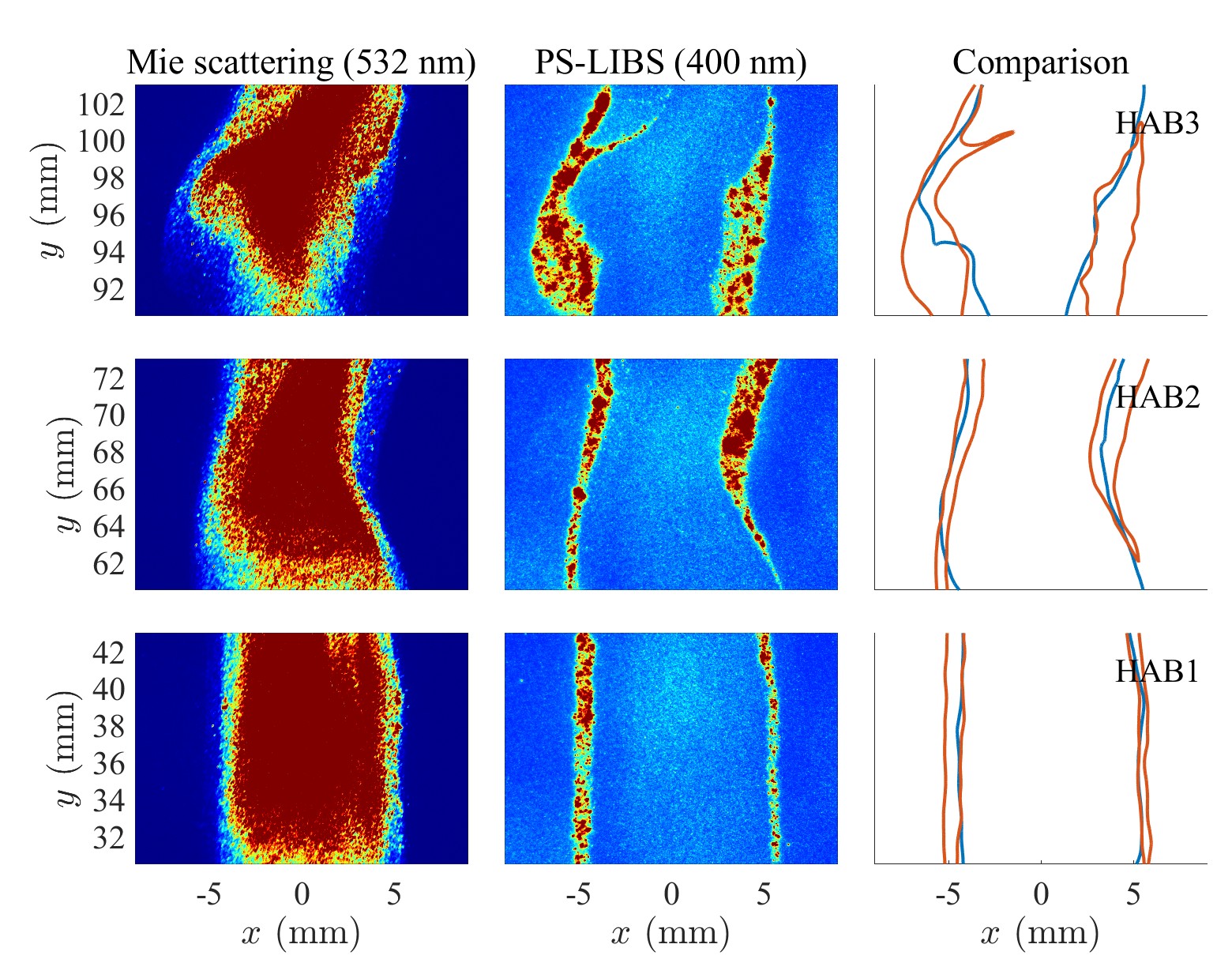

Supplement: Supplementary file 1 — Supplementary material 1 [file 41598_2025_26673_MOESM1_ESM.zip › Fig25.jpg]

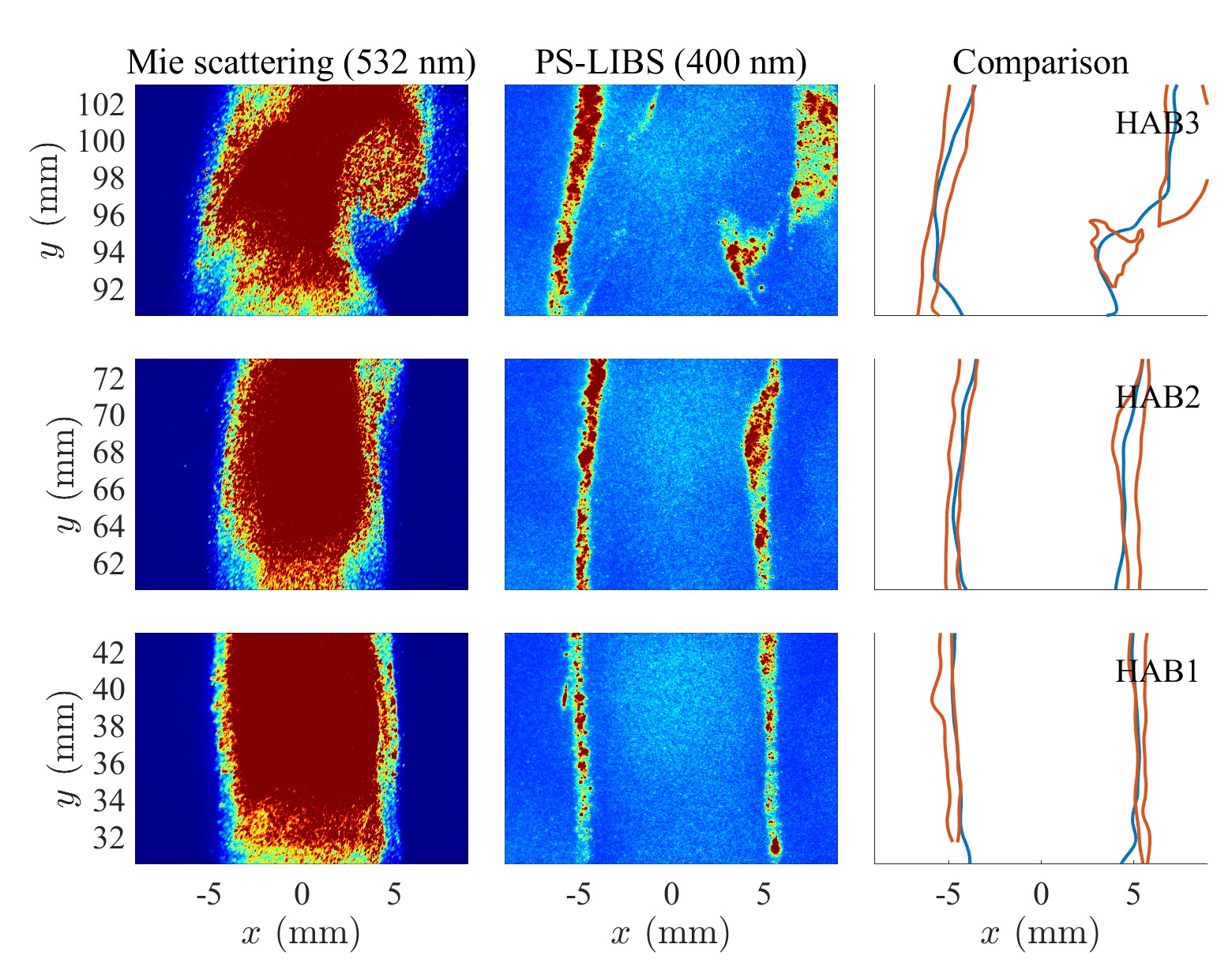

Supplement: Supplementary file 1 — Supplementary material 1 [file 41598_2025_26673_MOESM1_ESM.zip › Fig26.jpg]

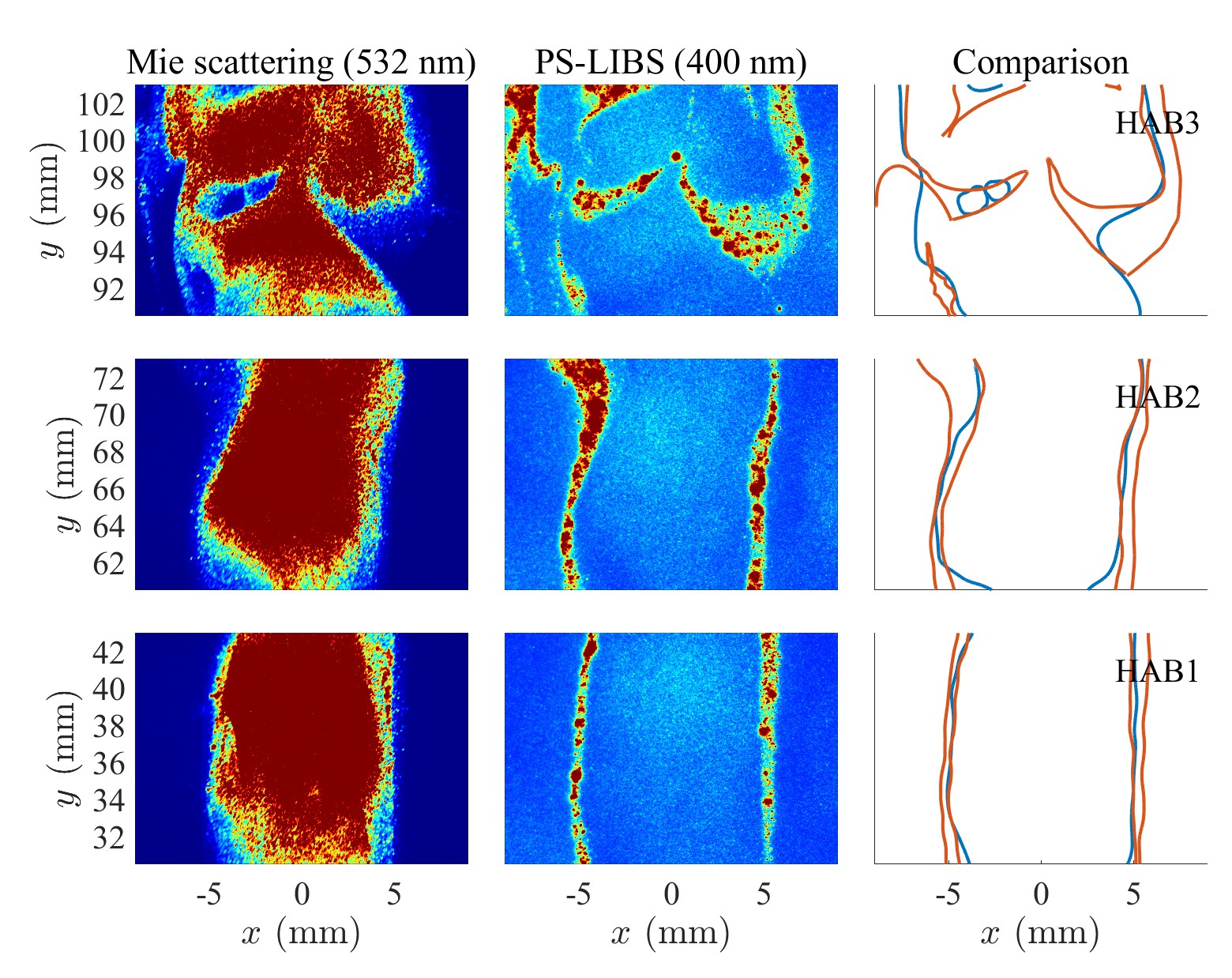

Supplement: Supplementary file 1 — Supplementary material 1 [file 41598_2025_26673_MOESM1_ESM.zip › Fig27.jpg]

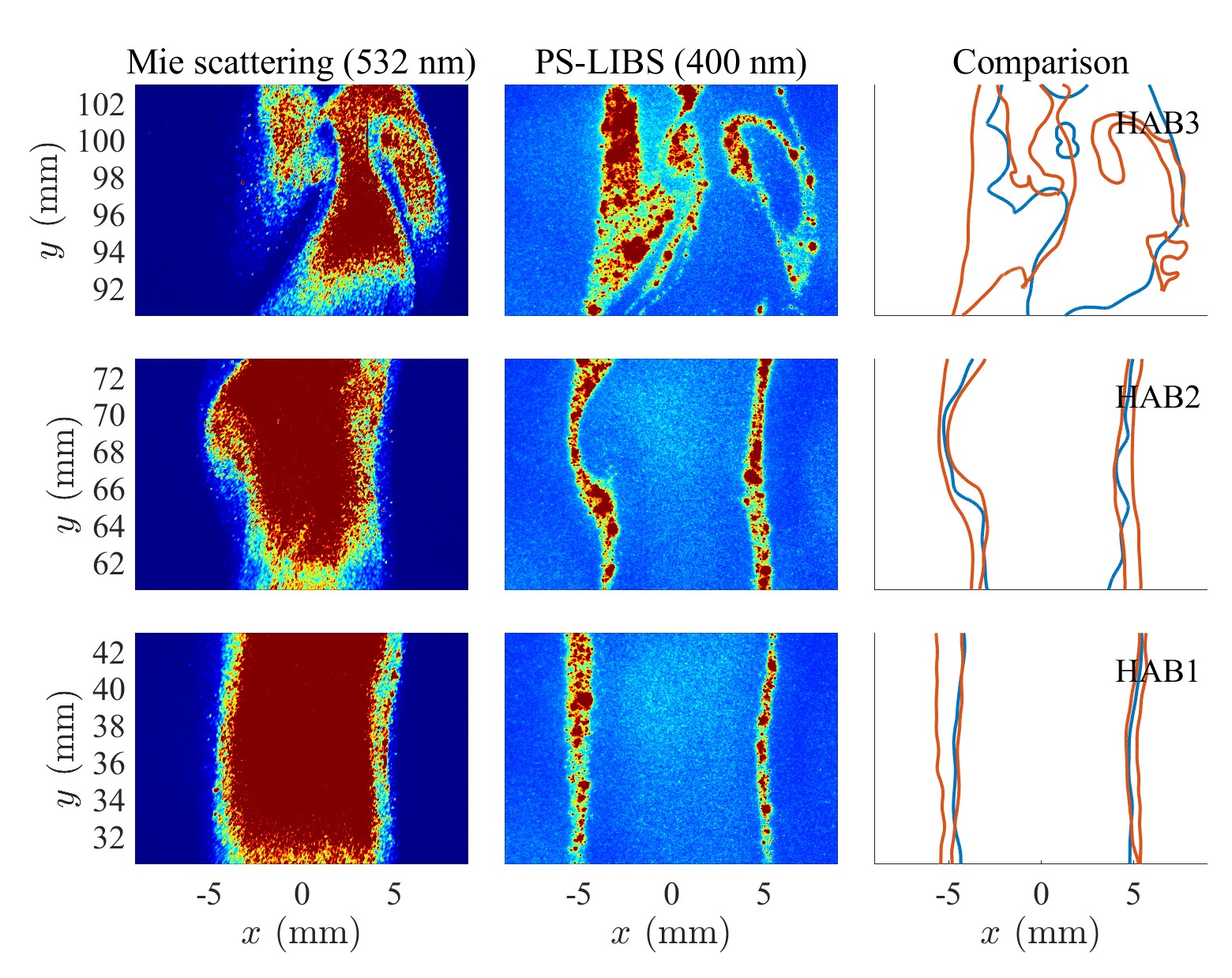

Supplement: Supplementary file 1 — Supplementary material 1 [file 41598_2025_26673_MOESM1_ESM.zip › Fig28.jpg]

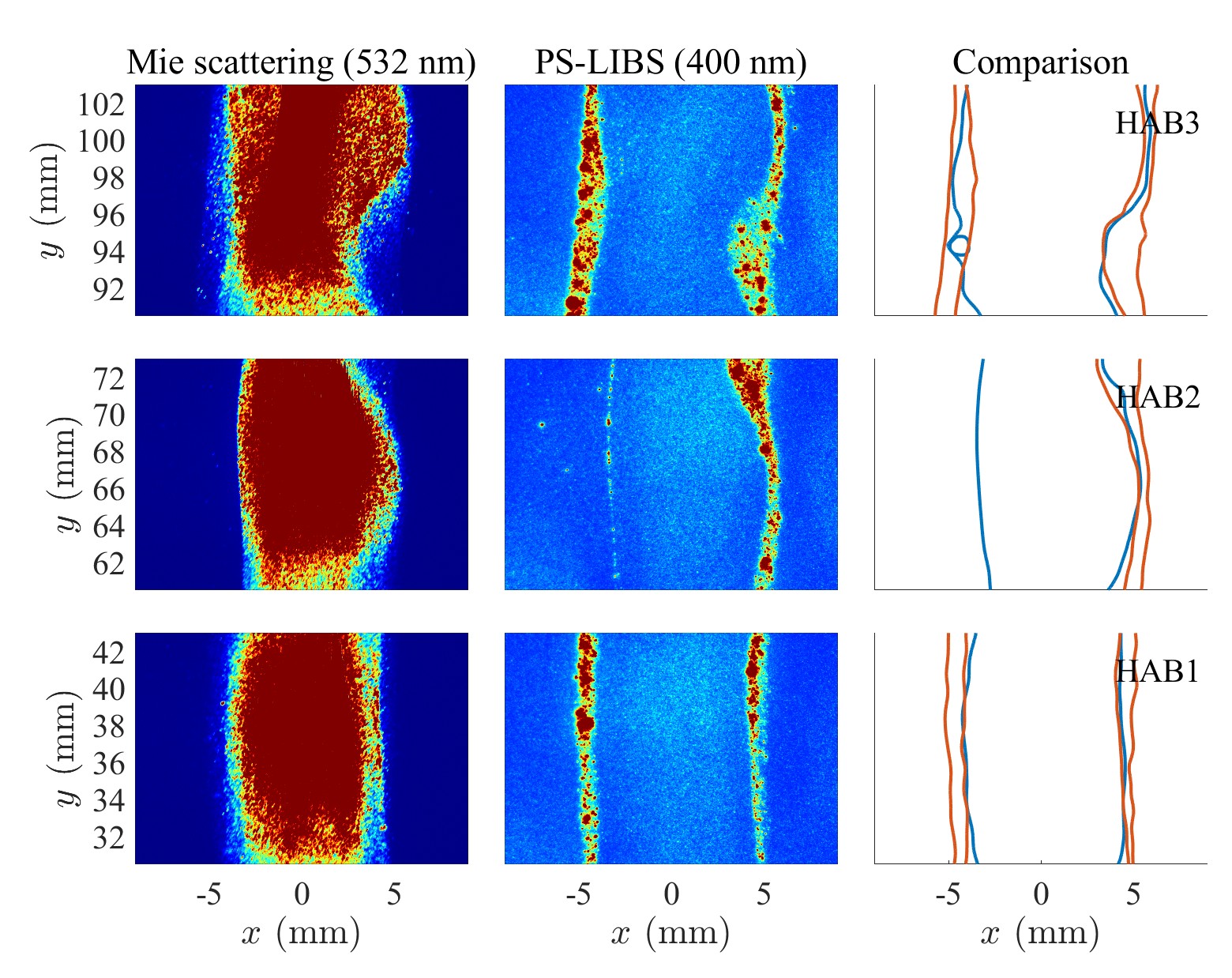

Supplement: Supplementary file 1 — Supplementary material 1 [file 41598_2025_26673_MOESM1_ESM.zip › Fig29.jpg]

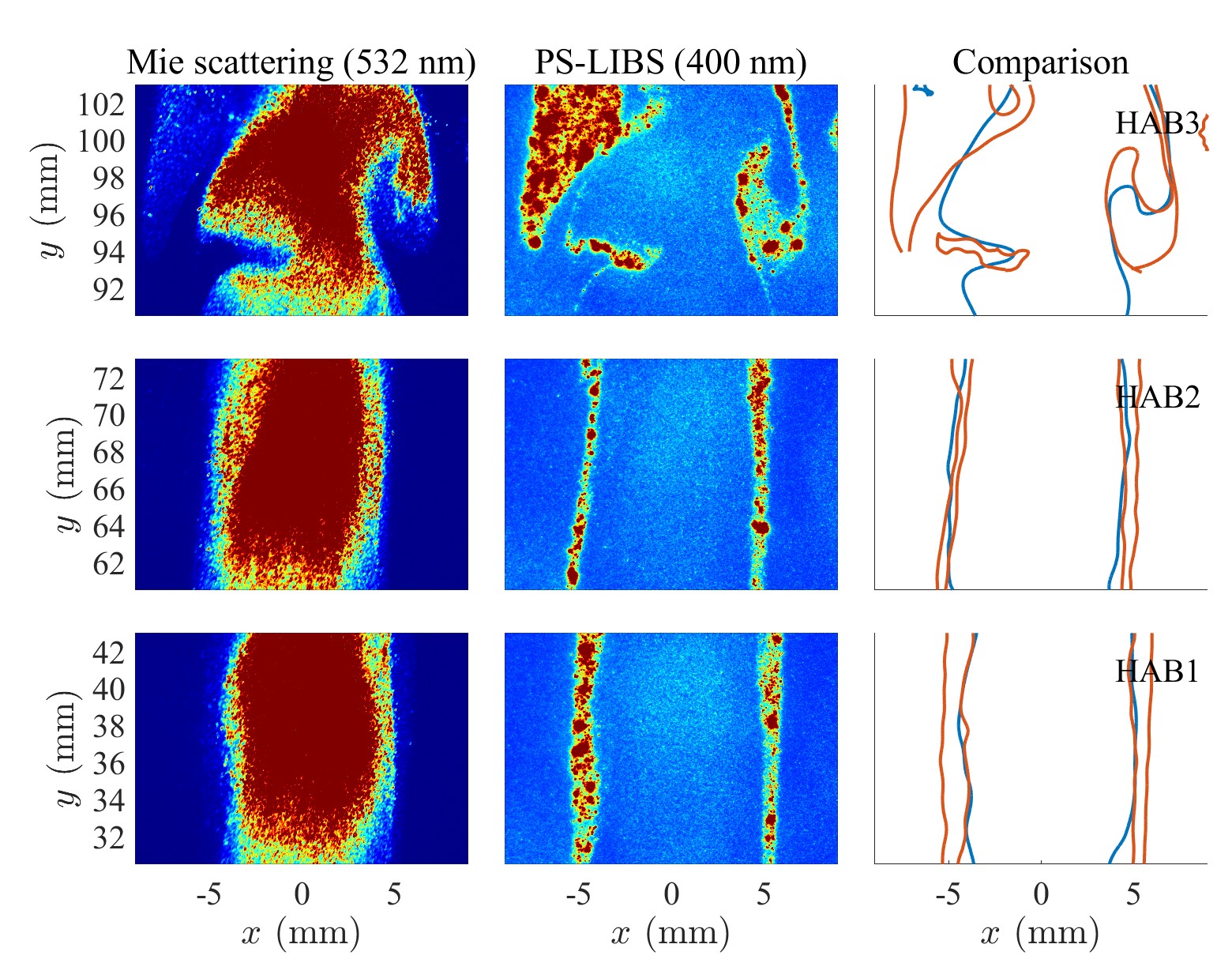

Supplement: Supplementary file 1 — Supplementary material 1 [file 41598_2025_26673_MOESM1_ESM.zip › Fig30.jpg]

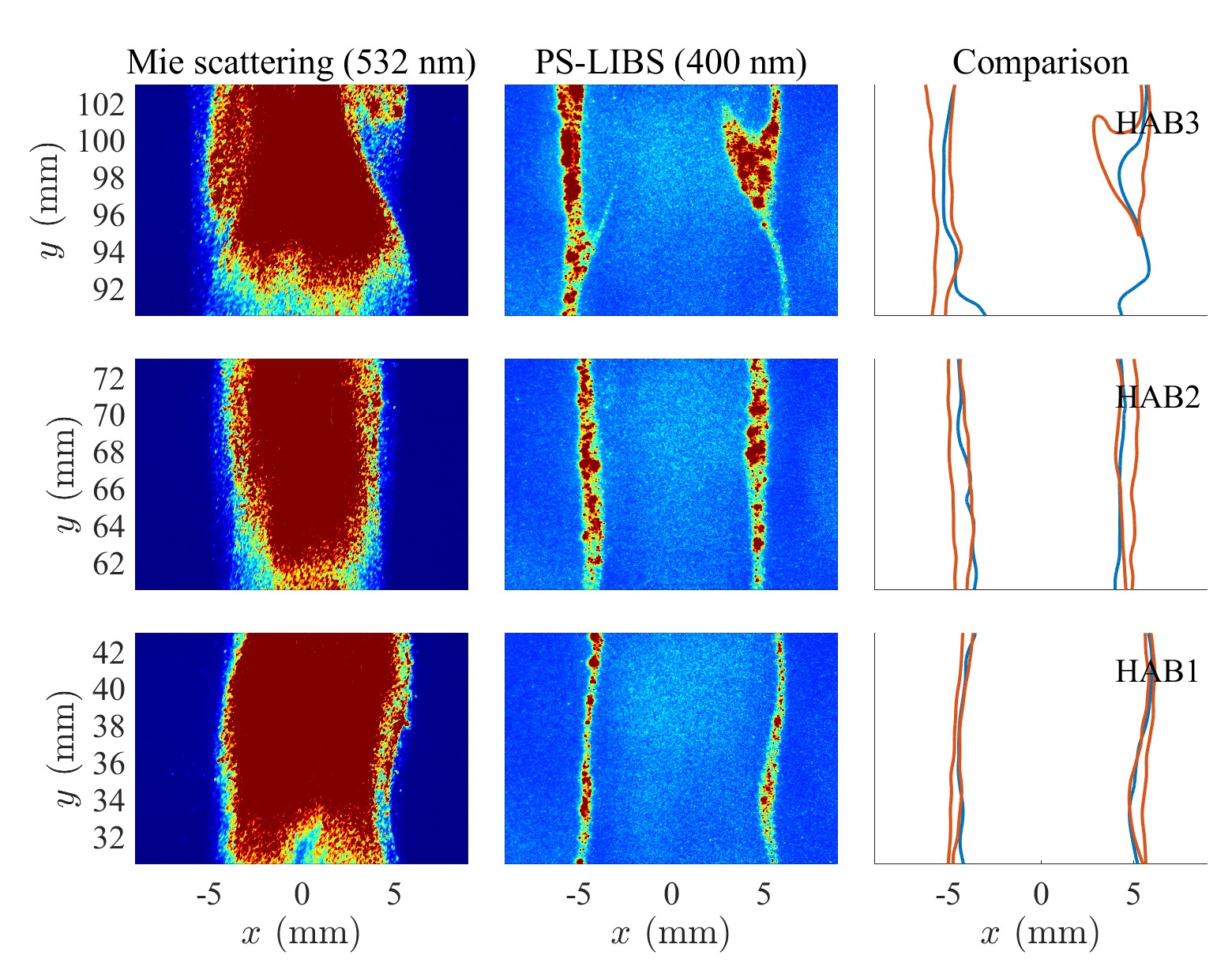

Supplement: Supplementary file 1 — Supplementary material 1 [file 41598_2025_26673_MOESM1_ESM.zip › Fig31.jpg]

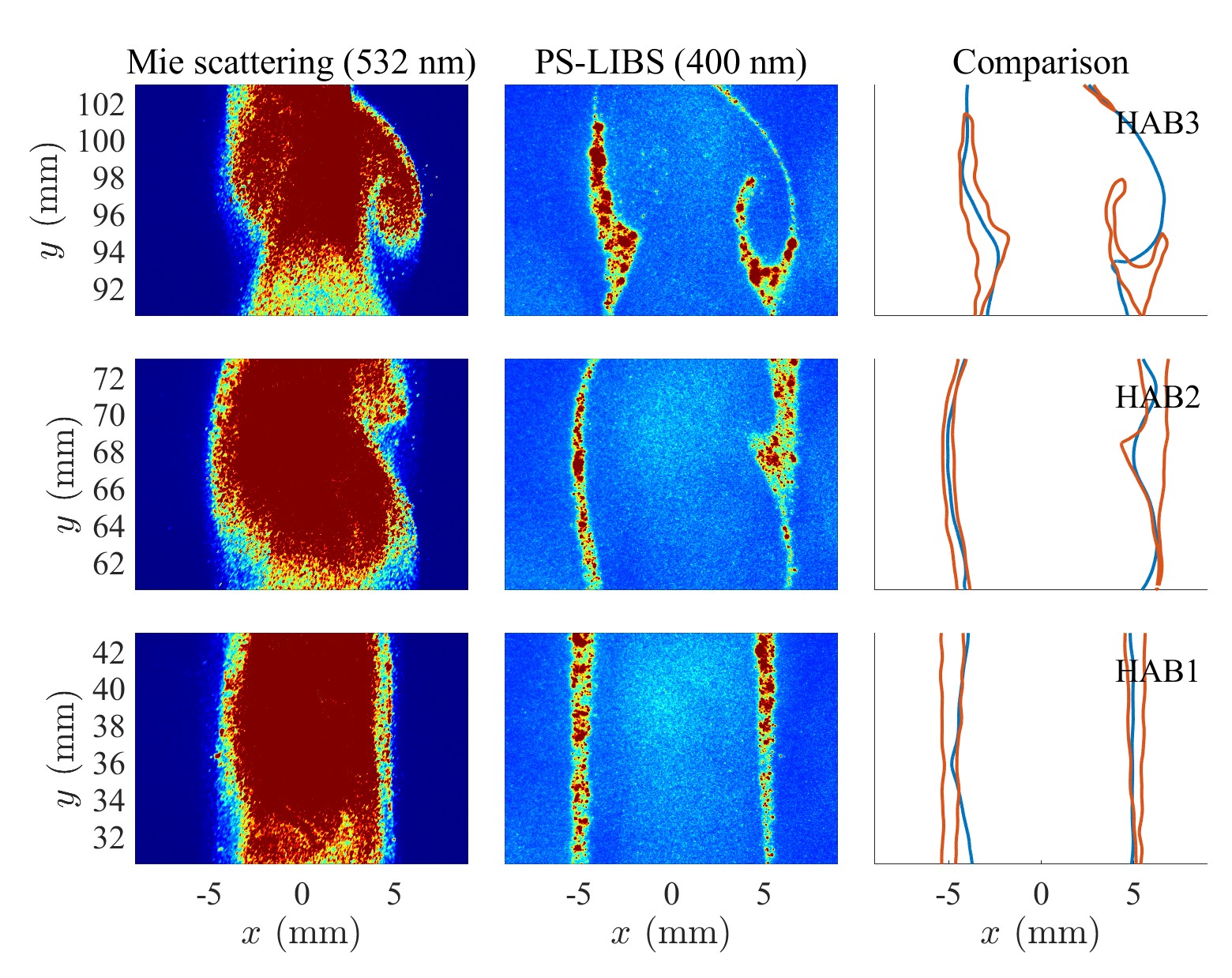

Supplement: Supplementary file 1 — Supplementary material 1 [file 41598_2025_26673_MOESM1_ESM.zip › Fig32.jpg]

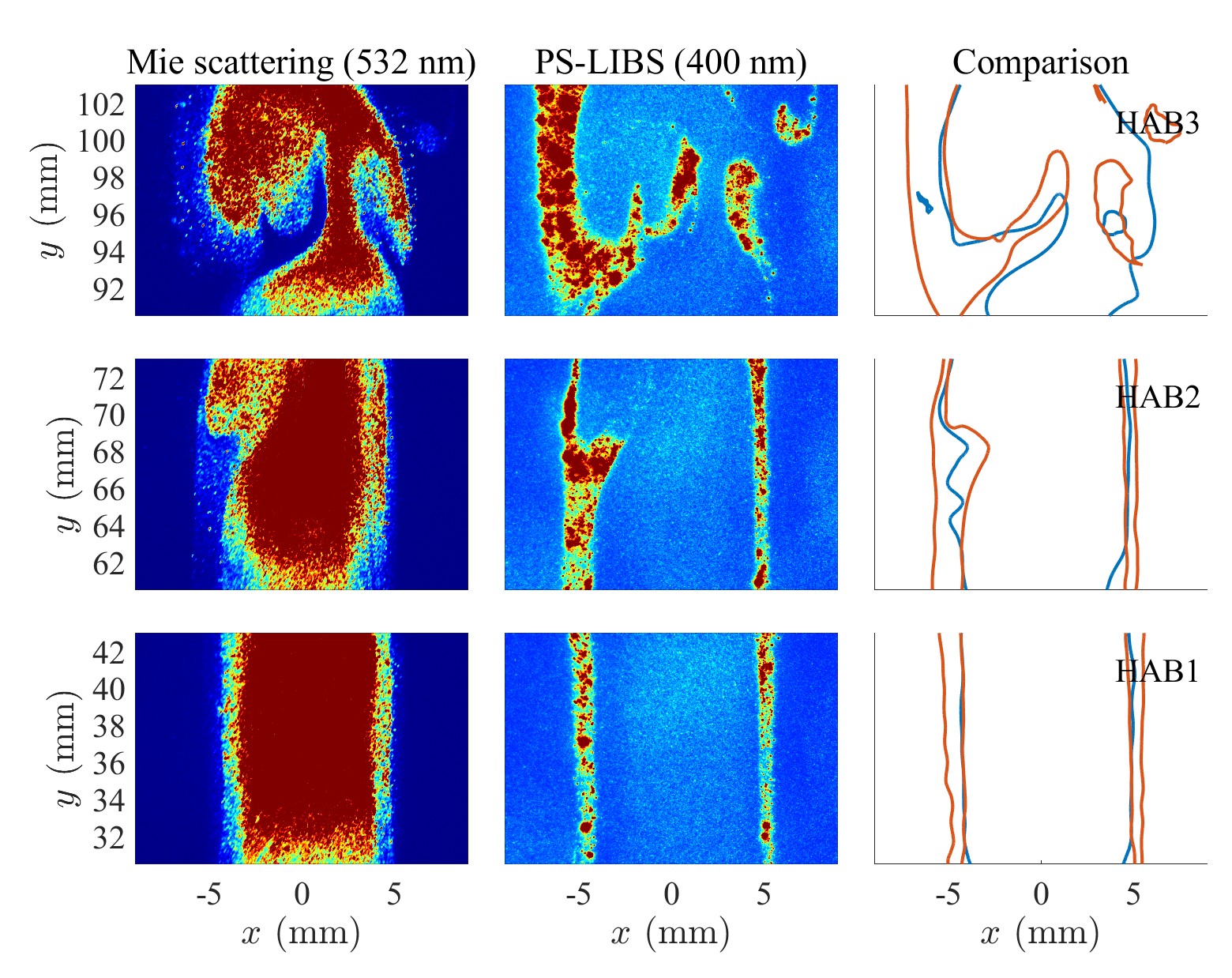

Supplement: Supplementary file 1 — Supplementary material 1 [file 41598_2025_26673_MOESM1_ESM.zip › Fig33.jpg]

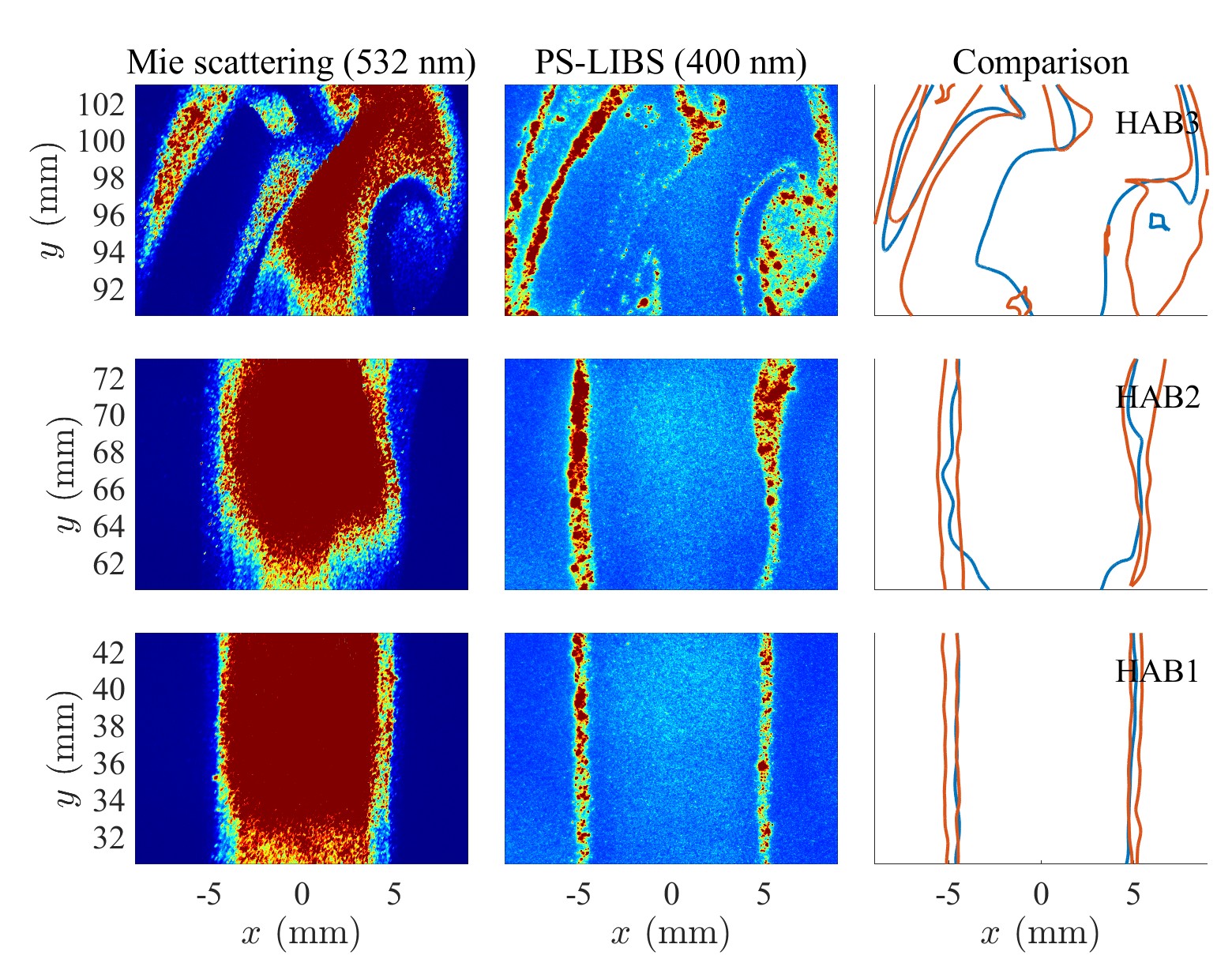

Supplement: Supplementary file 1 — Supplementary material 1 [file 41598_2025_26673_MOESM1_ESM.zip › Fig34.jpg]

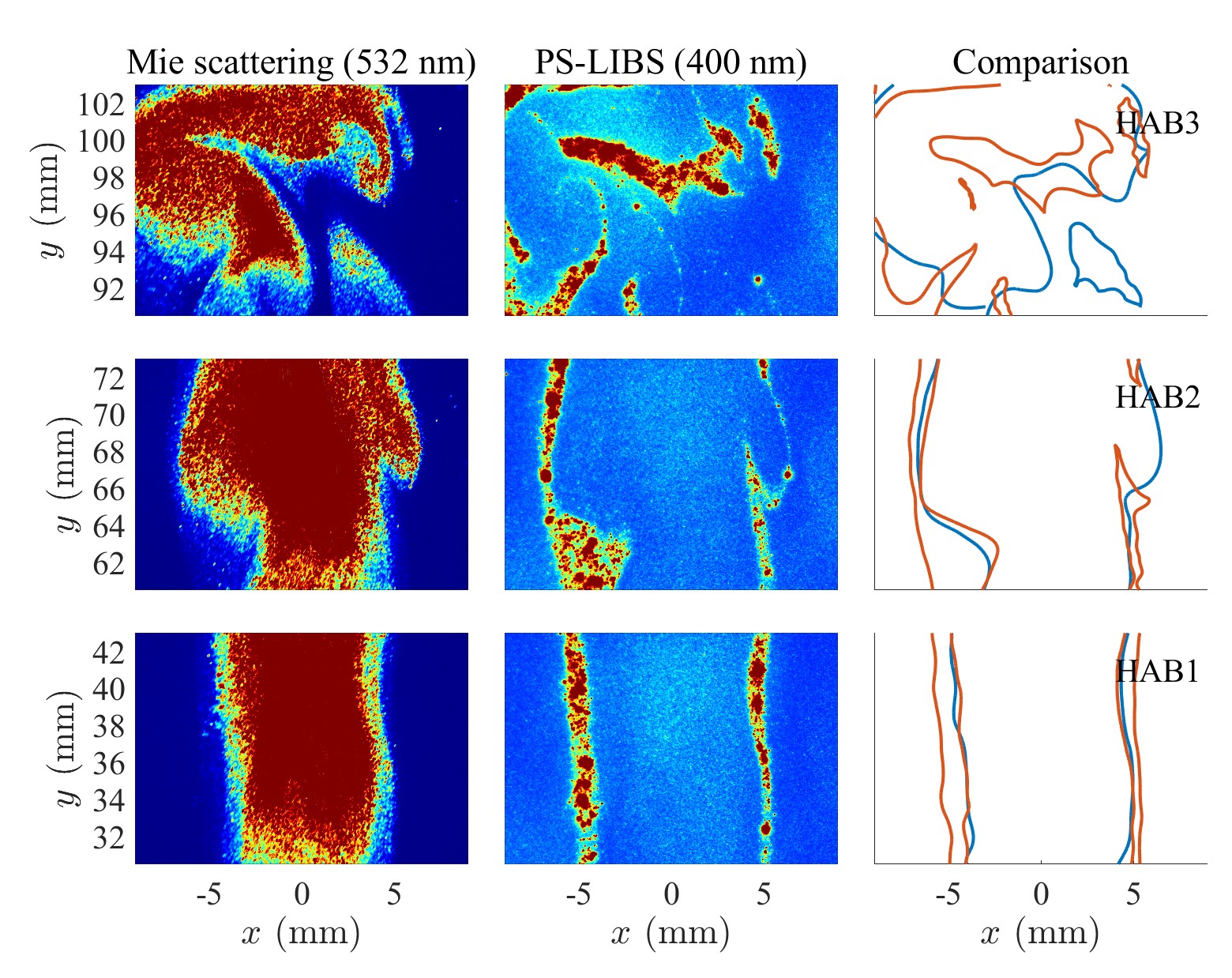

Supplement: Supplementary file 1 — Supplementary material 1 [file 41598_2025_26673_MOESM1_ESM.zip › Fig35.jpg]

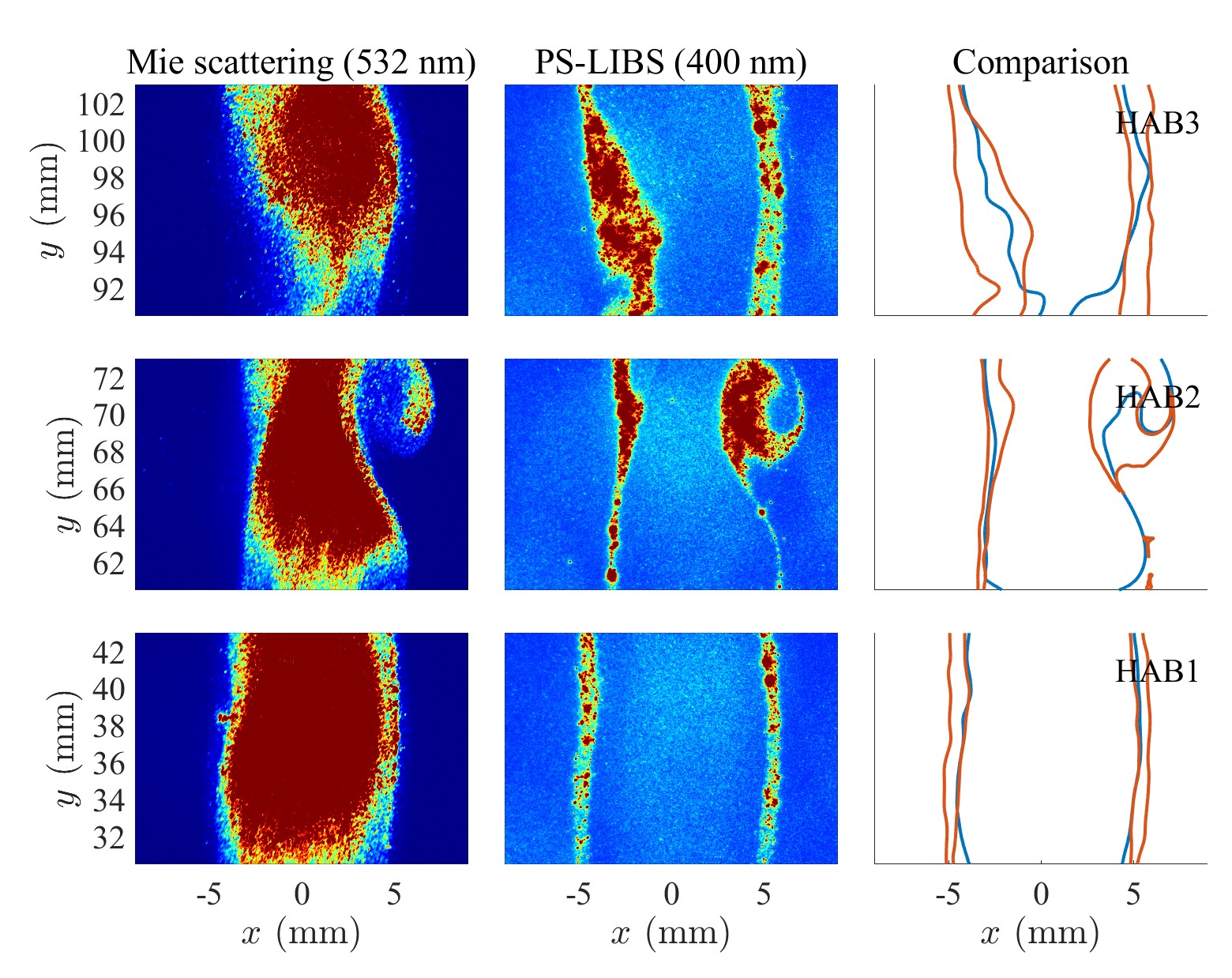

Supplement: Supplementary file 1 — Supplementary material 1 [file 41598_2025_26673_MOESM1_ESM.zip › Fig36.jpg]

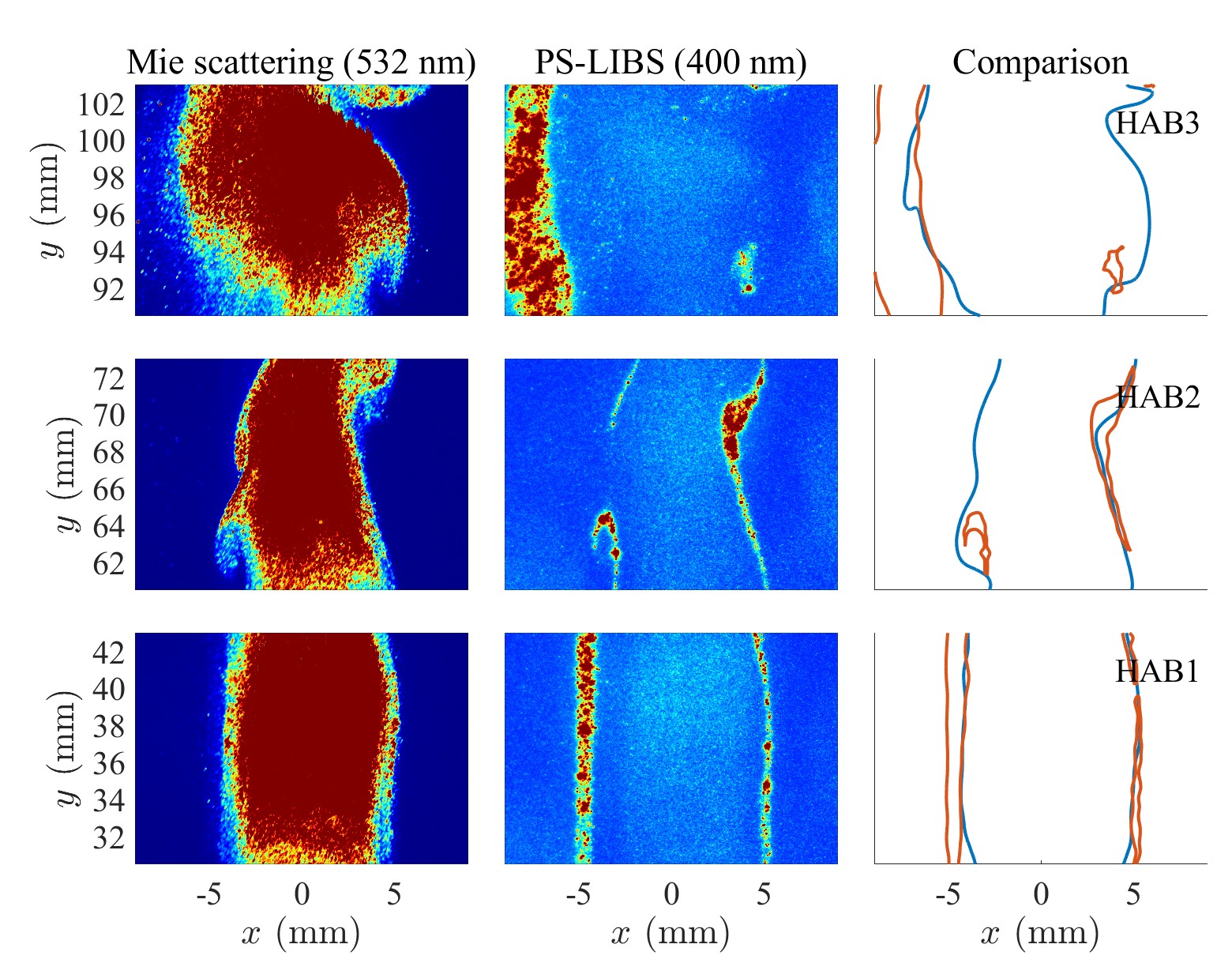

Supplement: Supplementary file 1 — Supplementary material 1 [file 41598_2025_26673_MOESM1_ESM.zip › Fig37.jpg]

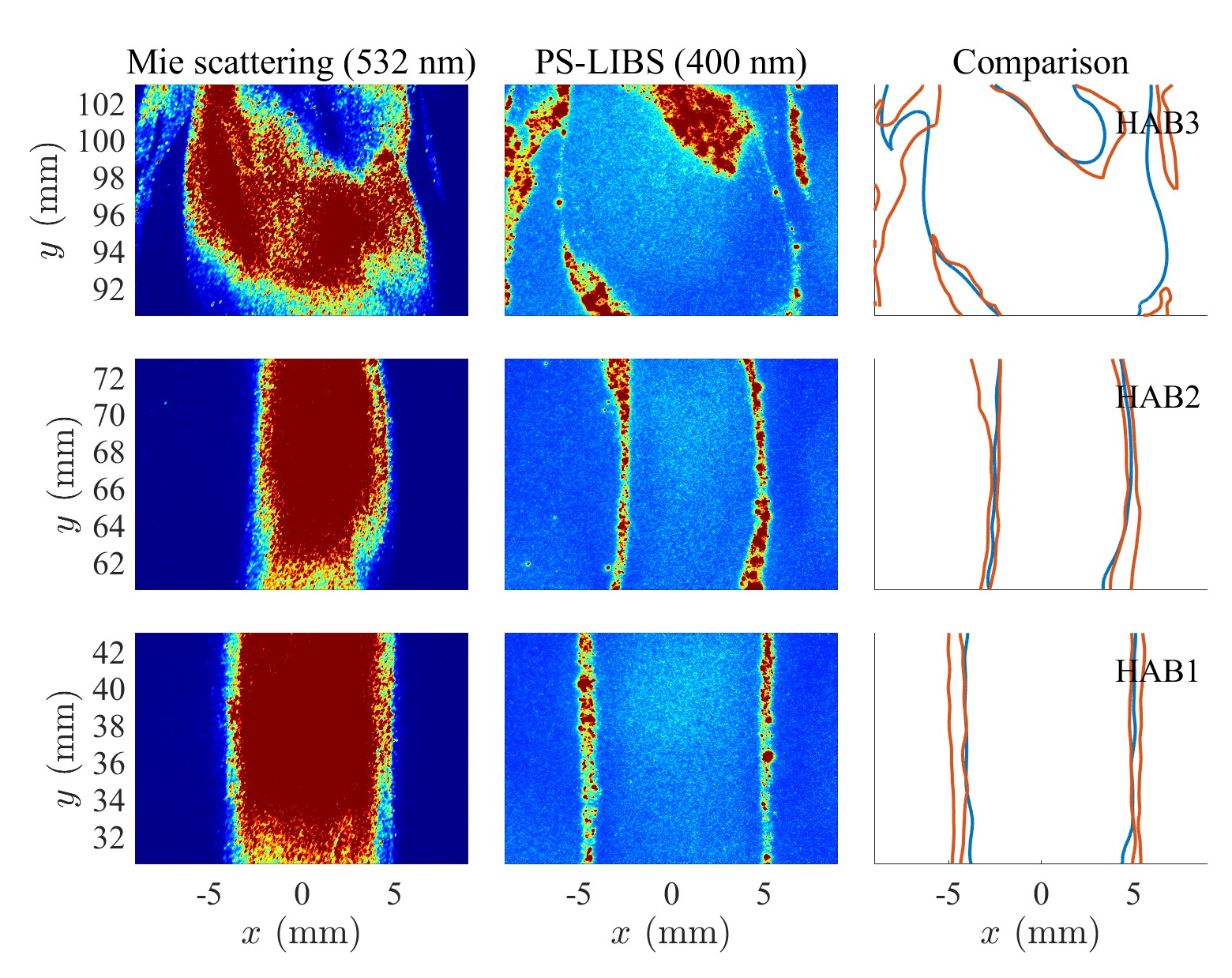

Supplement: Supplementary file 1 — Supplementary material 1 [file 41598_2025_26673_MOESM1_ESM.zip › Fig38.jpg]

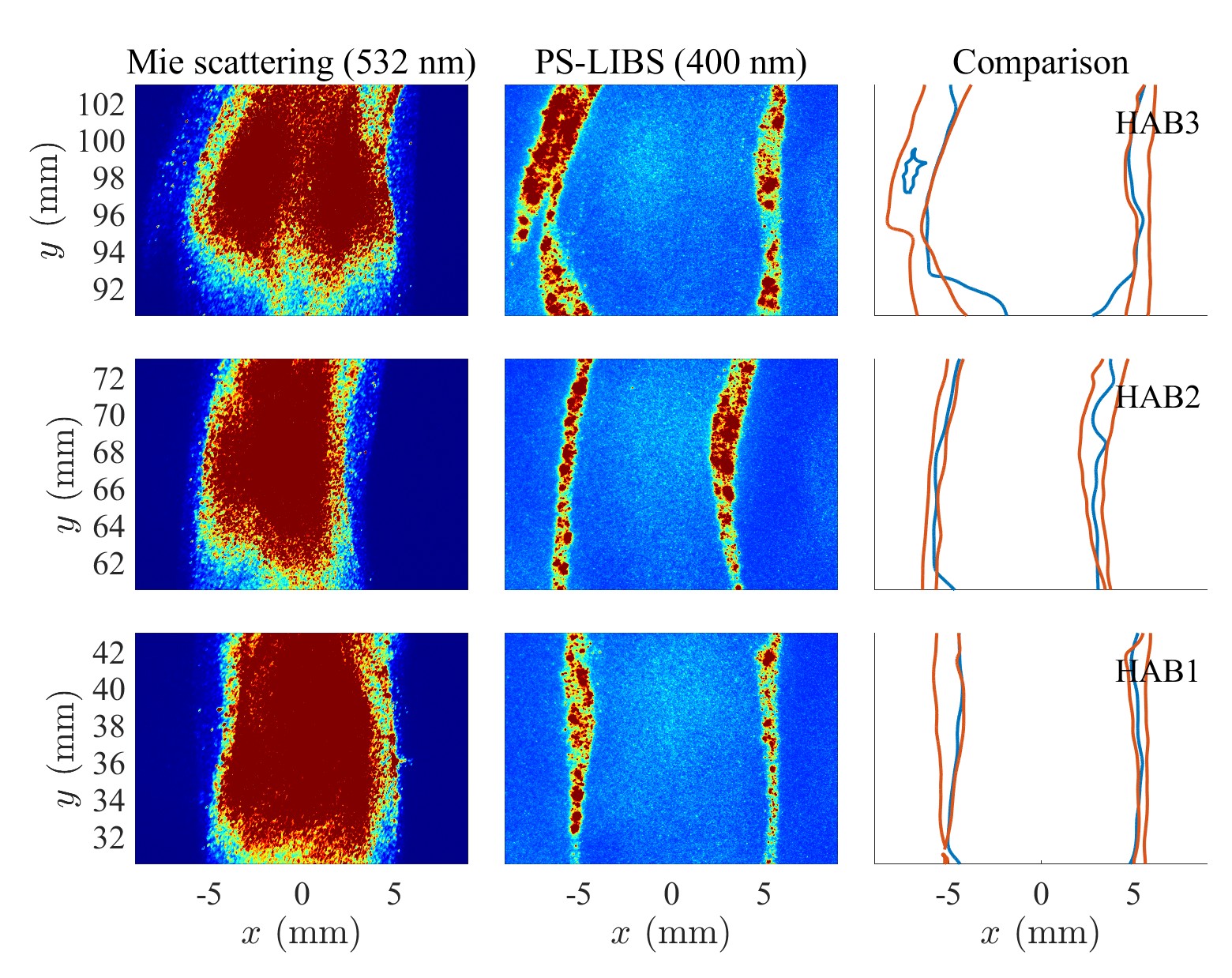

Supplement: Supplementary file 1 — Supplementary material 1 [file 41598_2025_26673_MOESM1_ESM.zip › Fig39.jpg]

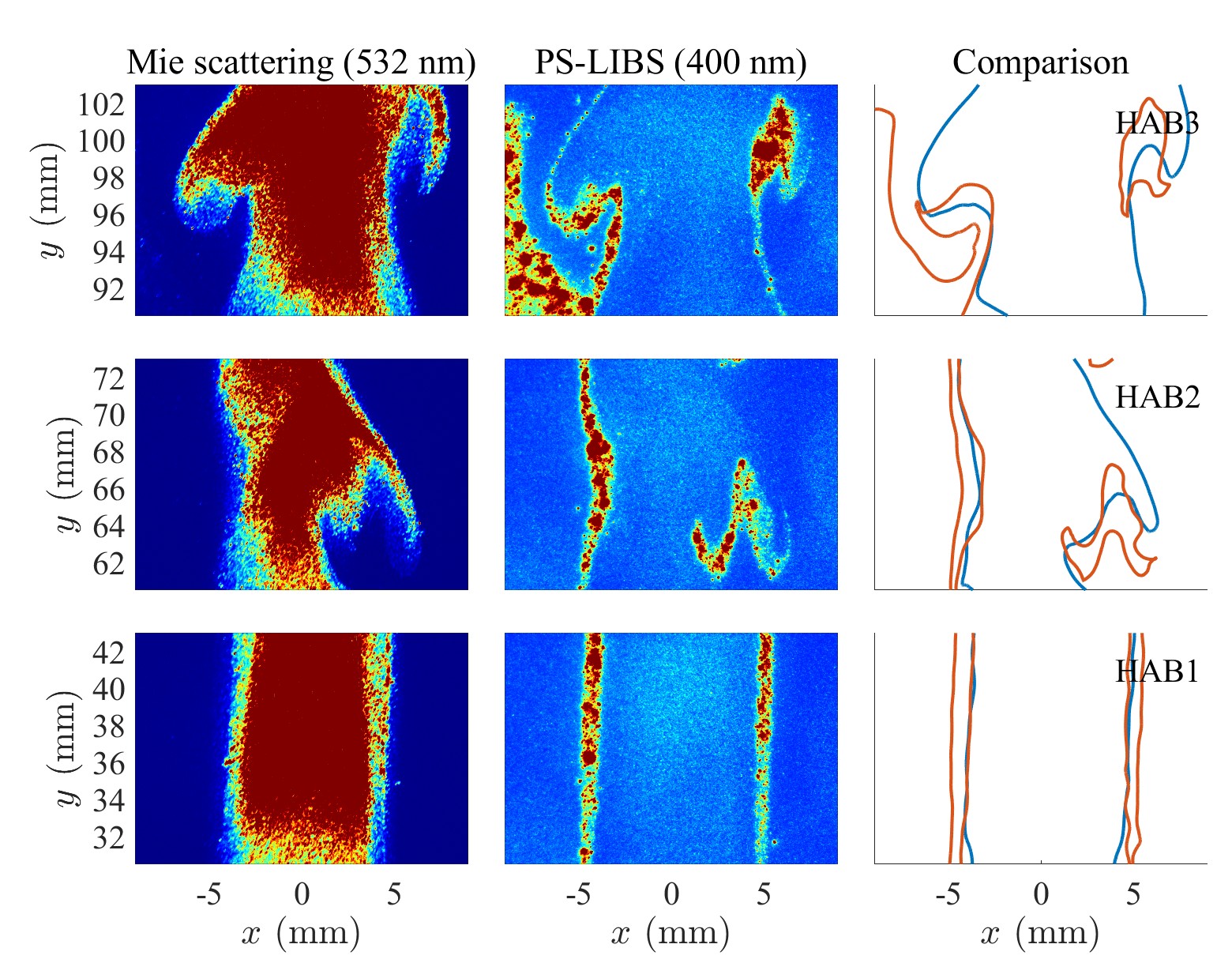

Supplement: Supplementary file 1 — Supplementary material 1 [file 41598_2025_26673_MOESM1_ESM.zip › Fig40.jpg]

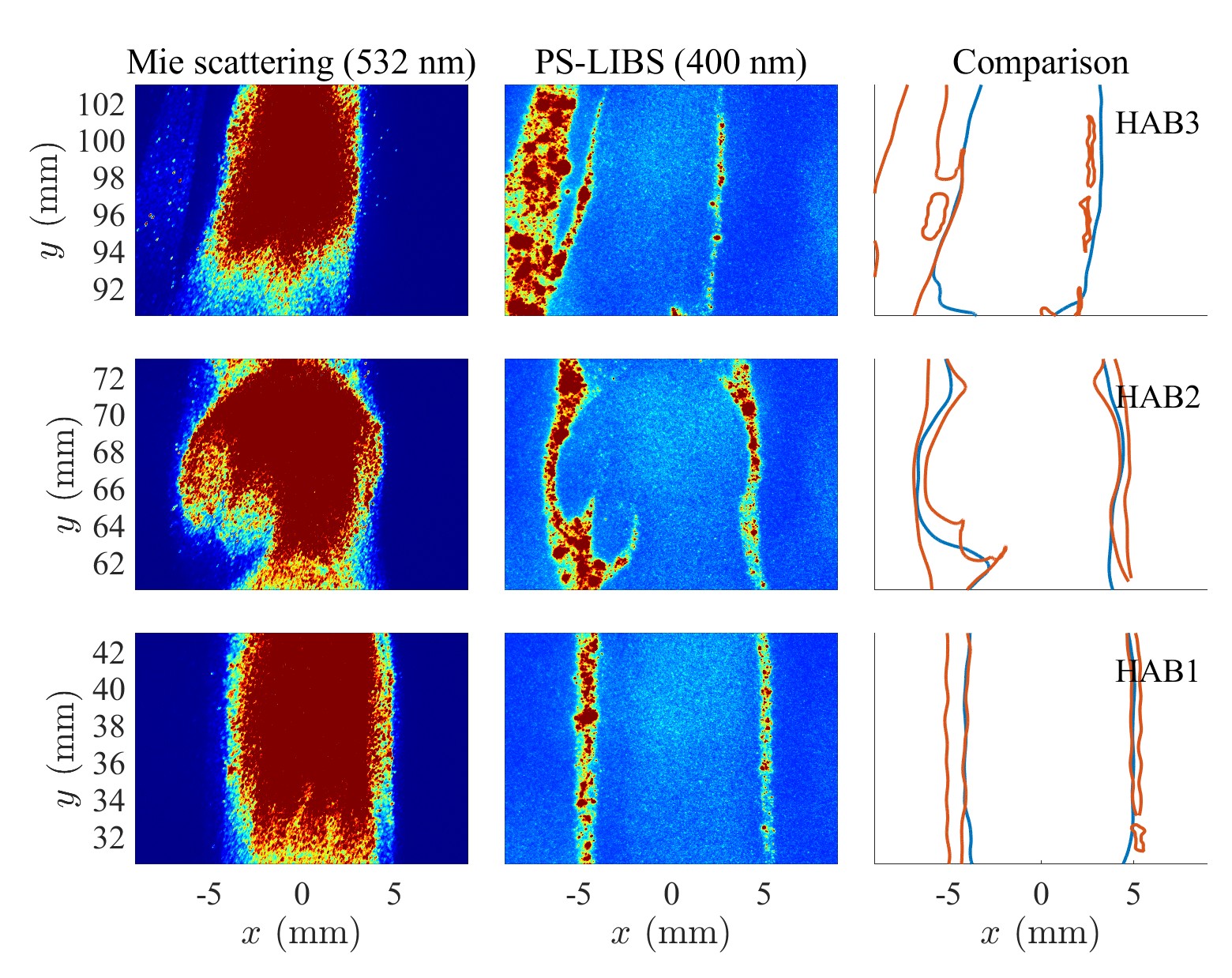

Supplement: Supplementary file 1 — Supplementary material 1 [file 41598_2025_26673_MOESM1_ESM.zip › Fig41.jpg]

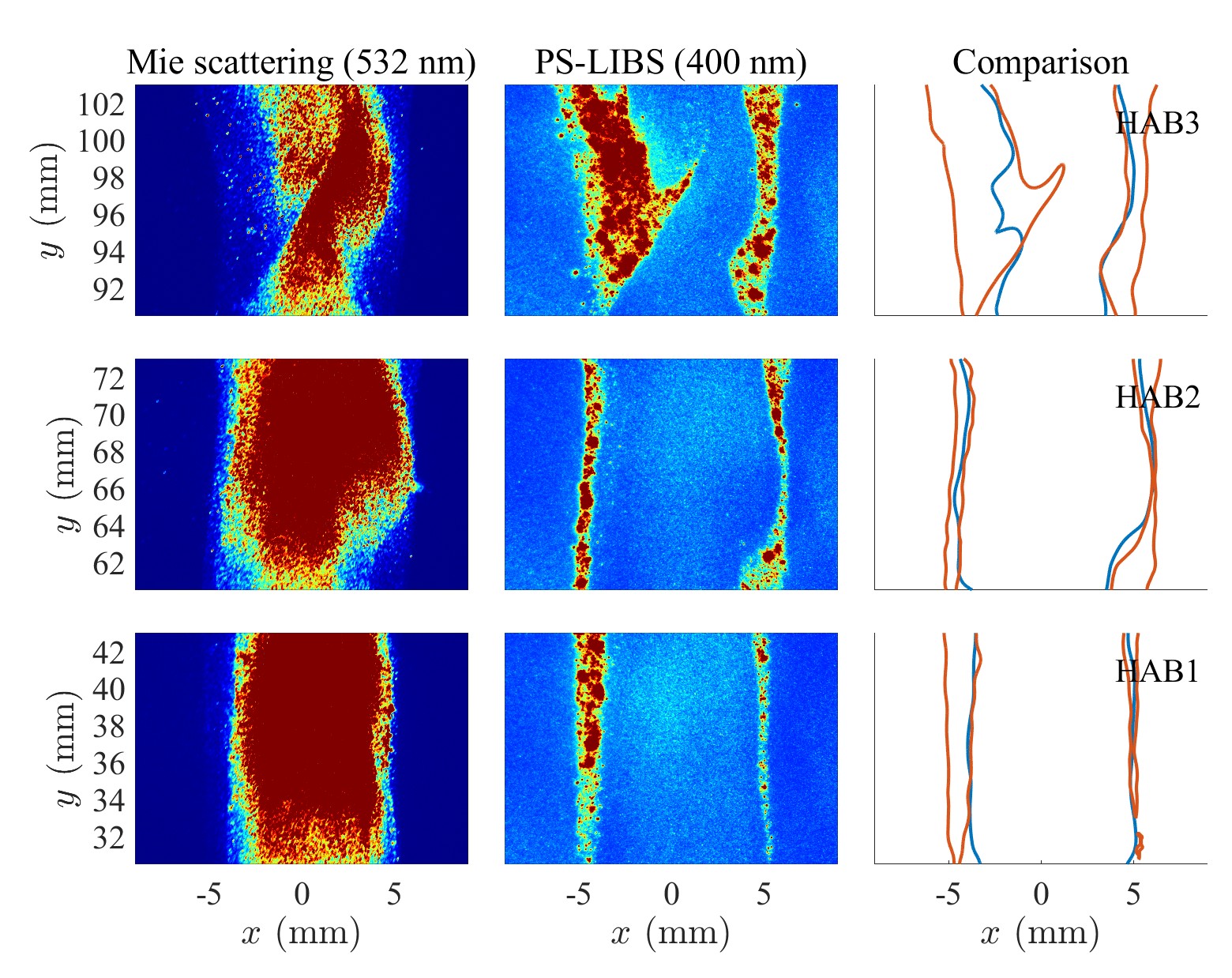

Supplement: Supplementary file 1 — Supplementary material 1 [file 41598_2025_26673_MOESM1_ESM.zip › Fig42.jpg]

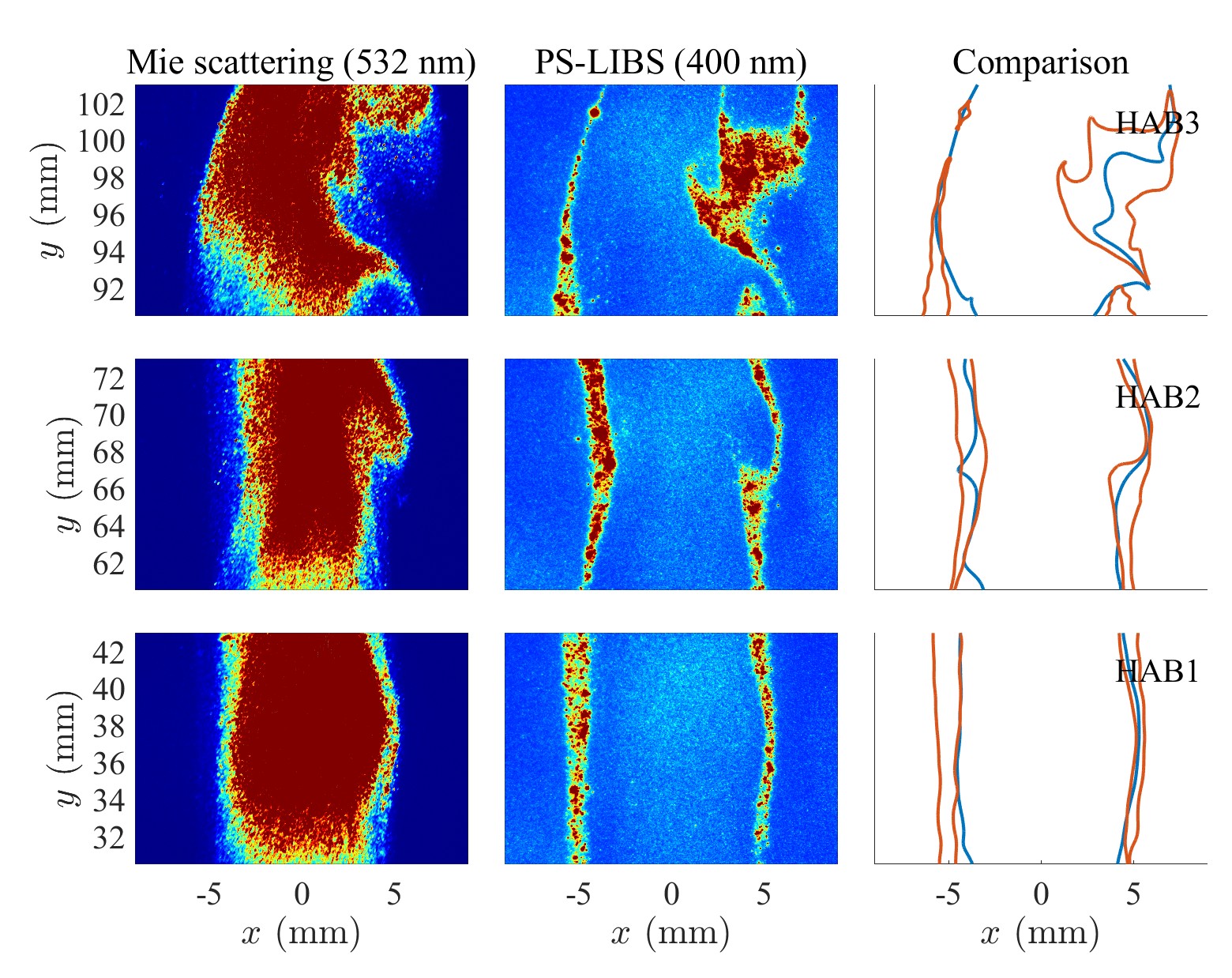

Supplement: Supplementary file 1 — Supplementary material 1 [file 41598_2025_26673_MOESM1_ESM.zip › Fig43.jpg]

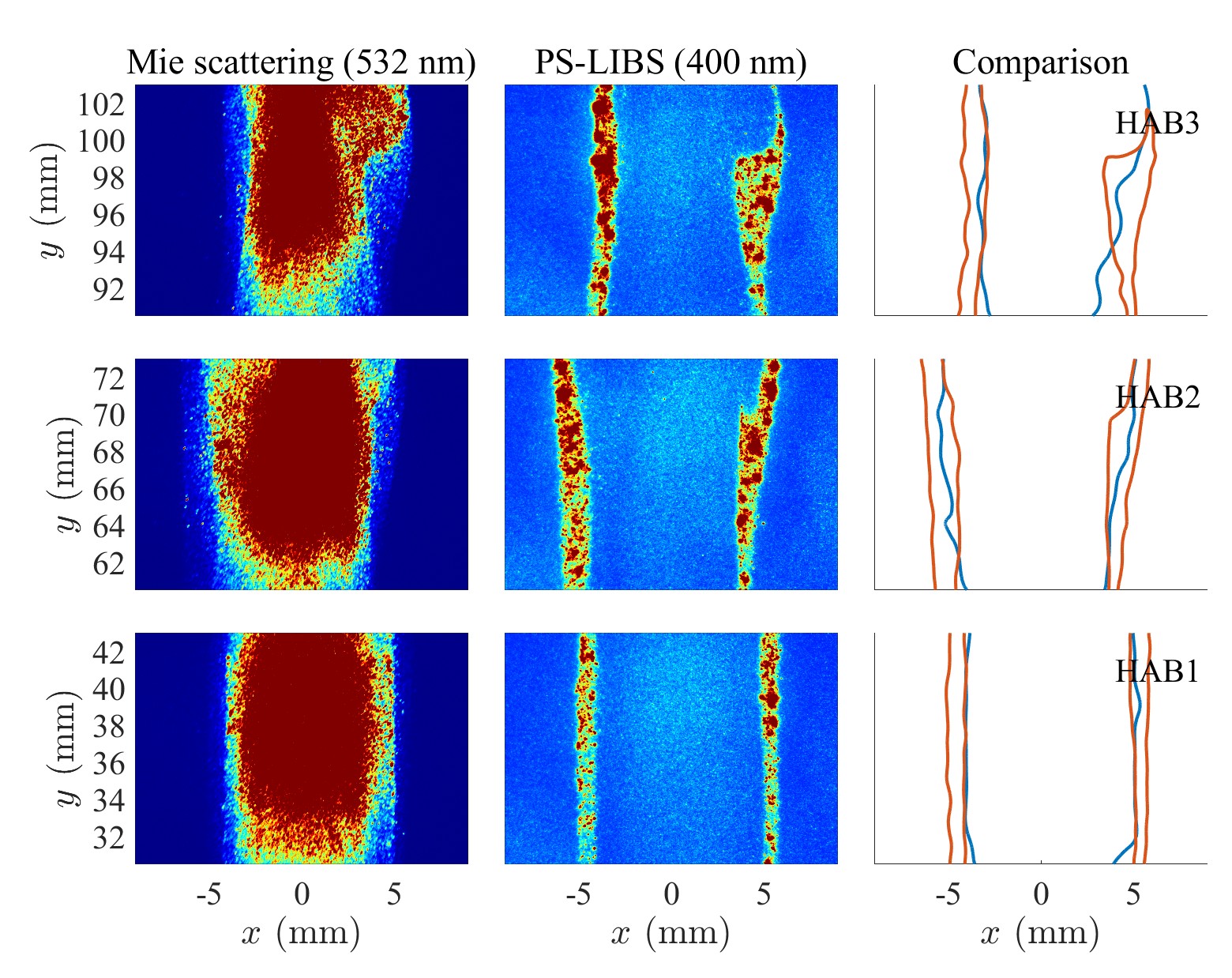

Supplement: Supplementary file 1 — Supplementary material 1 [file 41598_2025_26673_MOESM1_ESM.zip › Fig44.jpg]

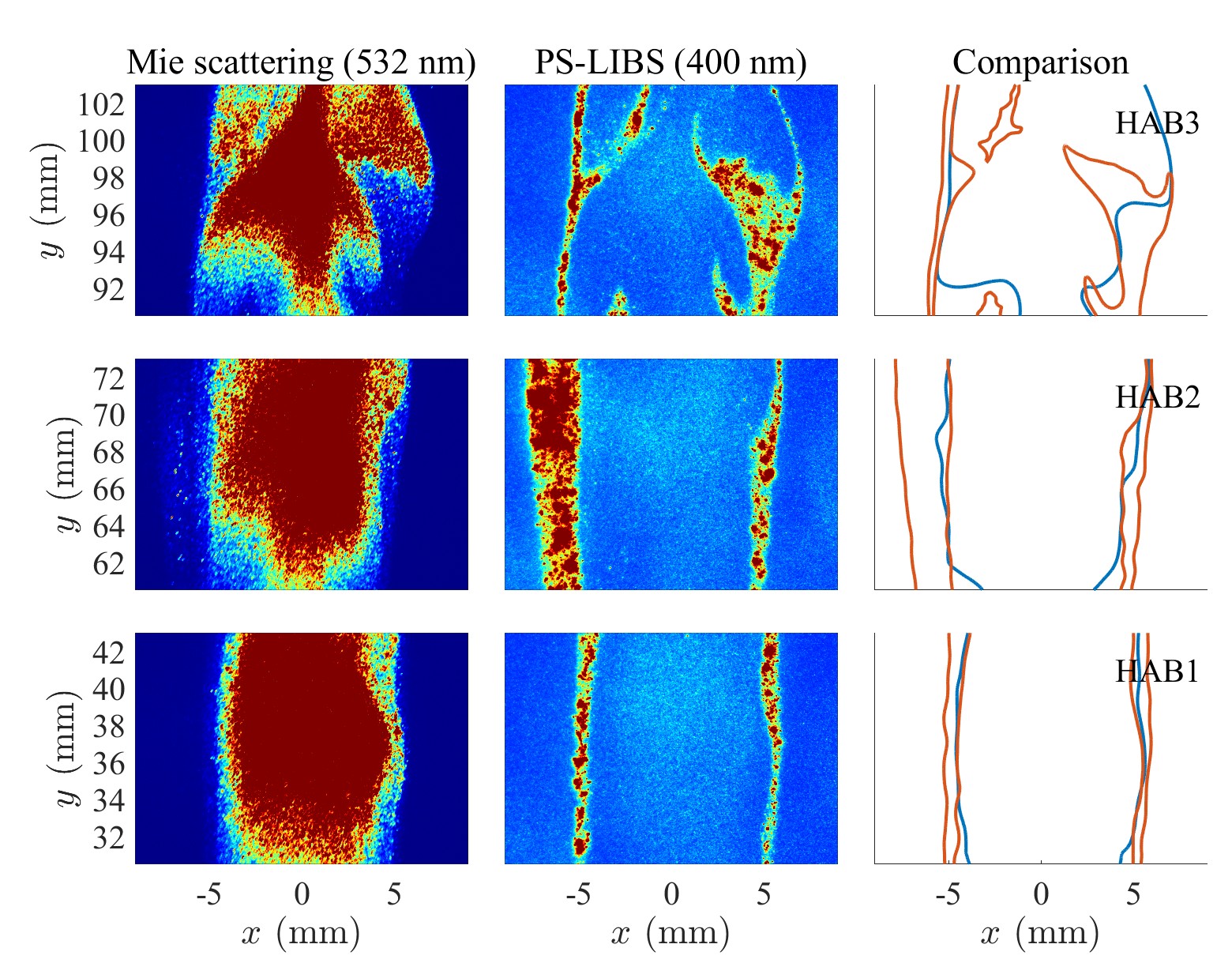

Supplement: Supplementary file 1 — Supplementary material 1 [file 41598_2025_26673_MOESM1_ESM.zip › Fig45.jpg]

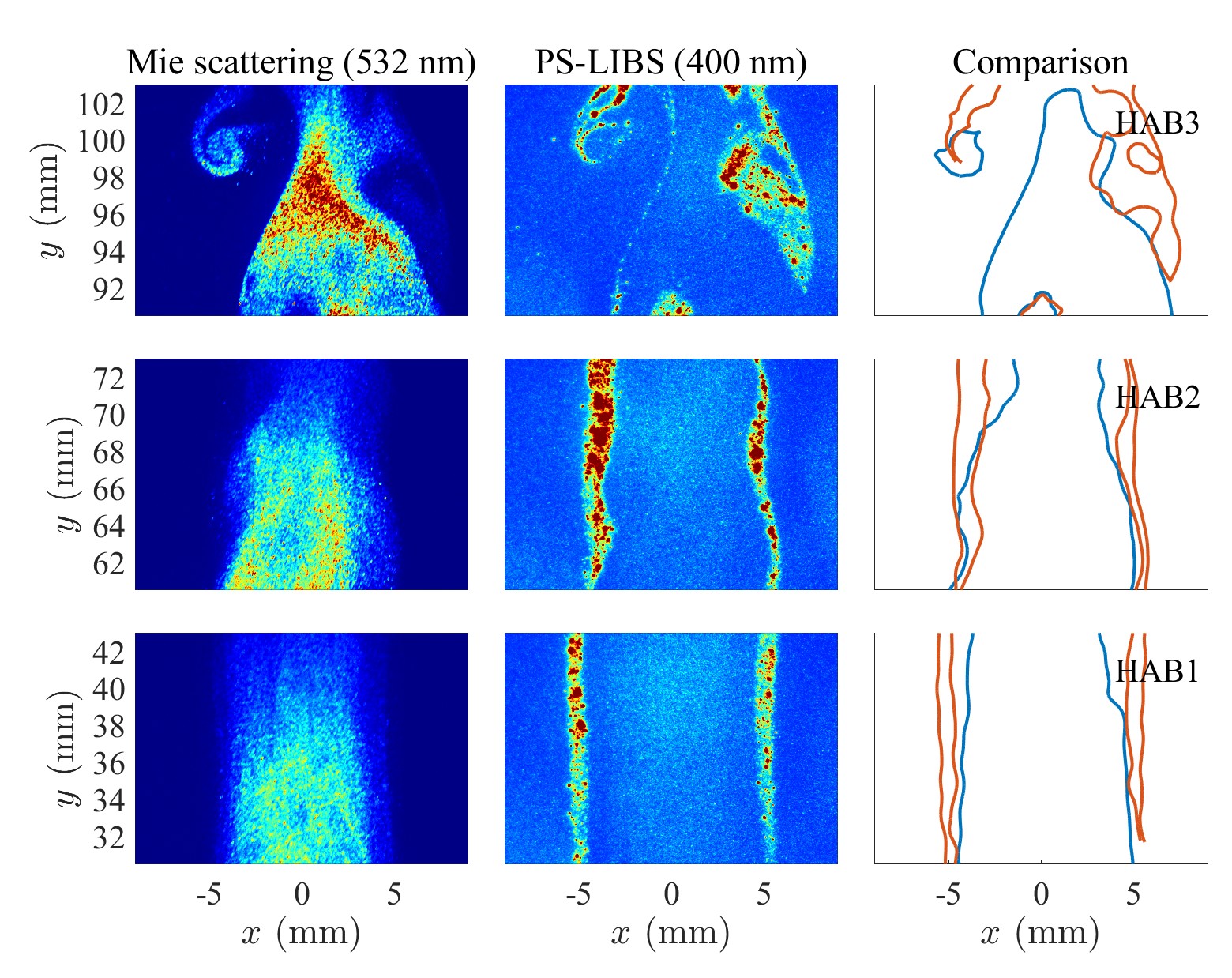

Supplement: Supplementary file 1 — Supplementary material 1 [file 41598_2025_26673_MOESM1_ESM.zip › Fig46.jpg]

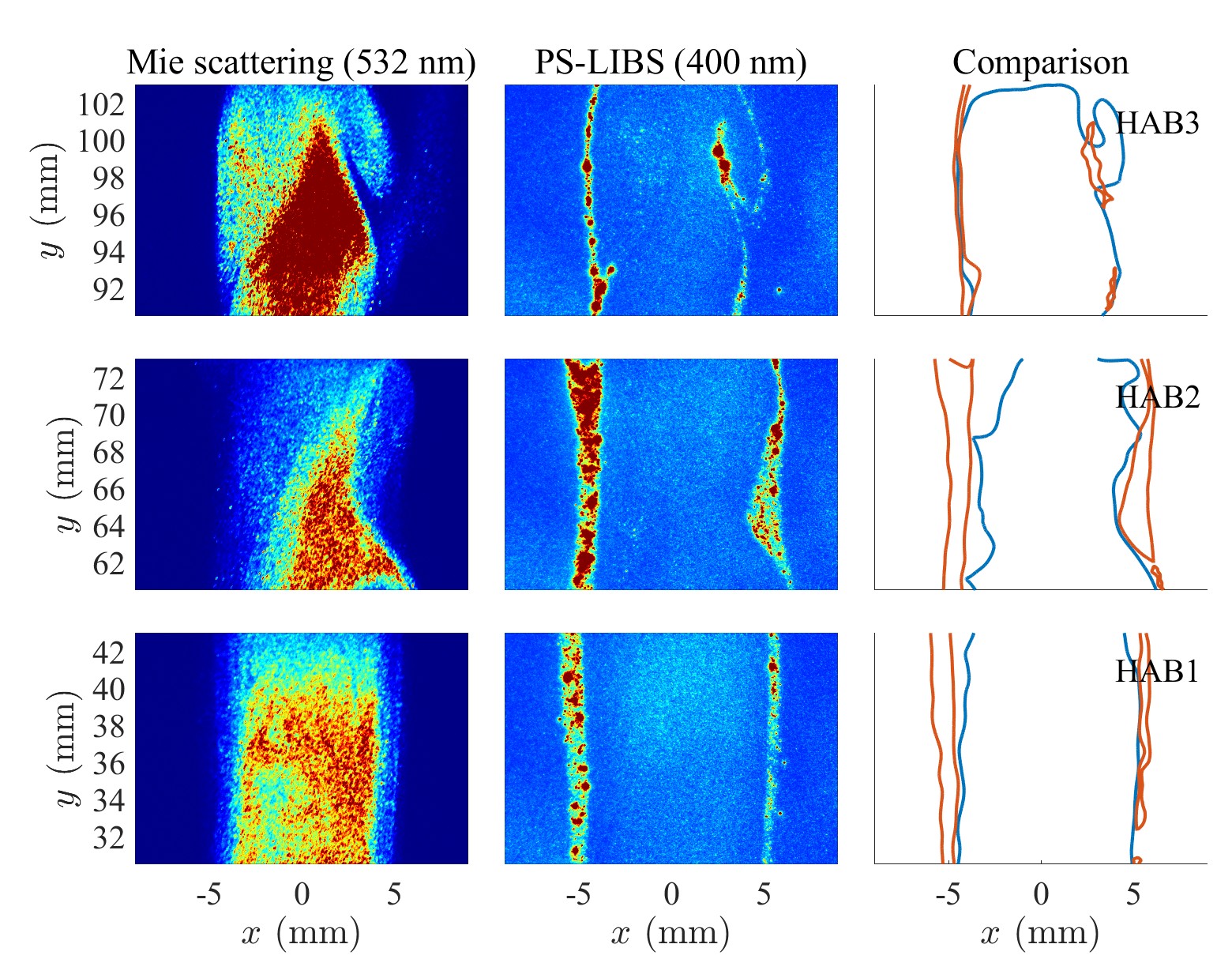

Supplement: Supplementary file 1 — Supplementary material 1 [file 41598_2025_26673_MOESM1_ESM.zip › Fig47.jpg]

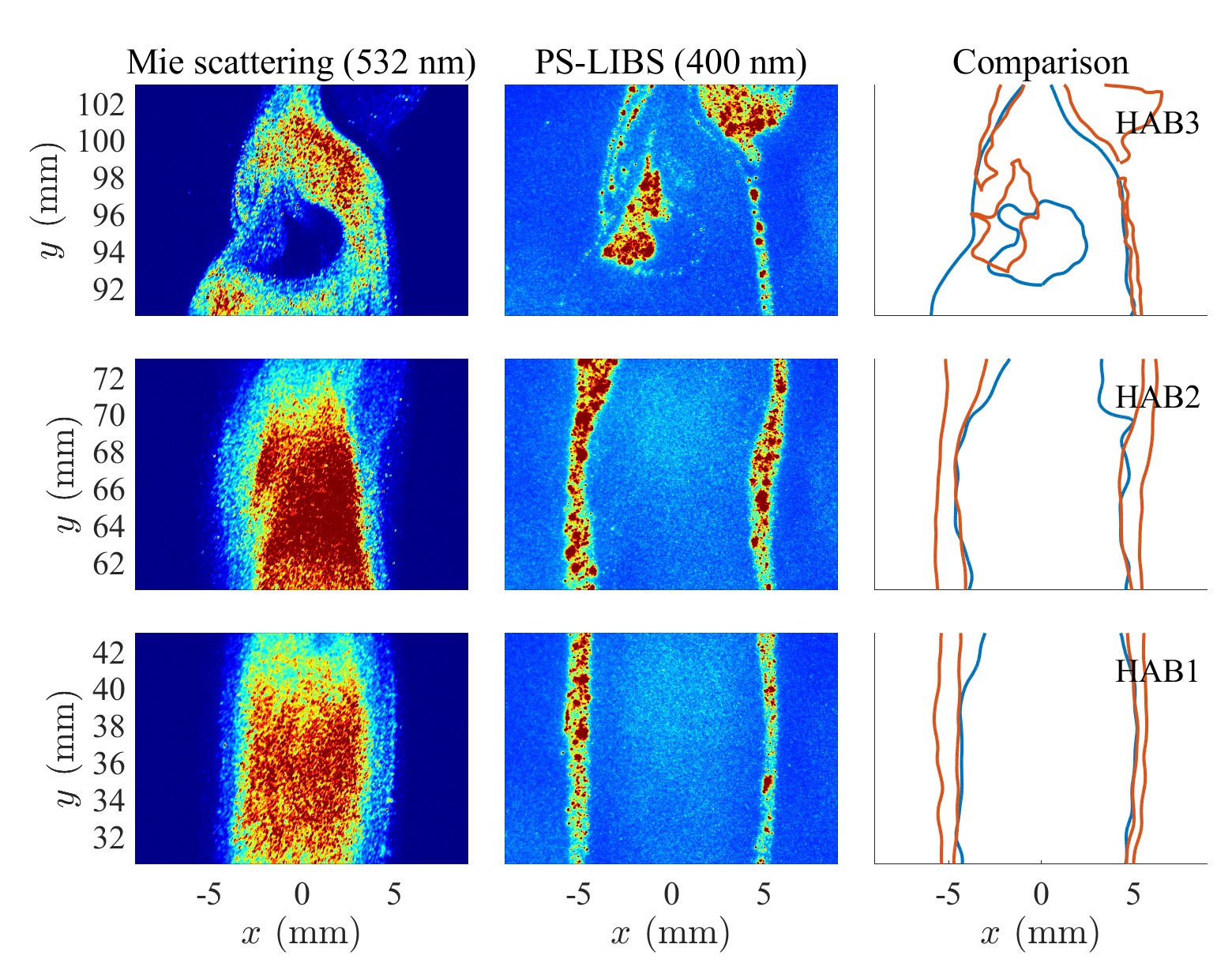

Supplement: Supplementary file 1 — Supplementary material 1 [file 41598_2025_26673_MOESM1_ESM.zip › Fig48.jpg]
